# Metabolite Profiling and Dipeptidyl Peptidase IV Inhibitory Activity of *Coreopsis* Cultivars in Different Mutations

**DOI:** 10.3390/plants10081661

**Published:** 2021-08-12

**Authors:** Bo-Ram Kim, Sunil Babu Paudel, Ah-Reum Han, Jisu Park, Yun-Seo Kil, Hyukjae Choi, Yeo Gyeong Jeon, Kong Young Park, Si-Yong Kang, Chang Hyun Jin, Jin-Baek Kim, Joo-Won Nam

**Affiliations:** 1 Advanced Radiation Technology Institute, Korea Atomic Energy Research Institute, Jeongeup-si 56212, Jeollabuk-do, Korea; boram0307@hnibr.re.kr (B.-R.K.); arhan@kaeri.re.kr (A.-R.H.); parksj94@kaeri.re.kr (J.P.); chjin@kaeri.re.kr (C.H.J.); jbkim74@kaeri.re.kr (J.-B.K.); 2 Natural Product Research Division, Honam National Institute of Biological Resources, Mokpo-si 58762, Jeollanam-do, Korea; 3 College of Pharmacy, Yeungnam University, Gyeongsan-si 38541, Gyeongsangbuk-do, Korea; phrsunil@gmail.com (S.B.P.); yskil@yu.ac.kr (Y.-S.K.); h5choi@yu.ac.kr (H.C.); 4 Research Institute of Cell Culture, Yeungnam University, Gyeongsan 38541, Gyeongbuk, Korea; 5 Uriseed Group, Icheon-si 17408, Gyeonggi-do, Korea; ygjeon@uriseed.com (Y.G.J.); uriseeds@naver.com (K.Y.P.); 6 Department of Horticulture, College of Industrial Sciences, Kongju National University, Yesan-gun 32439, Chungcheongnam-do, Korea; sykang@kongju.ac.kr

**Keywords:** *Coreopsis rosea*, *Coreopsis verticillata*, mutant cultivar, metabolomics, dipeptidyl peptidase-IV

## Abstract

*Coreopsis* species have been developed to produce cultivars of various floral colors and sizes and are also used in traditional medicine. To identify and evaluate mutant cultivars of *C*. *rosea* and *C*. *verticillata*, their phytochemical profiles were systematically characterized using ultra-performance liquid chromatography time-of-flight mass spectrometry, and their anti-diabetic effects were evaluated using the dipeptidyl peptidase (DPP)-IV inhibitor screening assay. Forty compounds were tentatively identified. This study is the first to provide comprehensive chemical information on the anti-diabetic effect of *C*. *rosea* and *C*. *verticillata*. All 32 methanol extracts of *Coreopsis* cultivars inhibited DPP-IV activity in a concentration-dependent manner (IC_50_ values: 34.01–158.83 μg/mL). Thirteen compounds presented as potential markers for distinction among the 32 *Coreopsis* cultivars via principal component analysis and orthogonal partial least squares discriminant analysis. Therefore, these bio-chemometric models can be useful in distinguishing cultivars as potential dietary supplements for functional plants.

## 1. Introduction

*Coreopsis* species are annual or perennial plants belonging to the Asteraceae (Compositae) family [1]. Approximately 80 species of *Coreopsis* are native to North America and are currently widespread in America, Asia, and Oceania regions [2,3,4]. They are usually cultivated for ornamental purposes in gardens or on roadsides. The plants are in the range of 46–120 cm in height and the petals of the flowers are primarily yellow in color and are serrated [5,6]. The color and size of *Coreopsis* flowers have commercially important value and are the reason for *Coreopsis* breeding. In addition, the *Coreopsis* flower has been ethnopharmacologically used for the treatment of diarrhea, vomiting, and hemorrhage in North America, where the *Coreopsis* species originates [7,8]. It has also been used as a drink to control diabetes in China and Portugal, and as an herbal tea to eliminate toxins and fever from the body in China [8, 9,10]. Nowadays, owing to scientific proof of its traditional use, several studies have been conducted on the phytochemical and biological activities of *C*. *lanceolata* and *C*. *tinctoria*, in particular [2,3,4,5,6,7,8,9,10,11,12,13,14,15,16,17,18,19]. Diverse types of flavonoids, such as aurone, chalcone, flavanone, and flavanol have been identified from *C*. *lanceolata* and *C*. *tinctoria*. In addition, unique polyacetylene compounds have also been found in these plants [10,12,13]. Various pharmacological activities such as anticancer [2,5], antioxidant [6,14,15,16,17,18], anti-inflammatory [6,10,19], and anti-diabetic effects [8,12] have been reported of compounds isolated from *C*. *lanceolata* and *C*. *tinctoria*. Limited reports on the chemical composition and biological activities of *Coreopsis* species exist. 

**Figure 1:**
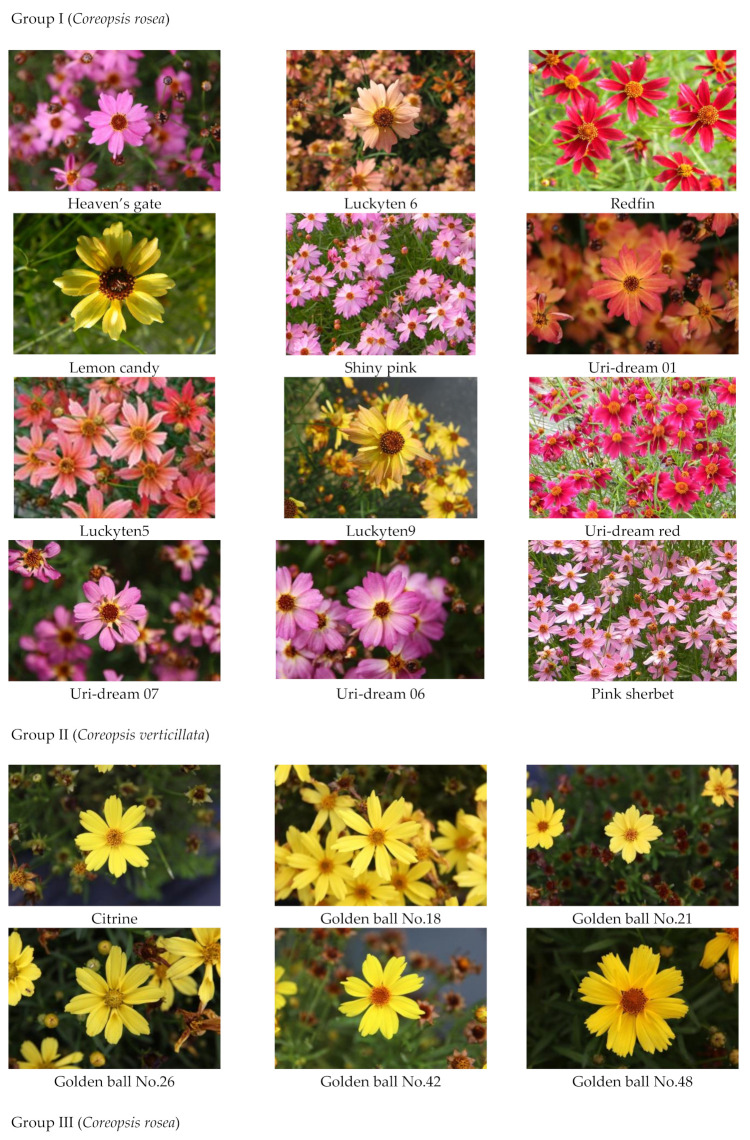

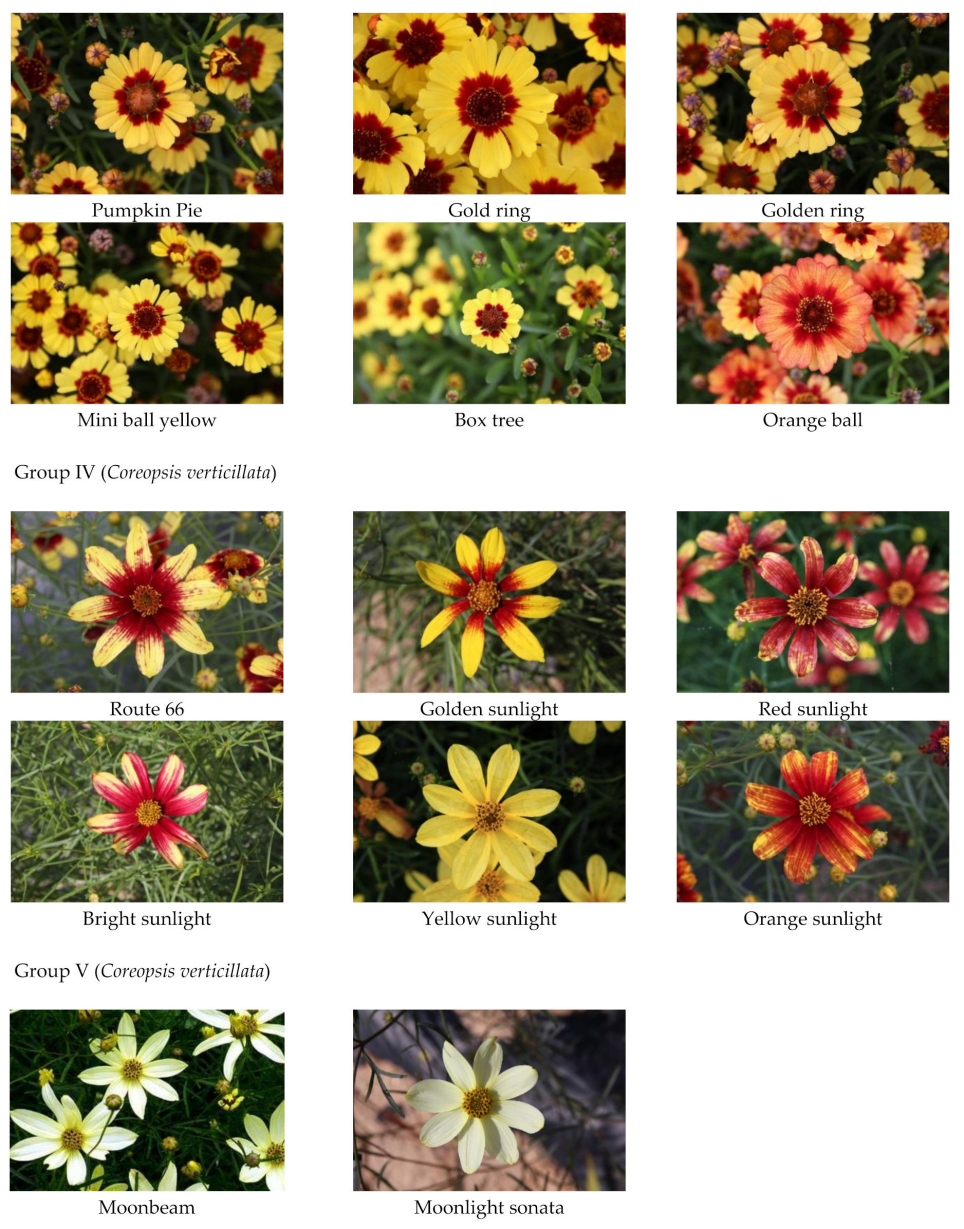
Photos of original and mutant cultivars of *Coreopsis rosea* and *Coreopsis verticillata* used in this study.

In this study, several new cultivars of *C. rosea* and *C. verticillata* throughout γ-irradiated mutation or herbicide-induced artificial mutation were developed and registered in the Korea Seed and Variety Service (Table 1 and Figure 1) [20]. For horticultural purposes and the improvement of quality and functionality, numerous cultivars have been developed by hybridization, and mutations were induced by chemical mutagens and ionizing radiation in plant breeding programs [21]. Previous studies on *C. rosea* and *C. verticillata* have primarily focused on plant growth impact assessment, new variety development, and horticulture [22,23,24,25]. However, there has been no report on the phytochemical and biological activity of *C. rosea* and *C. verticillata*, except for our previous study on the volatiles’ composition and antioxidant activity of *C. rosea* cultivars [18]. 

As part of our investigation of the effects of mutation on metabolic changes between the original mutant cultivars and their biological functions, we analyzed metabolite profiling of the five original cultivars and each mutant cultivar. Given that *Coreopsis* species have been known to be effective for diabetes in folk medicine, 70% methanol extracts of 32 *Coreopsis* samples were evaluated for their inhibitory effect against dipeptidyl peptidase (DPP)-IV, a target of incretin-based therapies for the treatment of type 2 diabetes mellitus.

## 2. Results

### 2.1. Subsection Identification of Metabolites in Coreopsis Cultivars Using UPLC-QTof-MS

Metabolites in *Coreopsis* cultivars were tentatively identified using UPLC-QTof-MS. The Metabolites were separated with high resolution within 10 min in the base peak ion (BPI) chromatogram. BPI chromatograms of the original *Coreopsis* cultivars are shown in Figure S1. The mass spectrum of each peak was carefully interpreted by analyzing its experimental and theoretical high resolution MS (the deprotonated molecular ion, [M – H]^−^), error ppm, molecular formula, and MS/MS fragmentation. Additionally, these were compared with data from the literature of plants belonging to the same genus, such as *C*. *tinctoria* (known as snow chrysanthemum) and *C*. *lanceolata* [3,5,6,12,26,27,28,29]. Moreover, its mass spectrum was compared to that in Waters Traditional Medicine Library that is built in UNIFI software (Waters, Milford, MA, USA) and MassBank available online (a public database for sharing mass spectral data) [30,31]. Forty compounds, including phenolic acids, flavonoids, and a polyacetylene were identified in methanol extracts of original and mutant cultivars of *C*. *rosea* and *C*. *verticillata* (Table 2). However, a peak observed in total ion chromatograms of all, or some *Coreopsis* cultivars could not be identified in this study.

#### 2.1.1. Phenolic Acids

Chlorogenic acid (peak 2, *t*_R_ 4.50 min) produced a major molecular ion at m/z 353.0864 [M – H]^−^ (calculated for C_16_H_17_O_9_^−^, 353.0878). At high energy scan, a fragment ion for a quinic acid was observed at m/z 191.0556 [3,26,27]. Peak 4 (*t*_R_ 5.21 min) produced a molecular ion at m/z 329.0865 [M – H]^−^ corresponding to C_14_H_17_O_9_^−^ and produced two fragment ions at m/z 167.0338 and 151.0026, assigned to the loss of glucose and [M – H – glucose – O]^−^, respectively. This peak was tentatively identified as vanillic acid-4-glucoside, which was confirmed by the UNIFI local library and first detected in *Coreopsis* species. Peak 27 (*t*_R_ 7.68 min) produced a major ion at m/z 515.1183 [M – H]^−^ (calculated for C_25_H_23_O_12_^−^, 515.1195) with two stable fragment ions at m/z 353.0869 [M – H – C_9_H_6_O_3_]^−^ and 191.0557 [M – H – 2C_9_H_6_O_3_]^−^ assigned to dicaffeoylquinic acid. Peak 31 (*t*_R_ 8.04 min) had the same precursor and fragment ions with peak 27. Peaks 27 and 31 were tentatively identified as 3,5-dicaffeoylquinic acid and 4,5-dicaffeoylquinic acid, respectively, by comparing with values in the literature of clearly identified constituents in *C*. *tinctoria* [26].

#### 2.1.2. Flavanones and Flavanonols

It has been reported that a retro Diels–Alder reaction, as well as the loss of H_2_O, sugar (usually glucose), and carbonyl groups were observed in the ion fragmentation pathways of flavonoids [3]. Flavanones, chalcones, and their glycosides have been known as the major types of flavonoids found in *Coreopsis* species [28]. These compounds usually showed the loss of H_2_O caused by the disposition of hydroxyls at C-3′ and C-4′ in the flavanone structure or at C-3 and C-4 in the chalcone structure, and the loss of a glucose at C-7 in the flavone structure or at C-4′ in the chalcone structure [3]. These phenomena were observed in mass spectra of flavanones and chalcones identified in this study. Abundant fragment ions, [M – H – glucose]^−^ and [M – H – glucose – H_2_O]^−^ for their glycosides and [M – H – H_2_O]^−^ for aglycones were produced by the loss of glucose and H_2_O, respectively. The loss of C_8_H_6_O (118 Da) or C_8_H_6_O_2_ (134 Da) were also characteristic fragment ions for flavanones and chalcones [3].

Peaks 1 (*t*_R_ 4.48 min) and 3 (*t*_R_ 4.96 min) produced a major molecular ion at m/z 465.1030 [M – H]^−^ (calculated for C_21_H_21_O_12_^−^, 465.1038) and yielded fragment ions at m/z 303.0503 [M – H – glucose]^−^ and 285.0397 [M – H – glucose – H_2_O]^−^ by the loss of glucose and H_2_O, indicating the presence of a 3′,4′-dihydroxyphenyl group. The fragment ion at m/z 151.0034 [M – H – glucose – H_2_O – C_8_H_6_O_2_]^−^ showed the presence of a 3-hydroxy group. Peak 3 had the fragment ion at 287.0550 [M – H – glucose – O]^−^, assuming that the sugar was attached at C-3 in the C ring. Therefore, peaks 1 and 3 were tentatively identified as taxifolin-7-*O*-glucoside [3] and taxifolin-3-*O*-glucoside [27], respectively. The aglycone of these compounds, taxifolin (peak 17, *t*_R_ 6.87 min), showed the same fragment ions with those of its glycosides (peaks 1 and 3) [3,26]. Peak 5 (*t*_R_ 5.74 min) produced a molecular ion at m/z 449.1085 [M – H]^−^, corresponding to the molecular formula C_21_H_21_O_11_^−^. At high energy scan, fragment ions at m/z 287.0554 [M – H – glucose]^−^ and 269.0446 [M – H – glucose – H_2_O]^−^ were detected by the loss of glucose and H_2_O and indicated the presence of a 3′,4′-dihydroxyphenyl group for the B ring. Fragment ions at m/z 151.0034 [M – H – glucose – H_2_O – C_8_H_6_O]^−^ suggested no hydroxyl group at C-3. Thus, it was tentatively identified as flavanomarein [26]. The fragmentation pattern of peak 6 (*t*_R_ 5.85 min) was identical to that of peak 5, except for the major molecular ion at m/z 595.1649 [M – H]^−^ (calculated for C_27_H_31_O_15_^−^, 595.1668) and the fragment ion at m/z 449.1069 [M – H – rhamnose]. Therefore, peak 6 was predicted as isookanin-7-*O*-rutinoside, which has been described previously [27]. Given that peak 16 (*t*_R_ 6.64 min) also exhibited the same fragment ions with those of peaks 5 and 6, it was tentatively identified as their aglycone, isookanin [3,26]. Peak 8 (*t*_R_ 5.98 min) produced a molecular ion at m/z 433.1135 [M – H]^−^ (calculated for C_21_H_21_O_10_^−^, 433.1140), which is 16 Da less than that of peak 5, and the fragment ion at m/z 135.0449 [M – H – glucose – H_2_O – C_8_H_6_O]^−^ indicated the presence of a 3′,4′-dihydroxyphenyl group and no hydroxyl group at C-3 in the C ring, and consequently confirmed the presence of a hydroxyl group in the A ring. Thus, peak 8 was tentatively identified as butin-7-*O*-glucoside [3]. Peak 9 (*t*_R_ 6.17 min) produced a molecular ion at m/z 479.0825 [M – H]^−^, corresponding to the molecular formula C_21_H_19_O_13_^−^. The fragment ions at m/z 317.0291 [M – H – glucose]^−^ and 166.9963 [M – H – glucose – CH_3_ – H_2_O – C_8_H_6_O]^−^ represented the presence of two hydroxy groups at C-3′ and C-4′ in the B ring and the absence of a hydroxy group at C-3 in the C ring, and consequently predicted the presence of 5,7-dihydroxy-8-methoxyphenyl group for the A ring. Therefore, peak 9 was tentatively identified as 8-methoxyeriodictyol-7-*O*-glucoside. Its aglycone, 8-methoxyeriodictyol, has been isolated from several plants [32,33,34]; however, its glycoside form has not been described previously. Peaks 10 (*t*_R_ 6.23 min) and 11 (*t*_R_ 6.34 min) produced the same molecular ion at m/z 463.1239 [M – H]^−^ (calculated for C_22_H_23_O_11_^−^, 463.1246) and the same fragment ion at m/z 165.0188 [M – H – glucose – H_2_O – C_8_H_6_O]^−^, indicating the presence of a 3′,4′-dihydroxyphenyl group for the B ring and no hydroxyl group at C-3 in the C ring, and consequently predicted the presence of the 7-hydroxy-8-methoxyphenyl group for the A ring. Therefore, peaks 10 and 11 were tentatively identified as coreolanceoline B [12] and lanceolin [6], respectively. Peak 12 (*t*_R_ 6.48 min) produced a molecular ion at m/z 433.1134 [M – H]^−^ (calculated for C_21_H_21_O_10_^−^, 433.1140), exhibiting the same molecular ions as peak 8; however, an [M – H – glucose – C_8_H_8_O]^−^ ion, instead of a [M – H – glucose – H_2_O]^−^ ion of peak 8, was observed in the fragmentation pattern of peak 12, indicating the presence of one hydroxy group in the B ring and two hydroxy groups in the A ring. Accordingly, peak 12 was tentatively identified as naringenin-7-*O*-glucoside [26]. Peak 14 (*t*_R_ 6.52 min) produced a molecular ion at m/z 595.1664 [M – H]^−^ (calculated for C_27_H_31_O_15_^−^, 595.1668) and the fragment ions at m/z 433.1121 [M – H – glucose]^−^ and 271.0604 [M – H – 2glucose]^−^, indicating the presence of two glucose groups. The fragment ion at m/z 135.0447 [M – H – 2glucose – C_8_H_8_O_2_]^−^ without the loss of H_2_O resulted in the presence of two hydroxy groups at C-3′ and C-5′ in the B ring. Thus, peak 14 was tentatively identified as 7,3′,5′-trihydroxyflavanone-*O*-diglucoside. Given that 7,3′,5′-trihydroxyflavanone-7-*O*-glucoside and its aglycone have been found in *Coreopsis* species [3,28], three types of this compound, 7,3′,5′-trihydroxyflavanone-7-*O*-(glucosyl glucoside), 7,3′,5′-trihydroxyflavanone-7,3′-*O*-di glucoside, and 7,3′,5′-trihydroxyflavanone-7,5′-*O*-diglucoside were predicted as possible structures; however, the three compounds have not been described previously. Peak 18 (*t*_R_ 6.91 min) produced a molecular ion at m/z 581.1501 [M – H]^−^ (calculated for C_26_H_29_O_15_^−^, 581.1512) and the fragment ion at m/z 287.0552 [M – H – arabinose – glucose] by a loss of the arabinosyl-glucose. The fragment ion at m/z 167.0342 [M – H – arabinosyl-glucose – C_8_H_6_O] without the loss of H_2_O indicated the presence of a hydroxy group in the B ring and three hydroxyl groups in the A ring. Thus, peak 18 was tentatively identified as 4′,5,7,8-tetrahydroxyflavanone-7-*O*-(6-*O*-arabinosyl-glucoside), which has not been described previously. Its aglycone, isocarthamidin (4′,5,7,8-tetrahydroxyflavanone), has not been reported in *Coreopsis* species; however, it has been isolated from the Asteraceae plant [35]. Peak 24 (*t*_R_ 7.26 min) produced a molecular ion at m/z 493.0984 [M – H]^−^ (calculated for C_22_H_21_O_13_^−^, 493.0988) and fragment ions at 331.0447 [M – H – glucose]^−^ and 316.0200 [M – H – glucose – CH_3_]^−^ by loss of a glucose and a methyl of the methoxy group, respectively. The fragment ion at m/z 164.9830 [M – H – glucose – CH_3_ – H_2_O – C_8_H_6_O_2_]^−^ produced a 3′-methoxy-4′-hydroxyphenyl group (or 3′-hydroxy-4′-methoxy phenyl group) for the B ring and a 3-hydroxy group in the C ring. Accordingly, peak 24 was tentatively identified as taxifolin 3′,7-dimethyl ether 3-*O*-glucoside [36]. Another candidate, taxifolin 4′,7-dimethyl ether 3-*O*-glucoside, has not been described previously; however, aglycones, taxifolin 4′,7-dimethyl ether, and taxifolin 3′,7-dimethyl ether have been reported in Asteraceae plants [37,38,39].

#### 2.1.3. Chalcones

Peak 23 has the same fragment rules as flavanomarein; however, it has been known that flavanones have shorter retention times than chalcones in chromatographic elution [29]. Therefore, peak 23 (*t*_R_ 7.15 min) was identified as marein [3,26,27]. The aglycone of this compound, okanin (peak 32, *t*_R_ 8.26 min), produced identical fragment ions with peak 23 [3,26,27]. Similarly, peak 30 (*t*_R_ 8.02 min) showed the same molecular ion and fragment ions as peak 8, thus identified as coreopsin [3,26]. Butein (peak 38, *t*_R_ 9.20 min), the aglycone of peak 30, showed a molecular ion at m/z 271.0605 [M – H]^−^ (calculated for C_15_H_11_O_5_^−^, 271.0612) and identical fragment ions with peak 30 [3,27]. Peak 13 (*t*_R_ 6.51 min) produced a molecular ion at m/z 611.1612 [M – H]^−^, corresponding to the molecular formula C_27_H_31_O_16_^−^. At high energy scan, the fragment ions at m/z 449.1080, 287.0551, and 269.0393 were formed by the loss of one glucose, two glucoses, and H_2_O, respectively, indicating the presence of two glucose groups and a 3,4-dihydroxyphenyl group for the B ring. Hence, peak 13 was tentatively identified as okanin-3′,4′-*O*-diglucoside, which has been isolated from *Bidens pilosa* [40]. Peak 33 (*t*_R_ 8.46 min) produced a molecular ion at m/z 611.1398 [M – H]^−^, corresponding to the molecular formula C_30_H_27_O_14_^−^. At high energy scan, fragment ions at m/z 449.1109 [M – H – glucose]^−^ and 287.0559 [M – H – 2glucose]^−^ were produced by the loss of two glucoses and 269.0441 [M – H – 2glucose – H_2_O]^−^ by the loss of H_2_O, and the fragment ion at m/z 151.0024 [M – H – 2glucose – H_2_O – C_8_H_6_O]^−^ indicated the presence of a 3′,4′-dihydroxyphenyl group for the B ring and the absence of a 3-hydroxy group in the C ring. Therefore, peak 33 was tentatively identified as eriodictyol chalcone-7-*O*-(glucosyl glucoside) or eriodictyol chalcone-*O*-diglucoside, which have not been described previously. Eriodictyol chalcone-7-*O* glucoside, which has one glucose, has been found in *Antirrhinum majus* [41]. Peak 34 (*t*_R_ 8.73 min) produced a molecular ion at m/z 287.0553 [M – H]^−^ and exhibited the same fragment pathway with that of peak 33, suggesting that it was an aglycone of peak 33, eriodictyol chalcone, which has been identified in *Coreopsis* species [42]. Peak 37 (*t*_R_ 8.84 min) produced a molecular ion at m/z 477.1396 [M – H]^−^, corresponding to the molecular formula C_23_H_25_O_11_^−^. At high energy scan, fragment ions were produced at m/z 315.0864 [M – H –glucose]^−^, 300.0624 [M – H –glucose – CH_3_]^−^, 297.0754 [M – H –glucose – H_2_O]^−^, 282.0527 [M – H – glucose – H_2_O – CH_3_]^−^, 163.0747 [M – H – glucose – H_2_O – C_8_H_6_O]^−^, and 148.00524 [M – H – glucose – H_2_O – CH_3_ – C_8_H_6_O]^−^. Therefore, peak 37 was tentatively identified as 4-methoxylanceoletin-4′-*O*-glucoside, which has been isolated from *C*. *lanceolata* [12], or lanceolein 2′-methyl ether, which has not been described previously.

#### 2.1.4. Flavones and Flavanols

Flavone having a double bond between C-2 and C-3 exhibits a molecular ion that is 2 Da less than that of flavanone or chalcone and characteristic fragment ions by the loss of C_8_H_4_O (116 Da) or C_8_H_4_O_2_ (132 Da) [3]. Peak 7 (*t*_R_ 5.93 min) produced a molecular ion at m/z 609.1454 [M – H]^−^ (calculated for C_27_H_29_O_16_^−^, 609.1461) and fragment ions at m/z 447.0932 [M – H – glucose]^−^ and 285.0392 [M – H – glucose – glucose]^−^ by the loss of two glucoses. Another fragment ion at m/z 151.0033 [M – H – 2glucose – H_2_O – C_8_H_4_O]^−^ indicated the presence of a 3′,4′-dihydroxyphenyl group for the B ring without a 3-hydroxy group in the C ring. Thus, peak 7 was tentatively identified as luteolin-7-*O*-sophoroside [3]. Peaks 22 (*t*_R_ 7.03 min) and 36 (*t*_R_ 8.80 min) produced molecular ions at m/z 447.0929 [M – H]^−^ (calculated for C_21_H_19_O_11_^−^, 447.0927) and m/z 285.0398 [M – H]^−^ (calculated for C_15_H_9_O_6_^−^, 285.0405) that were 162 Da and 324 Da less than that of peak 8, respectively, indicating that the sugar moiety was removed from C-2′′ and C-7 in luteolin-7-*O*-sophoroside (peak 7). Accordingly, peaks 22 and 36 were tentatively identified as luteolin-7-*O*-glucoside and luteolin, respectively [3,26,27]. In addition, a molecular ion at m/z 431.0978 [M – H]^−^ (calculated for C_21_H_19_O_10_^−^, 431.0984) for peak 29 (*t*_R_ 7.91 min) was 146 Da more than that of peak 36, indicating the addition of a rhamnose. Moreover, its fragment ions were similar to those of peaks 22 and 36. Thus, it was identified as luteolin-7-*O*-rhamnoside, which was first detected in *Coreopsis* species; however, it has been found in other plants, such as *Glechoma grandis* Kuprianova var. longituba, *Rumex algeriensis*, and *Cornulaca monacantha* [43,44,45]. Peak 15 (*t*_R_ 6.58 min) produced a molecular ion at m/z 609.1454 [M – H]^−^ (calculated for C_27_H_29_O_16_^−^, 609.1461) and fragment ions at m/z 447.0932 [M – H – glucose]^−^ and 285.0394 [M – H – 2glucose]^−^ by the loss of two glucoses. The fragment ion at m/z 151.0033 [M – H – 2glucose – H_2_O – C_8_H_4_O_2_]^−^ suggested the presence of a 3′,4′-dihydroxyphenyl group for the B ring and a 3-hydroxy group in the C ring. As a result, peak 15 was tentatively identified as fisetin-3,7-*O*-diglucoside, which was first detected in *Coreopsis* species; however, it has been found in other *Sophora* species [46]. Other glycosides of fisetin have not been previously described. Peak 20 (*t*_R_ 6.97 min) produced a molecular ion at 463.0885 [M – H]^−^, corresponding to the molecular formula C_21_H_19_O_12_^−^. At a high energy scan, the fragment ion at m/z 301.0346 [M – H – glucose]^−^ was observed by the loss of a glucose, and fragment ions at m/z 151.0034 [M – H – glucose – H_2_O – C_8_H_4_O_2_]^−^ indicated the presence of a 3′,4′-dihydroxyphenyl group by the loss of H_2_O and the presence of a 3-hydroxy group. Therefore, peak 20 was tentatively identified as quercetin-7-*O*-glucoside [3,26]. Peak 25 (*t*_R_ 7.33 min) showed a molecular ion at m/z 461.1085 [M – H]^−^ (calculated for C_22_H_21_O_11_^−^, 461.1089). At high energy scan, fragment ions at m/z 299.0547 [M – H – glucose]^−^, 283.0242 [M – H – glucose – O]^−^, 165.0188 [M – H – glucose – O – H_2_O – C_8_H_4_O]^−^, and 133.0291 [M – H – glucose – O – C_8_H_6_O_3_]^−^ were observed, indicating the loss of a glucoside at a 3-hydroxy group in the C ring and the presence of 3′,4′-dihydroxyphenyl group for the B ring. Therefore, peak 25 was tentatively identified as 3,3′,4′-trihydroxy-7-methoxyflavone 3-*O*-glucoside, which has been reported in *Aptenia cordifolia* [47]. Peak 26 (*t*_R_ 7.58 min) produced a molecular ion at m/z 641.1141 [M – H]^−^, corresponding to the molecular formula C_30_H_25_O_16_^−^. At high energy scan, fragment ions were produced at m/z 317.0294 [M – H – caffeoylglucose]^−^, 301.0342 [M – H – caffeoylglucose – O]^−^, 285.0381 [M – H – caffeoylglucose – O – O]^−^, 179.0343 [M – H – C_15_H_9_O_8_ – C_6_H_10_O_4_]^−^, 161.0224 [M – H – C_15_H_9_O_8_ – C_6_H_10_O_4_ – O]^−^, 135.0447 [M – H – C_15_H_9_O_8_ – C_6_H_10_O_4_ – CO_2_]^−^, and 133.0289 M – H – caffeoylglucose – O – O – C_7_H_3_O_4_]^−^. This peak was tentatively identified as qurcetagetin-7-O-(6′′-caffeoylglucoside), which was confirmed using the UNIFI local library and was first detected in *Coreopsis* species. However, this compound has been found in Asteraceae plants, such as *Gnaphalium uliginosum* and *Tagetes maxima* [48,49]. Peak 35 (*t*_R_ 8.74 min) produced a molecular ion at m/z 299.0555 [M – H]^−^ (calculated for C_16_H_11_O_6_^−^, 299.0561). The fragment ions produced at m/z 284.0319 [M – H – CH_3_]^−^ by the loss of a methyl of a methoxy group were observed. Moreover, 151.0032 [M – H – CH_3_ – C_8_H_5_O_2_]^−^ indicated the presence of a hydroxyl group at the B ring and a 3-hydroxy group in the C-ring. Other fragment ions at m/z 151.0032 [M – H – CH_3_ – C_8_H_6_O_2_]^−^ and 133.0447 [M – H – CH_3_ – C_7_H_3_O_4_]^−^ indicated the presence of a 4′-hydroxyphenyl group for the B ring with a 3-hydroxy group and two hydroxyl groups in the A ring, respectively. Thus, peak 35 was tentatively identified as kaempferide, by comparison of its mass spectrum with that in the MassBank database [31]. This compound was first detected in *Coreopsis* species; however, it has been found in Asteraceae plants, such as *Chrysanthemum morifolium*, *C*. *coronarium*, *Artemisia annua*, *Chromolaena odorata*, and *Filago germanica* [50,51,52]. Peak 39 (*t*_R_ 9.28 min) produced a molecular ion at m/z 269.0447 [M – H]^−^ (calculated for C_15_H_9_O_5_^−^, 269.0450) and fragment ions at m/z 227.0351 [M – H– C_2_H_2_O]^−^ and 117.0341 [M – H– C_7_H_4_O_4_]^−^. Thus, it was tentatively identified as apigenin [26].

#### 2.1.5. Aurones

Aurones are also one of the characteristic flavonoids found in *Coreopsis* species [3,12,29]. Peak 19 (*t*_R_ 6.94 min) produced a molecular ion at m/z 431.0977 [M – H]^−^ (calculated for C_21_H_19_O_10_^−^, 431.0984) and a fragment ion at m/z 269.0447 [M – H – glucose]^−^ by the loss of a glucose. In addition, fragment ions at m/z 135.0447 [M – H – glucose – H_2_O – C_8_H_4_O]^−^ and 133.0447 [M – H – glucose – C_7_H_4_O_3_]^−^ were also observed, indicating the presence of a 3′,4′-dihydroxyphenyl group for the B ring. Thus, peak 19 was tentatively identified as sulfurein (sulfuretin-6-*O*-glucoside) [35,53]. Sulfuretin (peak 28, *t*_R_ 7.77 min), the aglycone of peak 19, showed a molecular ion at m/z 269.0449 [M – H]^−^ (calculated for C_15_H_9_O_5_^−^, 269.0450) and identical fragment ions with peak 19 [27]. Peak 21 (*t*_R_ 7.01 min) showed a molecular ion at m/z 447.0929 [M – H]^−^ (calculated for C_21_H_19_O_11_^−^, 447.0927), which is 16 Da more than that of peak 20, and fragment ions at m/z 285.0397 [M – H – glucose]^−^, 135.0447 [M – H – glucose – H_2_O – C_8_H_4_O – O]^−^, and 133.0447 [M – H – glucose – C_7_H_4_O_3_]^−^, thus, identified as maritimein [3]. This identification was also confirmed by comparison with ESI-QTof-MS (negative ion mode) of maritimein in the MassBank database [31].

#### 2.1.6. Polyacetylene

Polyacetylenes of various structures have been isolated from the genus *Coreopsis* [12,28]. In this study, peak 41 (*t*_R_ 9.45 min) produced a molecular ion at m/z 557.2219 [M – H]^−^, a molecular formula of C_26_H_37_O_13_^−^. At high energy scan, fragment ions were produced at m/z 233.0650 [M – H – 2glucose]^−^, 191.0554 [M – H – 2glucose – C_3_H_6_]^−^, and 149.0441 [M – H – caffeoylglucose – C_5_H_8_O]^−^. This peak was tentatively identified as lobetyolinin, which was confirmed by the UNIFI local library and first detected in *Coreopsis* species; however, it has primarily been found in *Lobelia* species [54,55].

### 2.2. DPP-IV Inhibitory Effects of the 70% Ethanol Extract Obtained from Coreopsis cultivars

Type 2 diabetes mellitus is determined by several factors, including pancreas β-cell dysfunction, insulin resistance, increased hepatic and intestinal glucose production, or deficient insulin secretion [56]. Recently, the incretin effect has been observed to be reduced in patients with type 2 diabetes mellitus, which is a symptom of increased insulin secretion induced by oral administration, such as eating a meal, compared to intravenous administration of glucose [56]. This effect is mediated by incretin hormones, glucagon-like peptide-1 (GLP-1), and glucose-dependent insulinotropic polypeptide (GIP), which stimulate insulin secretion from pancreatic β-cells and consequently increase the blood glucose level [57,58]. In the incretin system, an increase of the elimination of GLP-1 and GIP occurs primarily through enzymatic degradation of DPP-IV [59]. Thus, DPP-IV inhibition enhances the function of insulinotropic hormones. It improves glucose tolerance in patients with type 2 diabetes mellitus [58]. Hence, DPP-IV inhibitors have emerged as a new class of oral anti-diabetic agents, and synthetic compounds have mainly been used in current treatments with these inhibitors [59]. However, there have also been studies that show that DPP-inhibitors are derived from natural sources as promising candidates of functional foods or pharmaceuticals [12,60,61,62,63].

In this study, the 70% ethanol extract of original and mutant cultivars of *C*. *rosea* and *C*. *verticillata* confirmed their anti-diabetic effect using an in vitro DPP-IV inhibitor screening assay. All extracts inhibited DPP-IV activity in a concentration-dependent manner with IC_50_ values from 34.01 to 134.28 μg/mL (Table 3). The positive control, sitagliptin, exhibited an IC_50_ of 0.095 μM. In two different species, the cultivars of *C*. *rosea* (Groups I and III) showed less inhibition of DPP-IV than the cultivars of *C*. *verticillata* (Groups II, IV, and V). Of the 32 samples, ‘Orange sunlight (No. 30)’, which belongs to Group IV (*C. verticillata* ), showed the greatest DPP-IV inhibitory effects. Thereafter, the most active cultivars with IC_50_ values less than 65 μg/mL were in the order of ‘Golden sunlight (No. 26)’, ‘Golden ball No.48 (No. 18)’, ‘Golden ball No.42 (No. 17)’, ‘Red sunlight (No. 27)’, ‘Bright sunlight (No. 28)’, and ‘Golden ball No.21 (No. 15)’, all of which belonged to *C*. *verticillata*. The DPP-IV inhibitory effects of six mutant cultivars, ‘Lemon candy (No. 4)’, ‘Shiny pink (No. 5)’, ‘Uri-dream 01 (No. 6)’, ‘Luckyten5 (No. 7)’, ‘Luckyten9 (No. 8)’, and ‘Uri-dream red (No. 9)’ were greater by 24–47 % than that of the original cultivar, ‘Heaven’s gate (No. 1)’ in Group I, while other mutant cultivars, ‘Luckyten 6 (No. 2)’, ‘Redfin (No. 3)’, ‘Uri-dream 07 (No. 10)’, ‘Uri-dream 06 (No. 11)’, and ‘Pink sherbet (No. 12)’ had similar or lower efficacy. In Group II, except for ‘Golden ball No.26 (N. 16)’, four mutant cultivars, ‘Golden ball No.18 (No. 14)’, ‘Golden ball No.21 (No. 15)’, ‘Golden ball No.42 (No. 17)’, and ‘Golden ball No.48 (No. 18)’ showed a 5–26 % increase in the inhibitory effect of DPP-IV compared to the original cultivar, ‘Citrine (No. 9)’. In Groups III and V, mutant cultivars exhibited similar or lower DPP-IV inhibitory effects than original cultivars. In Group IV, compared to the original cultivar, ‘Route 66 (No. 25)’, all mutant cultivars presented 7–37 % higher inhibitory effects of DPP-IV. 

Among all Coreopsis cultivars samples, ‘Orange sunlight (No. 30)’ showed the best efficacy with an IC_50_ value of 34.01 μg/mL; however, ‘Uri-dream red (No. 9)’ (IC_50_, 66.46 μg/mL) had the highest increase with 47% DPP-IV inhibitory activity compared to the original cultivar (IC_50_, 125.29 μg/mL). Therefore, ‘Orange sunlight (No. 30)’ had the potential to develop as a functional food, such as a tea ingredient or a food additive for the prevention or treatment of type 2 diabetes. The mutant cultivars with a greater increase in activity compared to the original cultivar, such as ‘Uri-dream red (No. 9)’ may be used for studies to identify metabolites changed by mutation using multivariate analysis, and for further research on genomic mutation mechanism.

### 2.3. Multivariate Analysis

Metabolite differences among original and mutant cultivars, *C*. *rosea* and *C*. *verticillate*, were examined based on the metabolite profiles analyzed by UPLC-QTof-MS. However, it was difficult to find differences among the samples in chromatograms. Therefore, PCA and OPLS-DA were used to provide an effective visualization for the classification and differentiation of a metabolome system.

**Figure 2:**
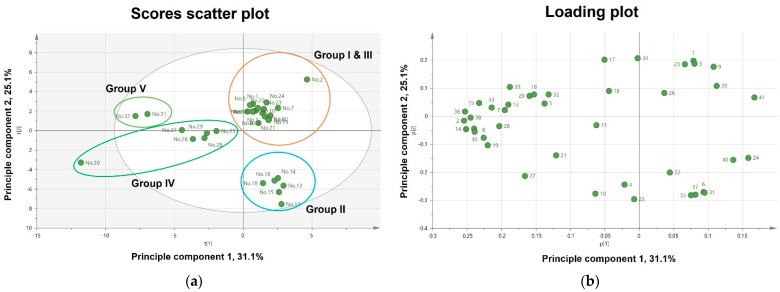

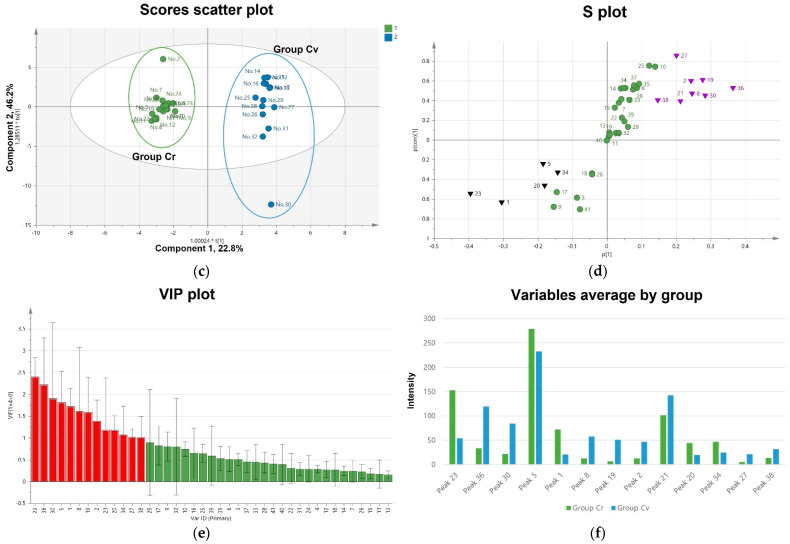
Principal component analysis (PCA) (**a**) score plot and (**b**) loading plot of metabolome analysis of the 32 *Coreopsis* cultivars; orthogonal partial least-squares discriminant analysis (OPLS-DA) (**c**) score plot and (**d**) S-plot show selected markers for differentiating *Coreopsis rosea* and *Coreopsis verticillata*; (**e**) Variable importance plot (VIP) scores of selected markers; (**f**) variables averages by group of selected potential marker compounds.

To compare metabolites from different cultivars of *C*. *rosea* and *C*. *verticillata*, we performed PCA analysis on negative ion mode data obtained from UPLC-QTof-MS analysis. PCA analysis was performed with three principal components (PC1–PC3) describing variation explained, 0.66 of *R^2^X* and predictive capability, 0.366 of *Q*^2^. Eigenvalues for PC1 and PC2 were found to be 9.94 and 8.05, respectively, indicating these first two principal components explain a large amount of the variance in the data. PC3 showed a comparatively smaller eigenvalue of 3.13, which led us to choose only PC1 and PC2 for further analysis. As shown in Figure 2A, the first two principal components described 56.2% of the total variation (31.1% and 25.1% by PC1 and PC2, respectively), and 32 *Coreopsis* samples were clearly clustered into four groups. Group I and Group III were clustered together, indicating similar chemical profiles among samples, and these two groups were of the same species, *C*. *rosea*. This cluster also suggested that there is no distinct difference between original cultivars and other mutation cultivars induced from each original one. However, an exception was found in ‘Luckyten 6 (No. 2)’, which is one of the mutant cultivars artificially induced using herbicide from the original cultivar of Group I, ‘Heaven’s gate (No. 1)’. Alternatively, a distinct separation was observed from cultivars in Group II, Group IV, and Group V, although they were all included in *C*. *verticallata*. Group II demonstrated different chemical profiles compared with Group IV and Group V. However, Group II showed one clustering with no substantial deviations between the γ-irradiated mutant cultivars (No.14–No. 18) and the original cultivar ‘Citrine (No. 13)’. In Group IV, the γ-irradiated mutant cultivar, ‘Orange sunlight (No. 30)’ was shown as the outlier, indicating that it had a different chemical profile than samples within the same group. Figure 2B, shows the derivation of markers primarily distributed among the four groups. However, this resulted in whole variability directions, with no distinction of variabilities among groups. Accordingly, we performed OPLS-DA analysis on the metabolite profiles between *C*. *rosea* cultivars (Group Cr) and *C*. *verticallata* cultivars (Group Cv) to find the differentiation and significant variances in these two species. Two clusters were clearly differentiated from each other according to species in the OPLS-DA model, with a cumulative R^2^Y value of 1.00 and a cumulative *Q*^2^ value of 0.94 (Figure 2C). However, ‘Luckyten 6 (No. 2)’ and ‘Orange sunlight (No. 30)’ were marginally out of each grouped sample area. The internal validation of OPLS-DA model was performed by a permutation test (*n* = 200). In permutation test, the intercept values of *R*^2^ and *Q*^2^ were 0.425 and −1.09 respectively. All permutations of the *R*^2^ and *Q*^2^ values to the left were lower than the original points to the right and the intersection of regression lines of the *R*^2^ and *Q*^2^ points on vertical axis was below 0.4 and −1.1, respectively (Figure S43). These values indicated OPLS-DA model of this analysis was strongly validated without overfitting of the original model. As shown in Figure 2D, the corresponding OPLS-DA S-plot enabled the derivation of 13 potential marker compounds responsible for separating two groups by being far from the center. Eight marker metabolites which were shifted in the same direction as Group Cv from the OPLS-DA score plot were peaks 2, 8, 19, 21, 27, 30, 36, and 38, indicating the most abundant markers in Group Cv. Five marker metabolites, peaks 1, 5, 20, 23, and 34, were at the highest level in Group Cr. The variable importance plot (VIP) (Figure 2E) confirms these 13 selected marker compounds are primarily responsible for the discrimination between Group Cr and Group Cv with high VIP values (VIP ≥ 1). Moreover, the variable average by group clearly shows differences of selected marker compounds (Figure 2F) in these groups. 

**Figure 3:**
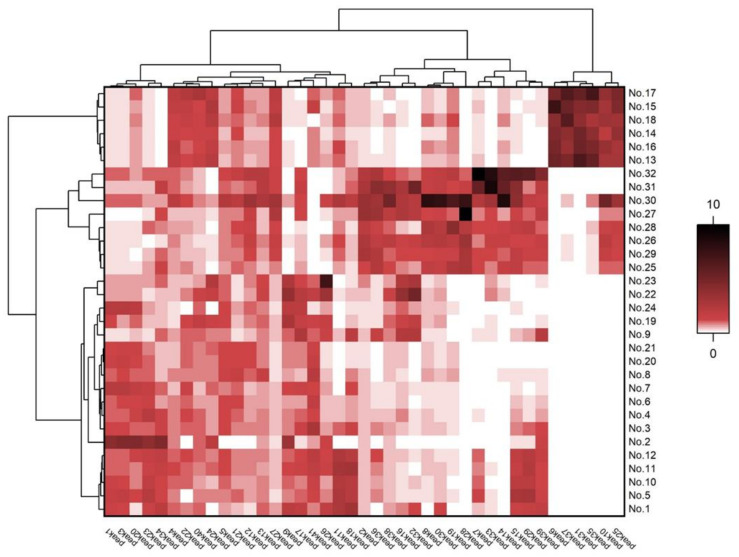
Hierarchical clustering analysis (HCA) with a heatmap from original and mutant cultivars of *Coreopsis* species.

The similarities in chemical composition and relative quantitative differences among different cultivars of *C*. *rosea* and *C*. *verticallata* were clearly visualized on a heatmap with a dendrogram, while a hierarchical cluster analysis exhibited the same pattern of clustering as observed in PCA analysis (Figure 3). Heatmap is considered as one of the best tools for converting qualitative data into quantitative. Group I (No. 1–No. 12) and Group III (No. 19–No. 24) were clustered as one big cluster with similar distribution of areas of peaks 1, 3, 4, 5, 9, 11, 12, 13, 17, 20, 21, 22, 23, 24, 26, 27, 34, 40, and 41. ‘Luckyten 6’ (No. 2) was observed to have comparatively higher area values for peaks 1, 3, 20, and 34 than other cultivars in Group I, indicating the relatively high contents of these four peaks when compared to other samples in Group I. These four peaks could be responsible for making ‘Luckyten 6’ (No. 2) an outlier. Peaks 6, 25, 31, 35, and 37 appear with intense color in heatmap representing high quantity in comparison with other samples, which was responsible for the clustering of group II (No. 13–No. 18). Group IV has peaks 2, 8, 10, 14, 19, 28, 30, and 36 in abundance, while ‘Orange sunlight’ (No. 30) is rich in peaks 8, 10, 14, 19, 28, and 30 among groups. These six peaks’ composition and relatively higher content could turn ‘Orange sunlight’ (No. 30) into an outlier in this statistical study. The contents of eight peaks 7, 14, 15, 29, 32, 33, 36, and 39 determine the clustering of group V (No. 31–No. 32), adjacent to group IV, sharing some similarities between them. 

The results of multivariate analyses to verify the correlation between metabolites and DPP-IV activities of the 32 *Coreopsis* samples were similar to the chemometric patterns between the two species. Given that DPP-IV inhibitory activities of *C*. *verticallata* cultivars appeared greater than that of *C*. *rosea* cultivars, distinguished metabolites between the active and inactive groups were almost identical to metabolites that showed differences between the two *Coreopsis* species presented in Table 3 (*C*. *verticallata*: IC_50_ < 65 µg/mL and *C*. *rosea*: IC_50_ > 65 µg/mL). Notably, ‘Orange sunlight (No. 30)’ and ‘Luckyten 6 (No. 2)’, which are outliers of Group Cv and Group Cr, respectively, were found to have the greatest and lowest DPP-IV inhibitory activity, respectively. These results suggested that the composition and relative content of distinguishable markers between *C*. *rosea* and *C*. *verticallata* cultivars were evaluated as key markers for the classification of species and contribution to the correlation of active and inactive cultivars.

## 3. Materials and Methods

### 3.1. Plant Material

*Coreopsis* cultivars were grown and collected from a wild cultivation field at Uriseed Group, Icheon-si, Gyeonggi-do, Republic of Korea and authenticated by Yeo Gyeong Jeon and Kong Young Park. These cultivars were selected according to their diverse phenotypic variants and exhibited a stable inheritance of these phenotypes for 4 years. Among them, five γ-irradiated mutants (Redfin, Lemone candy, Shiny pink, Uri-dream red, pink sherbet) of the original cultivar (Heaven’s gate) and the series of γ-irradiated mutants of original cultivars (Citirne, Pumpkin pie, Route 66) were generated using γ (^60^Co) irradiation (150 TBq capacity; AECL, Ottawa, ON, Canada). Six other mutant cultivars of ‘Heaven’s gate’ (Luckyten 6, Uri-dream 01, Luckyten5, Luckyten9, Uri-dream 07, Uri-dream 06) were artificially mutated using an herbicide. ‘Moonlight sonata’ was selected as the phenotypic variation of the original cultivar ‘Moonbeam’. Flowers used in this study were handpicked at the flowering stage in August 2018. These flowers were freeze-dried and stored at −20 °C for further analysis. Voucher specimens were deposited at the Uriseed Group Corporation.

### 3.2. Sample Preparation

Freeze-dried flowers of *Coreopsis* cultivars were ground into powder using a mixer. Extractions were performed with 200 mg of this powder in 20 mL of 70% methanol using an ultrasonic bath for 60 min, and subsequently evaporated to achieve a dry product. Thereafter, these dried extracts (1 mg each) were dissolved in 1 mL of 70% methanol and filtered through a 0.20 μm polyvinylidene fluoride filter. Samples (1000 ppm) were diluted with 70% methanol to a concentration of 200 ppm for further liquid chromatography–mass spectrometry (LC-MS) analysis. For the evaluation of bioactivity, methanol extracts were initially dissolved in dimethyl sulfoxide (DMSO) at a concentration of 10 mg/mL stock solution. All extraction and chromatographic solvents used in this study were of analytical grade (J. T. Baker, Phillipsburg, NJ, USA).

### 3.3. Ultra-Performance Liquid Chromatography Time-of-Flight Mass Spectrometry (UPLC-QTof-MS) Analysis

A Waters ACQUITY UPLC I-Class system equipped with a binary solvent pump and an autosampler combined with a Xevo G2-XS QTof-MS (Waters, Milford, MA, USA) was used. Each sample (1 μL) was injected into a ACQUITY UPLC BEH C_18_ column (2.1 mm i.d. × 100 mm, 1.7 μm) at a flow rate of 0.5 mL/min. The temperature of the column oven was maintained at 15 °C. The mobile phase was composed of 0.1% formic acid in water (*v*/*v*; solvent A) and 0.1% formic acid in acetonitrile (*v*/*v*; solvent B). Gradient elution was carried out as follows: 0–1.0 min, 1% B; 1.0–7.0 min, 1–20% B; 7.0–11.0 min, 20–40% B; 11.0–14.0 min, 60–100% B; 14.0–14.5 min, 100% B; 14.5–15.0 min, 100–1% B and 15.0–17.0 min, 1% B. The mass spectrometer was operated in negative ion mode with the following parameters: source temperature, 120 °C; desolvation temperature, 400 °C; capillary voltage, 3.0 kV; cone voltage, 40 V; cone gas flow: 50 L/h; flow rate of desolvation gas (N_2_), 1000 L/h; mass scan range, 550–1500 Da; scan time, 0.1 s. Leucine-enkephalin was used for the lock mass ([M ‒ H]^−^ m/z 554.2615). Full scan data, MS/MS spectra, accurate mass, and elemental composition were calculated using UNIFI software (Waters, Milford, MA, USA).

### 3.4. DPP-IV Inhibitor Screening Assay 

DPP-IV activity of *Coreopsis* cultivars was analyzed using a DPP-IV inhibitor screening assay kit (Cayman Chemical, Ann Arbor, MI, USA) which provides a fluorescence-based method for screening DPP-IV inhibitors. The assay uses the fluorogenic substrate, Gly-Pro-Aminomethylcoumarine (AMC), to measure DPP-IV activity. Cleavage of the peptide bond by DPP releases the free AMC group, resulting in fluorescence that can be analyzed using an excitation wavelength of 350–360 nm and an emission wavelength of 450–465 nm. The tested samples dissolved in DMSO at a concentration of 10 mg/mL were subsequently diluted to a final concentration of 20 to 200 μg/mL using DMSO and were added to a 96-well plate to a final volume of 10 μL and a final concentration of 50 μM. The assay procedure is described in our previous studies [12,62,63]. Briefly, diluted assay buffer (30 μL) and diluted enzyme solution (10 μL) were added to the 96-well plate containing 10 μL of solvent (blank) or solvent-dissolved test samples. The reaction was initiated by adding 50 μL of a diluted substrate solution, and the plate was incubated for 30 min at 37 °C. Following incubation, fluorescence with an excitation wavelength of 350 nm and an emission wavelength of 450 nm was monitored using a plate reader (TECAN, Männedorf, Switzerland). The percent inhibition was expressed as ([DPP-IV level of vehicle-treated control − DPP-IV level of test samples]/DPP-IV level of vehicle-treated control) × 100. Subsequently, the 50% inhibitory concentration (IC_50_) was determined using GraphPad Prism software (GaraphPad Software, La Jolla, CA, USA) via dose–response analysis.

### 3.5. Chemometric Data Analysis

Data management for the UPLC-QTof-MS analysis was performed using UNIFI software (Waters, Milford, MA, USA). MS data were processed using UNIFI to obtain a data matrix containing retention times, accurate masses, and normalized peak intensities. Parameters included retention time (*t*_R_, range of 0.0–15.0 min), mass-to-charge ratio (m/z, range of 100–1500 Da), and a mass tolerance of 0.04 Da. The resulting data were evaluated using SIMCA 15.0.2 (Umetrics, Umeå, Sweden) for multivariate statistical analysis. Unsupervised principal component analysis (PCA) was performed using UV (univariate)-scaled and supervised orthogonal partial least-squares discriminant analysis (OPLS-DA) was used to identify and compare different metabolite sizes of the 32 samples. The quality of the OPLS-DA model was evaluated with *R*^2^*Y* value and cumulative *Q*^2^ value. The model was further validated with a permutation test (*n* = 200). Markers for the difference between groups were identified by analyzing the S-plot with pareto scaling, which were generated with covariance (p) and correlation (pcorr) data. These data sets were normalized by dividing with mean value to get a value between 0 and 10 and a heatmap with dendrograms was generated using OriginPro 2021 (OriginLab Corporation, Northampton, MA, USA) selecting ward for cluster method. Marker compounds were tentatively identified by comparison to published MS data in literature and databases such as Waters Local Library in UNIFI and Massbank [3,5,6,26,27,28,29,30,31].

## 4. Conclusions

To the best of our knowledge, a comparative metabolomics approach to identify metabolite composition and DPP-IV inhibitory activities in various cultivars of *C. rosea* and *C. verticillata*, were demonstrated for the first time in this study. UPLC-QTof-MS techniques were used to identify several phenolic acids, flavonoids and a polyacetylene in mutant cultivars compared to original cultivars. PCA and OPLS-DA results showed that metabolites discriminate between the mutant and original cultivars and between the two species. In addition, significant changes in metabolite content were observed under different DPP-IV inhibitory activities of cultivars, and chlorogenic acid, butin-7-*O*-glucoside, sulfuretin-6-*O*-glucoside, maritimein, 3,5-dicaffeoylquinic acid, coreopsin, luteolin, and butein were abundant in the active extracts. Therefore, the DPP-IV inhibitory cultivars and the metabolites influencing their activities would be favorable for the development of functional foods and the information of the metabolites accumulated differently for each mutant cultivar would be useful as a scientific reference for further studies on plant mutation mechanisms.

## Figures and Tables

**Table 1:** The list of the original and mutant cultivars of *Coreopsis rosea* and *Coreopsis verticillata* used in this study.

Group (Plant Name)	No.	Cultivar Names	Registration No.	Application No.	Breeding Process
I (*C. rosea*)	1	Heaven’s gate	-	-	Original cultivar
	2	Luckyten 6	3869	-	Herbicide-induced artificial mutation
	3	Redfin	4408	-	γ-Irradiated mutation
	4	Lemon candy	4418	-	γ-Irradiated mutation
	5	Shiny pink	4420	-	γ-Irradiated mutation
	6	Uri-dream 01	3993	-	Herbicide-induced artificial mutation
	7	Luckyten5	4411	-	Herbicide-induced artificial mutation
	8	Luckyten9	4413	-	Herbicide-induced artificial mutation
	9	Uri-dream red	6001	-	γ-Irradiated mutation
	10	Uri-dream 07	3998	-	Herbicide-induced artificial mutation
	11	Uri-dream 06	3997	-	Herbicide-induced artificial mutation
	12	Pink sherbet	4415	-	γ-Irradiated mutation
II (*C. verticillata*)	13	Citrine	-	-	Original cultivar
	14	Golden ball No.18	6421	-	γ-Irradiated mutation
	15	Golden ball No.21	6422	-	γ-Irradiated mutation
	16	Golden ball No.26	5995	-	γ-Irradiated mutation
	17	Golden ball No.42	5997	-	γ-Irradiated mutation
	18	Golden ball No.48	5999	-	γ-Irradiated mutation
III (*C. rosea*)	19	Pumpkin Pie	-	-	Original cultivar
	20	Gold ring	7523	-	γ-Irradiated mutation
	21	Golden ring	5994	-	γ-Irradiated mutation
	22	Mini ball yellow	6453	-	γ-Irradiated mutation
	23	Box tree	6462	-	γ-Irradiated mutation
	24	Orange ball	6005	-	γ-Irradiated mutation
IV (*C. verticillata*)	25	Route 66	-	-	Original cultivar
	26	Golden sunlight	-	2018-406	γ-Irradiated mutation
	27	Red sunlight	-	2018-410	γ-Irradiated mutation
	28	Bright sunlight	-	2018-408	γ-Irradiated mutation
	29	Yellow sunlight	-	2018-411	γ-Irradiated mutation
	30	Orange sunlight	-	2018-399	γ-Irradiated mutation
V (*C. verticillata*)	31	Moonbeam	-	-	Original cultivar
	32	Moonlight sonata	-	2018-401	Selection of phenotypic variant

**Table 2:** Characterization and tentative identification of metabolites found in original and mutant cultivars of *Coreopsis rosea* and *Coreopsis verticillata* using ultra-performance liquid chromatography time-of-flight mass spectrometry (UPLC-QTof MS).

Peak No.	ESI-MS *t*_R_ (min)	Observed Mass (m/z)	Caculated Mass (m/z)	Error (ppm)	Molecular Formula	Key MS^E^ Fragment Ions (m/z)	Identification
1	4.48	465.1030	465.1039	−0.8	C_21_H_22_O_12_	303.0503, 285.0397, 151.0034, 125.0239	Taxifolin-7-*O*-glucoside
2	4.50	353.0864	353.0878	−1.4	C_16_H_18_O_9_	191.0556, 133.0290	Chlorogenic acid
3	4.96	465.1030	465.1039	−0.8	C_21_H_22_O_12_	303. 0503, 287.0550, 285.0397, 151.0034, 125.0234	Taxifolin-3-*O*-glucoside
4	5.21	329.0865	329.0878	−1.3	C_14_H_18_O_9_	167.0338, 151.0026	Vanillic acid-4-glucoside
5	5.74	449.1085	449.1089	−0.4	C_21_H_22_O_11_	287.0554, 269.0446, 151.0034, 135.0449	Flavanomarein
6	5.85	595.1649	595.1668	−1.9	C_27_H_32_O_15_	449.1069, 287.0548, 269.0428, 151.0028, 135.0447	Isookanin-7-*O*-rutinoside
7	5.93	609.1454	609.1461	−0.7	C_27_H_30_O_16_	447.0932, 285.0392, 151.0033	Luteolin-7-*O*-sophoroside
8	5.98	433.1135	433.1140	−0.5	C_21_H_22_O_10_	271.0605, 253.0499, 135.0449	Butin-7-*O*-glucoside
9	6.17	479.0825	479.0831	−0.6	C_21_H_20_O_13_	317.0291, 166.9963	8-Methoxyeriodictyol-7-*O*-glucoside
10	6.23	463.1239	463.1246	−0.7	C_22_H_24_O_13_	301.0708, 165.0188, 135.0449	Coreolanceoline B
11	6.34	463.1251	463.1246	−0.5	C_22_H_24_O_11_	301.0708, 165.0188, 135.0449	Lanceolin
12	6.48	433.1134	433.1140	−0.6	C_21_H_22_O_10_	271.0602, 151.0029, 119.0488	Naringenin-7-*O*-glucoside
13	6.51	611.1612	611.1618	−0.6	C_27_H_32_O_16_	449.1080, 287.0551, 269.0393, 135.0447	Okanin-3′,4′-*O*-diglucoside
14	6.52	595.1664	595.1668	−0.4	C_27_H_32_O_15_	433.1121, 271.0604, 135.0447	4′,7,8-Trihydroxyflavone-*O*-diglucoside
15	6.58	609.1454	609.1461	−0.7	C_27_H_30_O_16_	447.0932, 285.0394, 135.0082	Fisetin-3,7-*O*-diglucoside
16	6.64	287.0555	287.0561	−0.6	C_15_H_12_O_6_	151.0031, 135.0449	Isookanin
17	6.87	303.0502	303.0510	−0.8	C_15_H_12_O_7_	285.0399, 151.0084, 135.0447, 125.0240	Taxifolin
18	6.91	581.1501	581.1512	−1.1	C_26_H_30_O_15_	287.0552, 167.0342, 151.0029	4′,5,7,8-Tetrahydroxyflavanone-7-*O*-(6-*O*-arabinosyl-glucoside)
19	6.94	431.0977	431.0984	−0.7	C_21_H_20_O_10_	269.0447, 135.0447, 133.0290	Sulfuretin-6-*O*-glucoside
20	6.97	463.0885	463.0882	0.3	C_21_H_20_O_12_	301.0346, 151.0031	Quercetin-7-*O*-glucoside
21	7.01	447.0929	447.0927	0.2	C_21_H_20_O_11_	285.0397, 135.0447, 133.0291	Maritimein
22	7.03	447.0929	447.0927	0.2	C_21_H_20_O_11_	285.0397, 151.0033	Luteolin-7-*O*-glucoside
23	7.15	449.1081	449.1089	−0.8	C_21_H_22_O_11_	287.0551, 269.0445, 151.0033, 135.0448	Marein
24	7.26	493.0984	493.0988	−0.4	C_22_H_22_O_13_	331.0447, 316.0200, 164.9830	Taxifolin 3′,7-dimethyl ether 3-*O*-glucoside
25	7.33	461.1085	461.1089	−0.4	C_22_H_22_O_11_	299.0547, 283.0242, 165.0188, 133.0291	3,3′,4′-Trihydroxy-7-methoxyflavone 3-*O*-glucoside
26	7.58	641.1141	641.1148	−0.7	C_30_H_26_O_16_	317.0294, 301.0342, 285.0381, 179.0343, 161.0224, 135.0447, 133.0289	Qurcetagetin-7-*O*-(6′′-caffeoylglucoside)
27	7.68	515.1183	515.1195	−1.2	C_25_H_24_O_12_	353.0869, 191.0557, 179.0346, 135.0447	3,5-Dicaffeoylquinic acid
28	7.77	269.0449	269.0450	−0.1	C_15_H_10_O_5_	135.0447, 133.0287	Sulfuretin
29	7.91	431.0978	431.0984	−0.6	C_21_H_20_O_10_	285.0398, 151.0031, 133.0289	Luteolin-6-*O*-rhamnoside
30	8.02	433.1135	433.1140	−0.5	C_21_H_22_O_10_	271.0606, 135.0448	Coreopsin
31	8.04	515.1187	515.1195	−0.8	C_25_H_24_O_12_	353.0862, 191.0556, 179.0340	4,5-Dicaffeoylquinic acid
32	8.26	287.0556	287.0561	−0.5	C_15_H_12_O_6_	151.0032, 134.0368, 123.0083	Okanin
33	8.46	611.1398	611.1406	−0.8	C_30_H_28_O_14_	449.1109, 287.0559, 269.0441, 151.0024	Eriodictyol chalcone-*O*-diglucoside
34	8.73	287.0553	287.0561	−0.8	C_15_H_12_O_6_	151.0032, 135.0047	Eriodictyol chalcone
35	8.74	299.0555	299.0561	−0.6	C_16_H_12_O_6_	284.0319, 151.0032, 133.0291	Kaempferide
36	8.80	285.0398	285.0405	−0.7	C_15_H_10_O_6_	151.0032, 133.0291	Luteolin
37	8.84	477.1396	477.1402	−0.6	C_23_H_26_O_11_	315.0864, 300.0624, 282.0527, 148.0524, 135.0435	4-Methoxylanceoletin-4′-*O*-glucoside
38	9.20	271.0605	271.0612	−0.7	C_15_H_12_O_5_	253.0496, 135.0448,	Butein
39	9.28	269.0447	269.0450	−0.3	C_15_H_10_O_5_	227.0351, 117.0341	Apigenin
40	9.39	831.3595	831.3597	−0.2	C_46_H_56_O_14_	785.3536, 666.2998, 545.2401, 145.0291	Unknown
41	9.45	557.2244	557.2240	−2.1	C_26_H_38_O_13_	233.0650, 191.0554, 149.0441	Lobetyolinin

**Table 3:** Effects of the 70% ethanol extract of *Coreopsis* cultivars on dipeptidyl peptidase (DPP)-IV activity.

Group (Plant Name)	No.	Cultivar Names	DPP-IV Inhibitory Effects (IC_50_, μg/mL) ^1^
I (*C. rosea*)	1	Heaven’s gate	125.29
	2	Luckyten 6	158.83
	3	Redfin	117.55
	4	Lemon candy	95.39
	5	Shiny pink	76.92
	6	Uri-dream 01	95.53
	7	Luckyten5	78.06
	8	Luckyten9	78.60
	9	Uri-dream red	66.46
	10	Uri-dream 07	118.13
	11	Uri-dream 06	134.28
	12	Pink sherbet	117.70
II (*C. verticillata*)	13	Citrine	56.86
	14	Golden ball No.18	53.55
	15	Golden ball No.21	49.64
	16	Golden ball No.26	63.84
	17	Golden ball No.42	45.01
	18	Golden ball No.48	41.44
III (*C. rosea*)	19	Pumpkin Pie	76.40
	20	Gold ring	87.62
	21	Golden ring	89.22
	22	Mini ball yellow	74.57
	23	Box tree	76.83
	24	Orange ball	124.88
IV (*C. verticillata*)	25	Route 66	54.87
	26	Golden sunlight	40.37
	27	Red sunlight	45.42
	28	Bright sunlight	47.58
	29	Yellow sunlight	50.45
	30	Orange sunlight	34.01
V (*C. verticillata*)	31	Moonbeam	60.61
	32	Moonlight sonata	61.15
		Sitagliptin^2^	0.095 (μM)

^1^ Values are presented as the mean ± SD of three independent experiments. ^2^ Sitagliptin was used as the positive control.

## Supplementary Materials

The following are available online at https://www.mdpi.com/article/10.3390/plants10081661/s1, Figure S1: title, Table S1: title, Video S1: title. Figure S1: Representative UPLC-QTof-MS chromatograms of the 70% methanol extracts of the original cultivars at low CE scan (6 eV) for precursor (up) and high CE scan (20-50 eV) for fragment ions (down). (a) ‘Heaven’s gate’ (No. 1), (b) ‘Citrine’ (No. 13), (c) ‘Pumpkin Pie’ (No. 19), (d) ‘Route 66’ (No. 25), and (e) ‘Moonbeam’ (No. 31), Figure S2: ESI-QTof-MS spectrum of taxifolin-7-*O*-glucoside (peak 1), Figure S3: ESI-QTof-MS spectrum of chlorogenic acid (peak 2), Figure S4: ESI-QTof-MS spectrum of taxifolin-3-*O*-glucoside (peak 3), Figure S5: ESI-QTof-MS spectrum of vanillic acid-4-glucoside (peak 4), Figure S6: ESI-QTof-MS spectrum of flavanomarein (peak 5), Figure S7: ESI-QTof-MS spectrum of isookanin-7-*O*-rutinoside (peak 6), Figure S8: ESI-QTof-MS spectrum of luteolin-7-*O*-sophoroside (peak 7), Figure S9: ESI-QTof-MS spectrum of butin-7-*O*-glucoside (peak 8), Figure S10: ESI-QTof-MS spectrum of 8-methoxyeriodictyol-7-*O*-glucoside (peak 9), Figure S11: ESI-QTof-MS spectrum of coreolanceoline B (peak 10), Figure S12: ESI-QTof-MS spectrum of lanceolin (peak 11), Figure S13: ESI-QTof-MS spectrum of naringenin-7-*O*-glucoside (peak 12), Figure S14: ESI-QTof-MS spectrum of okanin-3′,4′-*O*-diglucoside (peak 13), Figure S15: ESI-QTof-MS spectrum of 4′,7,8-trihydroxyflavone-*O*-diglucoside (peak 14), Figure S16: ESI-QTof-MS spectrum of fisetin-3,7-*O*-diglucoside (peak 15), Figure S17: ESI-QTof-MS spectrum of isookanin (peak 16), Figure S18: ESI-QTof-MS spectrum of taxifolin (peak 17), Figure S19: ESI-QTof-MS spectrum of 4′,5,7,8-tetrahydroxyflavanone-7-*O*-(6-*O*-arabinosyl-glucoside) (peak 18), Figure S20: ESI-QTof-MS spectrum of sulfuretin-6-*O*-glucoside (peak 19), Figure S21: ESI-QTof-MS spectrum of quercetin-7-*O*-glucoside (peak 20), Figure S22: ESI-QTof-MS spectrum of maritimein (peak 21), Figure S23: ESI-QTof-MS spectrum of luteolin-7-O-glucoside (peak 22), Figure S24: ESI-QTof-MS spectrum of marein (peak 23), Figure S25: ESI-QTof-MS spectrum of taxifolin 3′,7-dimethyl ether 3-*O*-glucoside (peak 24), Figure S26: ESI-QTof-MS spectrum of 3,3′,4′-trihydroxy-7-methoxyflavone 3-*O*-glucoside (peak 25), Figure S27: ESI-QTof-MS spectrum of qurcetagetin-7-*O*-(6′′-caffeoylglucoside) (peak 26), Figure S28: ESI-QTof-MS spectrum of 3,5-dicaffeoylquinic acid (peak 27), Figure S29: ESI-QTof-MS spectrum of sulfuretin (peak 28), Figure S30: ESI-QTof-MS spectrum of luteolin-6-*O*-rhamnoside (peak 29), Figure S31: ESI-QTof-MS spectrum of coreopsin (peak 30), Figure S32: ESI-QTof-MS spectrum of 4,5-dicaffeoylquinic acid (peak 31), Figure S33: ESI-QTof-MS spectrum of okanin (peak 32), Figure S34: ESI-QTof-MS spectrum of eriodictyol chalcone-*O*-diglucoside (peak 33), Figure S35: ESI-QTof-MS spectrum of eriodictyol chalcone (peak 34), Figure S36: ESI-QTof-MS spectrum of kaempferide (peak 35), Figure S37: ESI-QTof-MS spectrum of luteolin (peak 36), Figure S38: ESI-QTof-MS spectrum of 4-methoxylanceoletin-4′-*O*-glucoside (peak 37), Figure S39: ESI-QTof-MS spectrum of butein (peak 38), Figure S40: ESI-QTof-MS spectrum of apigenin (peak 39), Figure S41: ESI-QTof-MS spectrum of unknown (peak 40), Figure S42: ESI-QTof-MS spectrum of lobetyolinin (peak 41), Figure S43: Validation plot of the OPLS-DA obtained from 200 permutation test.
