# Supplementary material for: Metabolite Profiling and Dipeptidyl Peptidase IV Inhibitory Activity of Coreopsis Cultivars in Different Mutations
Source: Plants (Basel). 2021 Aug 12;10(8):1661. doi: 10.3390/plants10081661 (PMC8401970; doi:10.3390/plants10081661)

Supporting Information

Metabolite Profiling and Dipeptidyl Peptidase IV Inhibitory Activity of Coreopsis Cultivars in Different Mutation

Bo-Ram Kim [1,2,†], Sunil Babu Paudel [3,†], Ah-Reum Han [1,†], Ji-Su Park [1], Yun-Seo Kil [3], Hyukjae Choi [3,4], Yeo Gyeong Jeon [5], Kong Young Park [5], Si-Yong Kang [6], Chang Hyun Jin [1], Jin-Baek Kim [1], and Joo-Won Nam [3,*]

1 Advanced Radiation Technology Institute, Korea Atomic Energy Research Institute (KAERI), Jeongeup-si, Jeollabuk-do 56212, Republic of Korea
2 Natural Product Research Division, Honam National Institute of Biological Resources, Mokpo-si, Jeollanam-do 58762, Republic of Korea
3 College of Pharmacy, Yeungnam University, Gyeongsan-si, Gyeongsangbuk-do 38541, Republic of Korea
4 Research Institute of Cell Culture, Yeungnam University, Gyeongsan, Gyeongbuk 38541, Republic of Korea
5 Uriseed Group, Icheon-si, Gyeonggi-do 17408, Republic of Korea
6 Department of Horticulture, College of Industrial Sciences, Kongju National University, Yesan-gun, Chungcheongnam-do 32439, Republic of Korea
* Correspondence: jwnam@yu.ac.kr (J.-W.N.); Tel. +53-810-2818 (J.-W.N.)
† These authors contributed equally to this work.

CONTENS

Figure S1. Representative UPLC-QTof-MS chromatograms of the 70% methanol extracts of the original cultivars at low CE scan (6 eV) for precursor (up) and high CE scan (20-50 eV) for fragment ions (down). (**a**) ‘Heaven’s gate’ (No. 1), (**b**) ‘Citrine’ (No. 13), (**c**) ‘Pumpkin Pie’ (No. 19), (**d**) ‘Route 66’ (No. 25), and (**e**) ‘Moonbeam’ (No. 31).

Figure S2. ESI-QTof-MS spectrum of taxifolin-7-O-glucoside (peak 1)

Figure S3. ESI-QTof-MS spectrum of chlorogenic acid (peak 2)

Figure S4. ESI-QTof-MS spectrum of taxifolin-3-O-glucoside (peak 3)

Figure S5. ESI-QTof-MS spectrum of vanillic acid-4-glucoside (peak 4)

Figure S6. ESI-QTof-MS spectrum of flavomarein (peak 5)

Figure S7. ESI-QTof-MS spectrum of isookanin-7-O-rutinoside (peak 6)

Figure S8. ESI-QTof-MS spectrum of luteolin-7-O-sophoroside (peak 7)

Figure S9. ESI-QTof-MS spectrum of butin-7-O-glucoside (peak 8)

Figure S10. ESI-QTof-MS spectrum of 8-methoxyeriodictyol-7-O-glucoside (peak 9)

Figure S11. ESI-QTof-MS spectrum of coreolanceoline B (peak 10)

Figure S12. ESI-QTof-MS spectrum of lanceolin (peak 11)

Figure S13. ESI-QTof-MS spectrum of naringenin-7-O-glucoside (peak 12)

Figure S14. ESI-QTof-MS spectrum of okanin-3,4′-O-diglucoside (peak 13)

Figure S15. ESI-QTof-MS spectrum of 4′,7,8-trihydroxyflavone-O-diglucoside (peak 14)

Figure S16. ESI-QTof-MS spectrum of fisetin-3,7-O-diglucoside (peak 15)

Figure S17. ESI-QTof-MS spectrum of isookanin (peak 16)

Figure S18. ESI-QTof-MS spectrum of taxifolin (peak 17)

Figure S19. ESI-QTof-MS spectrum of 4′,5,7,8-tetrahydroxyflavanone-7-O-(6-O-arabinosyl-glucoside) (peak 18)

Figure S20. ESI-QTof-MS spectrum of sulfuretin-6-O-glucoside (peak 19)

Figure S21. ESI-QTof-MS spectrum of quercetin-7-O-glucoside (peak 20)

Figure S22. ESI-QTof-MS spectrum of maritimein (peak 21)

Figure S23. ESI-QTof-MS spectrum of luteolin-7-O-glucoside (peak 22)

Figure S24. ESI-QTof-MS spectrum of marein (peak23)

Figure S25. ESI-QTof-MS spectrum of taxifolin 3′,7-dimethyl ether 3-O-glucoside (peak 24)

Figure S26. ESI-QTof-MS spectrum of 3,3′,4′-trihydroxy-7-methoxyflavone 3-O-glucoside (peak 25)

Figure S27. ESI-QTof-MS spectrum of qurcetagetin-7-O-(6′′-caffeoylglucoside) (peak 26)

Figure S28. ESI-QTof-MS spectrum of 3,5-dicaffeoylquinic acid (peak 27)

Figure S29. ESI-QTof-MS spectrum of sulfuretin (peak 28)

Figure S30. ESI-QTof-MS spectrum of luteolin-6-O-rhamnoside (peak 29)

Figure S31. ESI-QTof-MS spectrum of coreopsin (peak 30)

Figure S32. ESI-QTof-MS spectrum of 4,5-dicaffeoylquinic acid (peak 31)
Figure S33. ESI-QTof-MS spectrum of okanin (peak 32)
Figure S34. ESI-QTof-MS spectrum of eriodictyol chalcone-O-diglucoside (peak 33)
Figure S35. ESI-QTof-MS spectrum of eriodictyol chalcone (peak 34)
Figure S36. ESI-QTof-MS spectrum of kaempferide (peak 35)
Figure S37. ESI-QTof-MS spectrum of luteolin (peak 36)
Figure S38. ESI-QTof-MS spectrum of 4-methoxylanceoletin-4′-O-glucoside (peak 37)
Figure S39. ESI-QTof-MS spectrum of butein (peak 38)
Figure S40. ESI-QTof-MS spectrum of apigenin (peak 39)
Figure S41. ESI-QTof-MS spectrum of unknown (peak 40)
Figure S42. ESI-QTof-MS spectrum of lobetyolinin (peak 41)
Figure S43. Validation plot of the OPLS-DA obtained from 200 permutation test.

Figure S1. Representative UPLC-QTof MS chromatograms of the 70% methanol extracts of the original cultivars at low CE scan (6 eV) for precursor (up) and high CE scan (20-50 eV) for fragment ions (down). (**a**) ‘Heaven’s gate’ (No. 1), (**b**) ‘Citrine’ (No. 13), (**c**) ‘Pumpkin Pie’ (No. 19), (**d**) ‘Route 66’ (No. 25), and (**e**) ‘Moonbeam’ (No. 31).

(**a**)

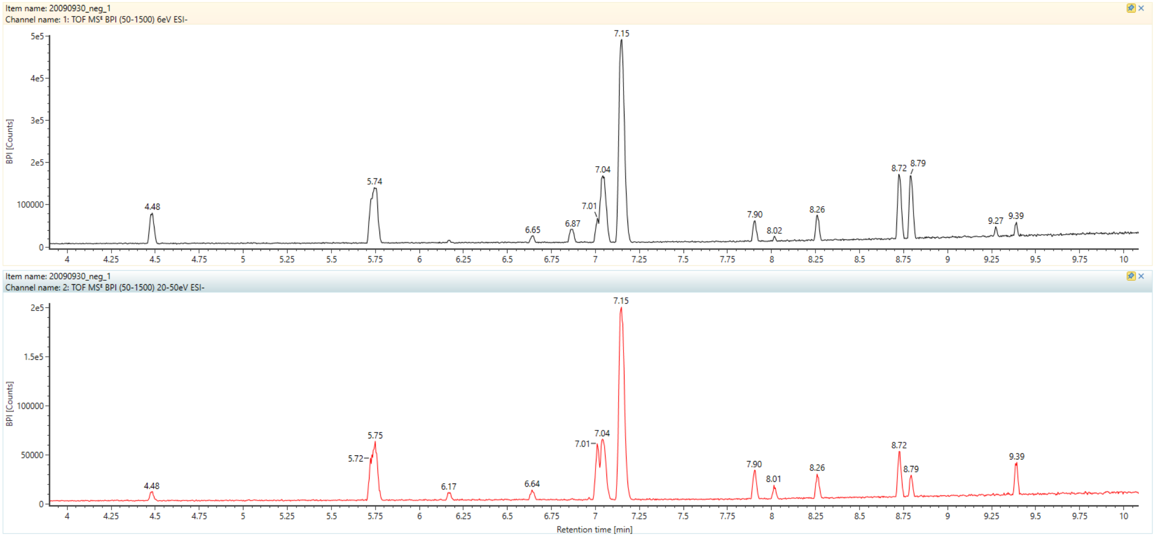

(**b**)

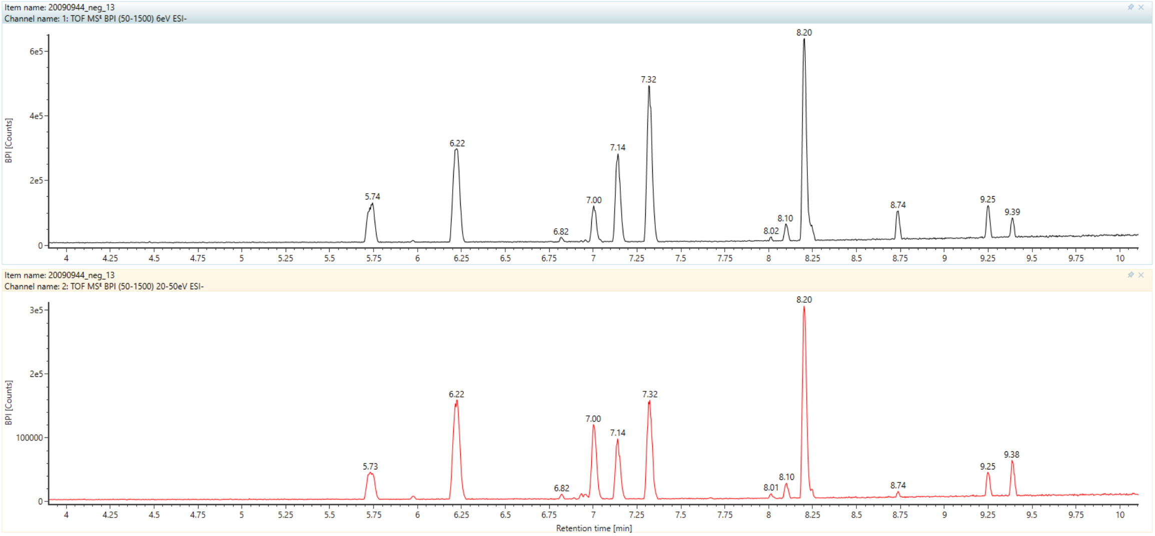

(c)

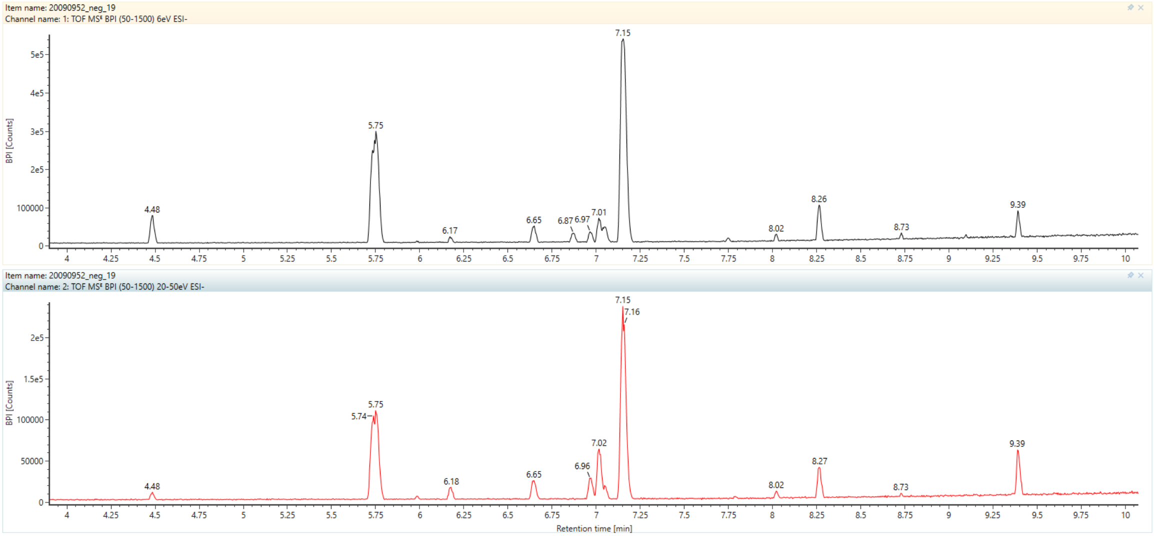
Item name: 20090952_neg_19
Channel name: 1: TOF MS^E BPI (50-1500) 6eV ESI-
BPI [Counts]
4.48
5.75
6.17
6.65
6.87
6.97
7.01
7.15
8.02
8.26
8.73
9.39
Item name: 20090952_neg_19
Channel name: 2: TOF MS^E BPI (50-1500) 20-50eV ESI-
BPI [Counts]
4.48
5.74
5.75
6.18
6.65
6.96
7.02
7.15
7.16
8.02
8.27
8.73
9.39
Retention time [min]

(d)

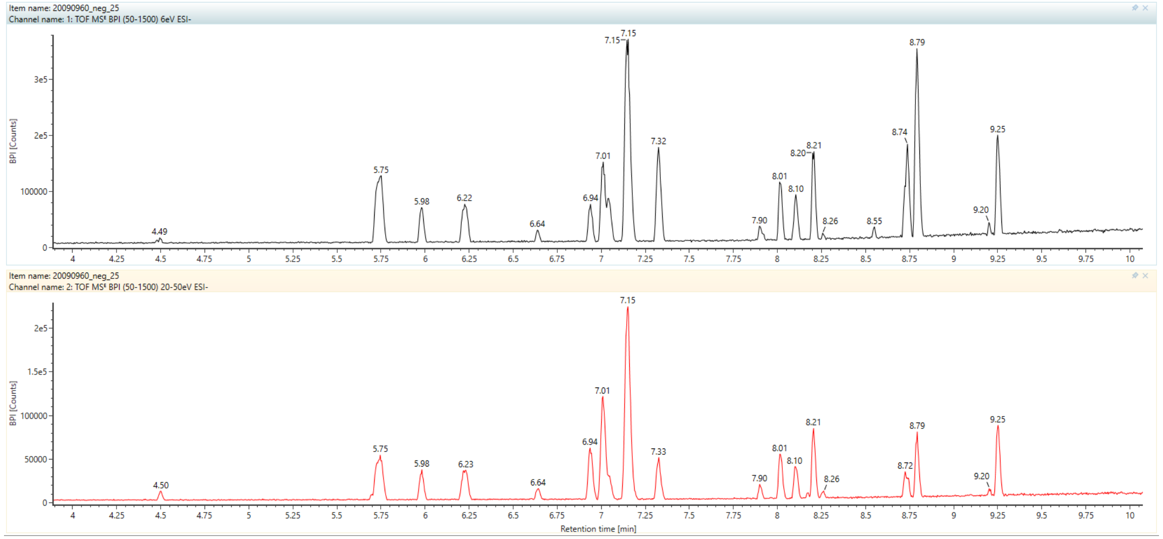
Item name: 20090960_neg_25
Channel name: 1: TOF MS^E BPI (50-1500) 6eV ESI-
BPI [Counts]
4.49
5.75
5.98
6.22
6.64
6.94
7.01
7.15
7.15
7.32
7.90
8.01
8.10
8.20
8.21
8.26
8.55
8.74
8.79
9.20
9.25
Item name: 20090960_neg_25
Channel name: 2: TOF MS^E BPI (50-1500) 20-50eV ESI-
BPI [Counts]
4.50
5.75
5.98
6.23
6.64
6.94
7.01
7.15
7.33
7.90
8.01
8.10
8.21
8.26
8.72
8.79
9.20
9.25
Retention time [min]

(e)

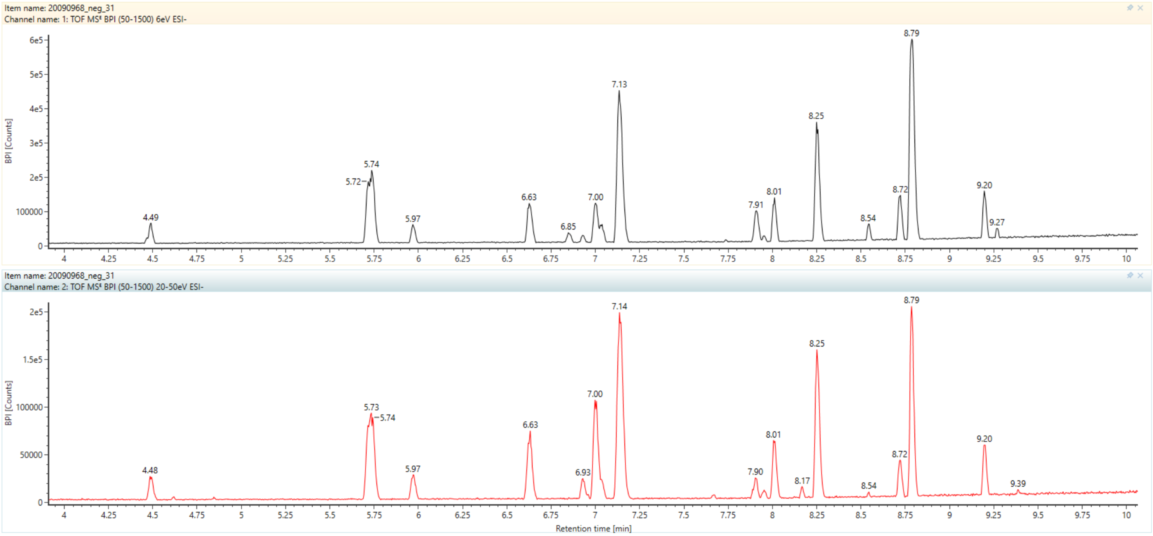
Item name: 20090968_neg_31
Channel name: 1: TOF MS^E BPI (50-1500) 6eV ESI-
BPI [Counts]
Item name: 20090968_neg_31
Channel name: 2: TOF MS^E BPI (50-1500) 20-50eV ESI-
BPI [Counts]
Retention time [min]

Figure S2. ESI-QTof-MS spectrum of taxifolin-7-O-glucoside (peak 1)

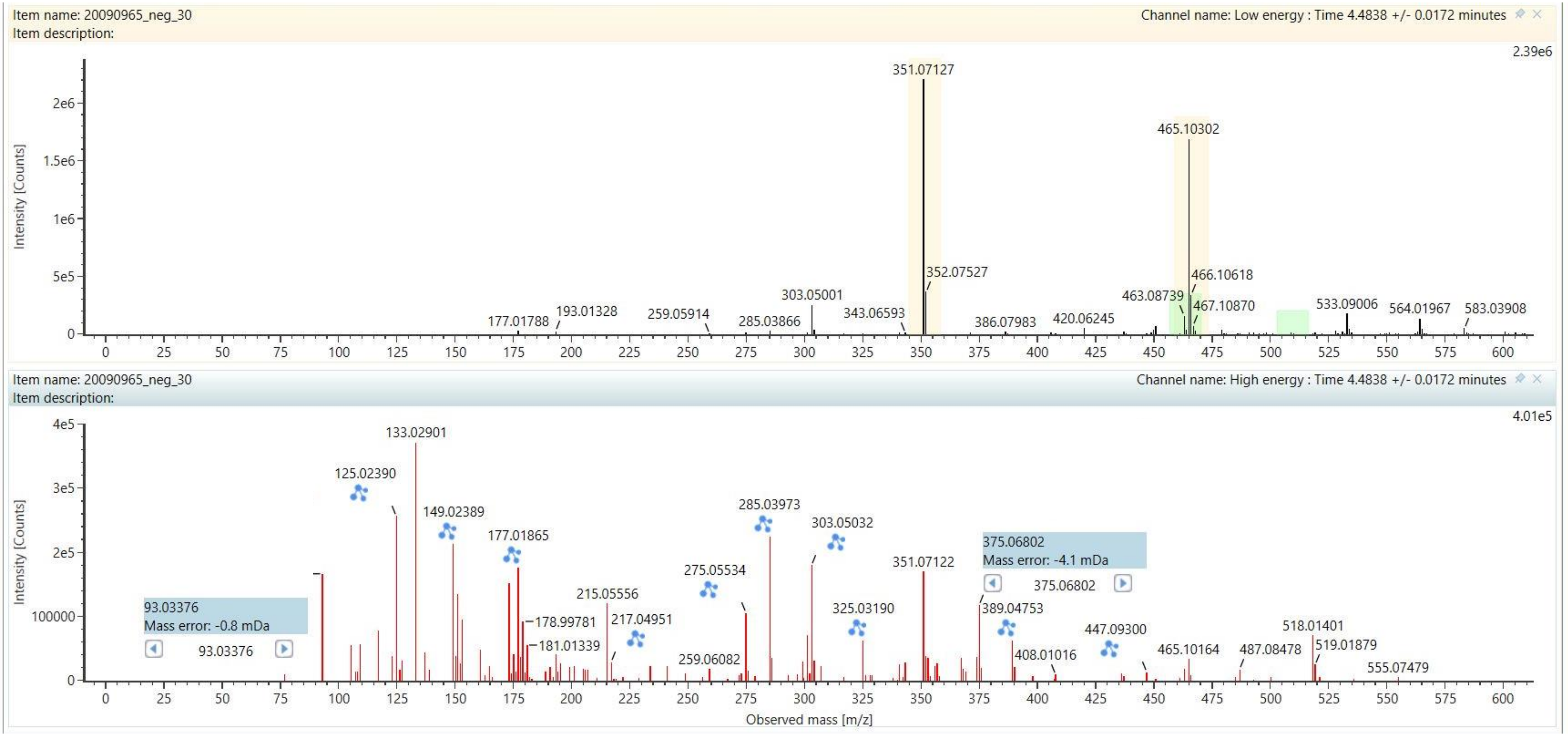

Figure S3. ESI-QTof-MS spectrum of chlorogenic acid (peak 2)

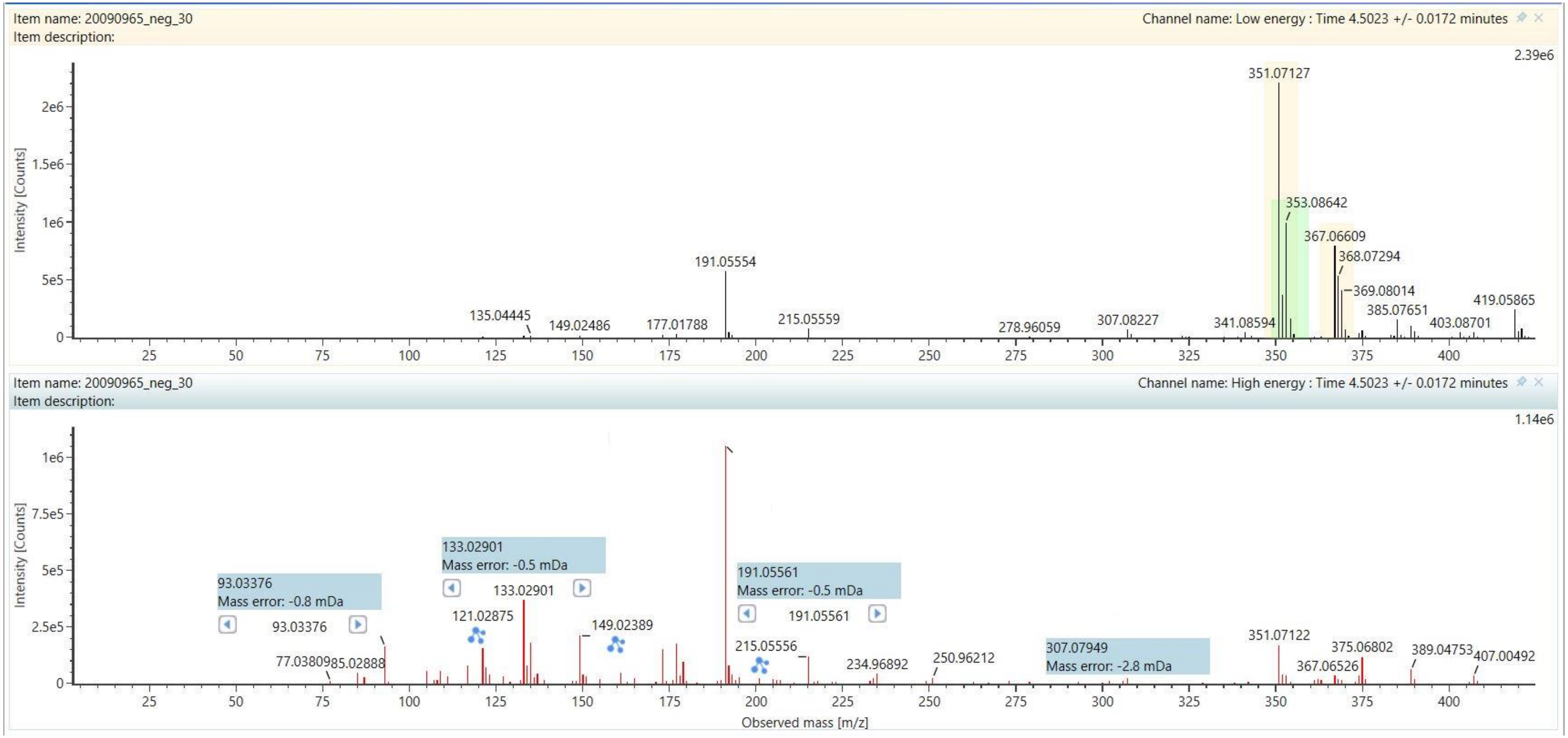

Figure S4. ESI-QTof-MS spectrum of taxifolin-3-O-glucoside (peak 3)

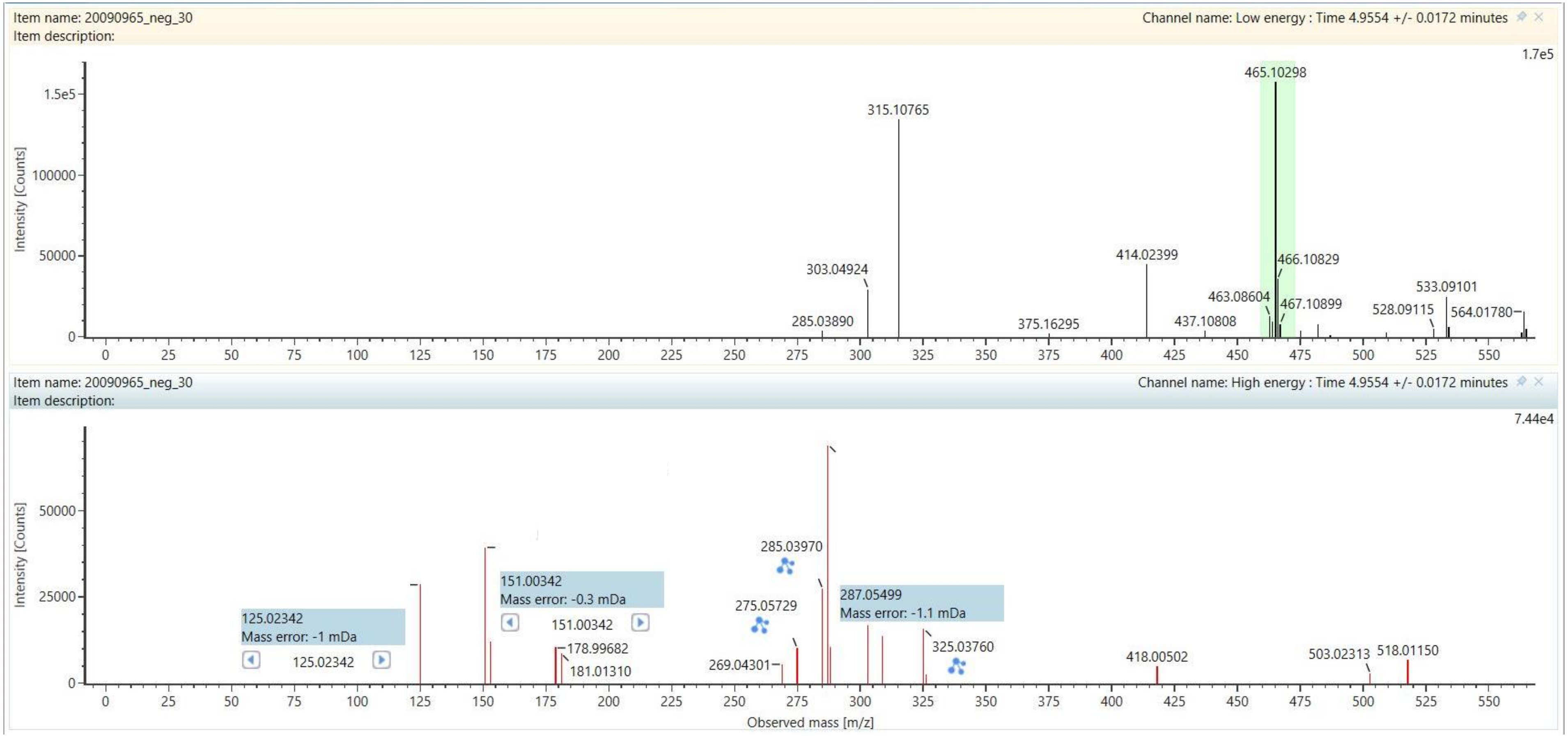

Figure S5. ESI-QTof-MS spectrum of vanillic acid-4-glucoside (peak 4)

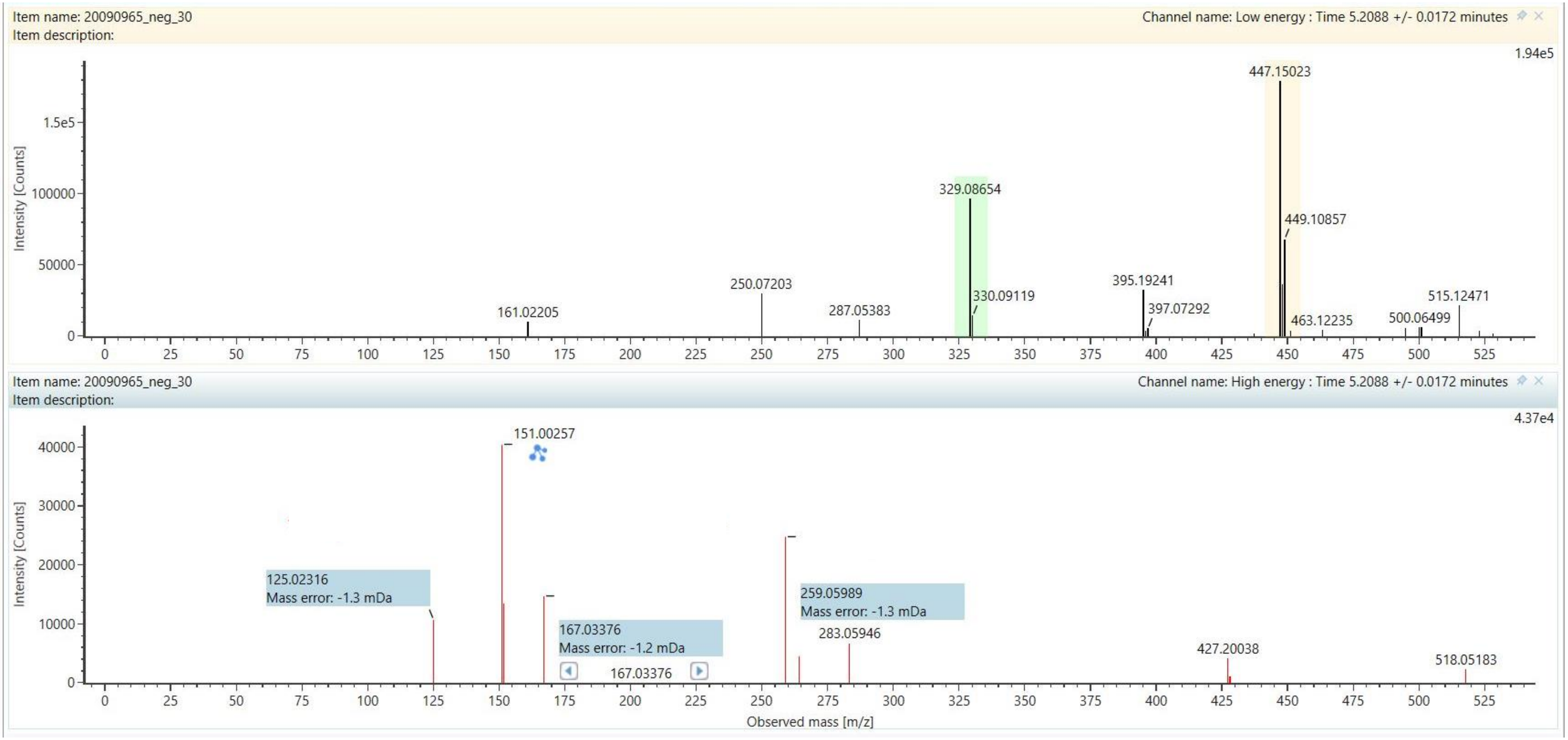

Figure S6. ESI-QTof-MS spectrum of flavomarein (peak 5)

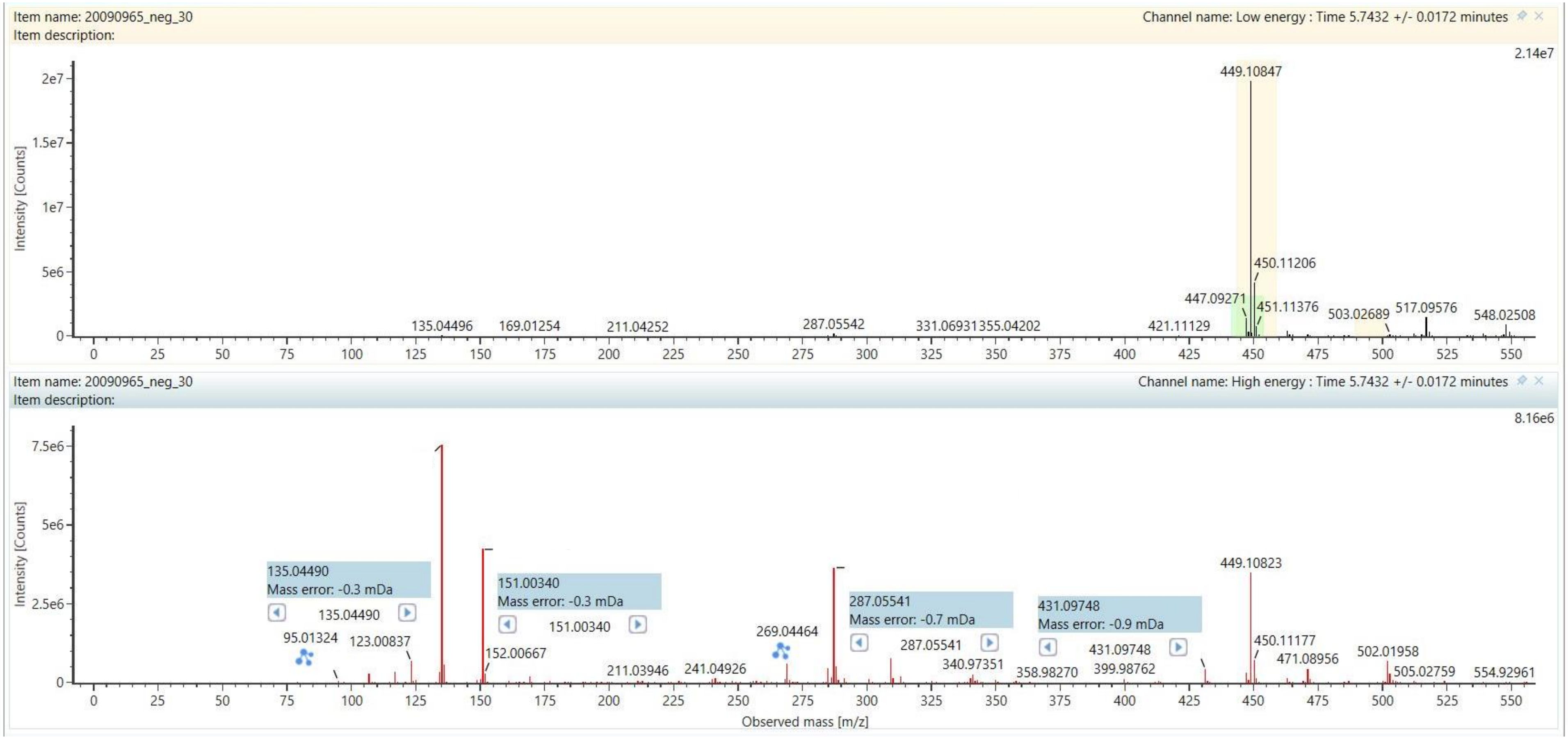

Figure S7. ESI-QTof-MS spectrum of isookanin-7-O-rutinoside (peak 6)

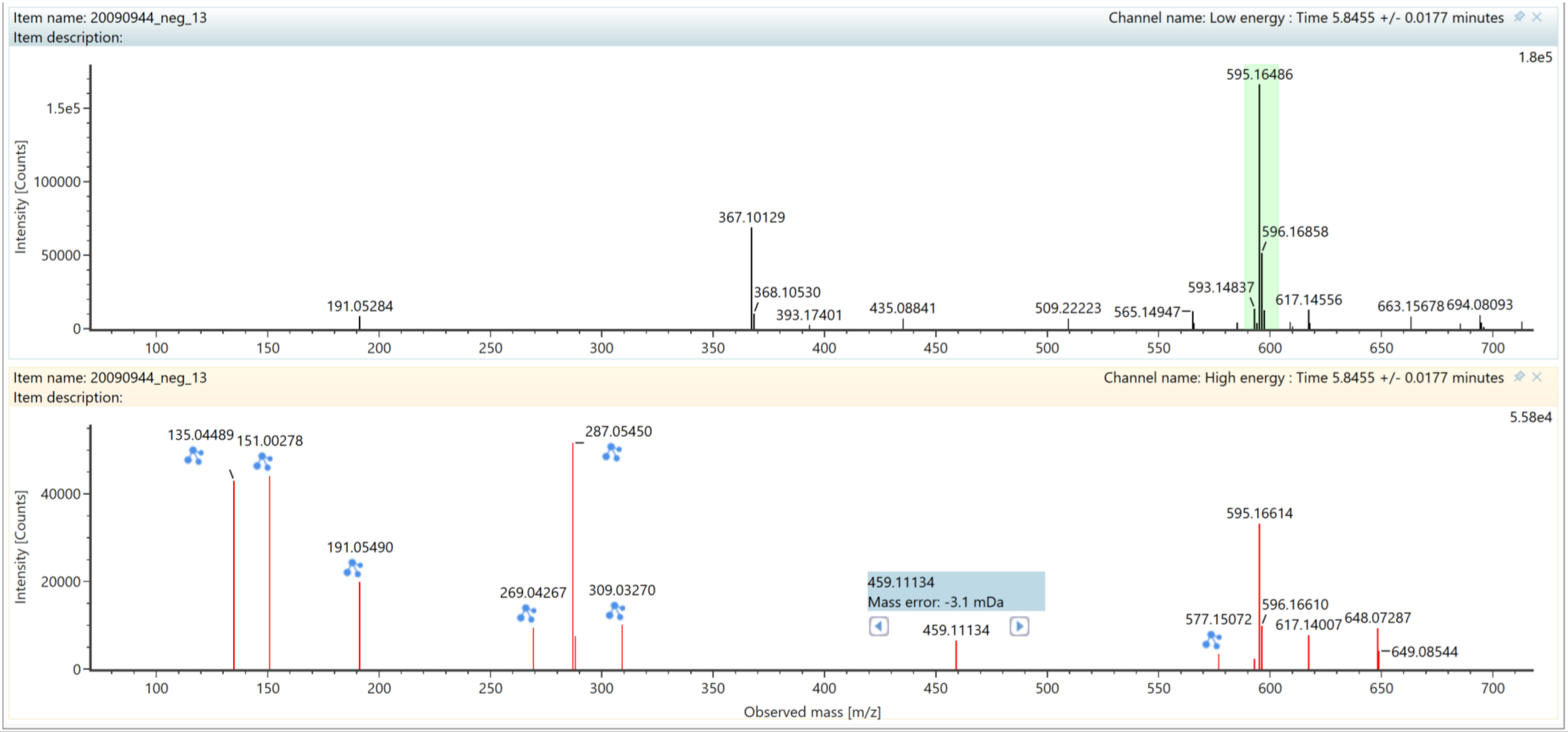

Figure S8. ESI-QTof-MS spectrum of luteolin-7-O-sophoroside (peak 7)

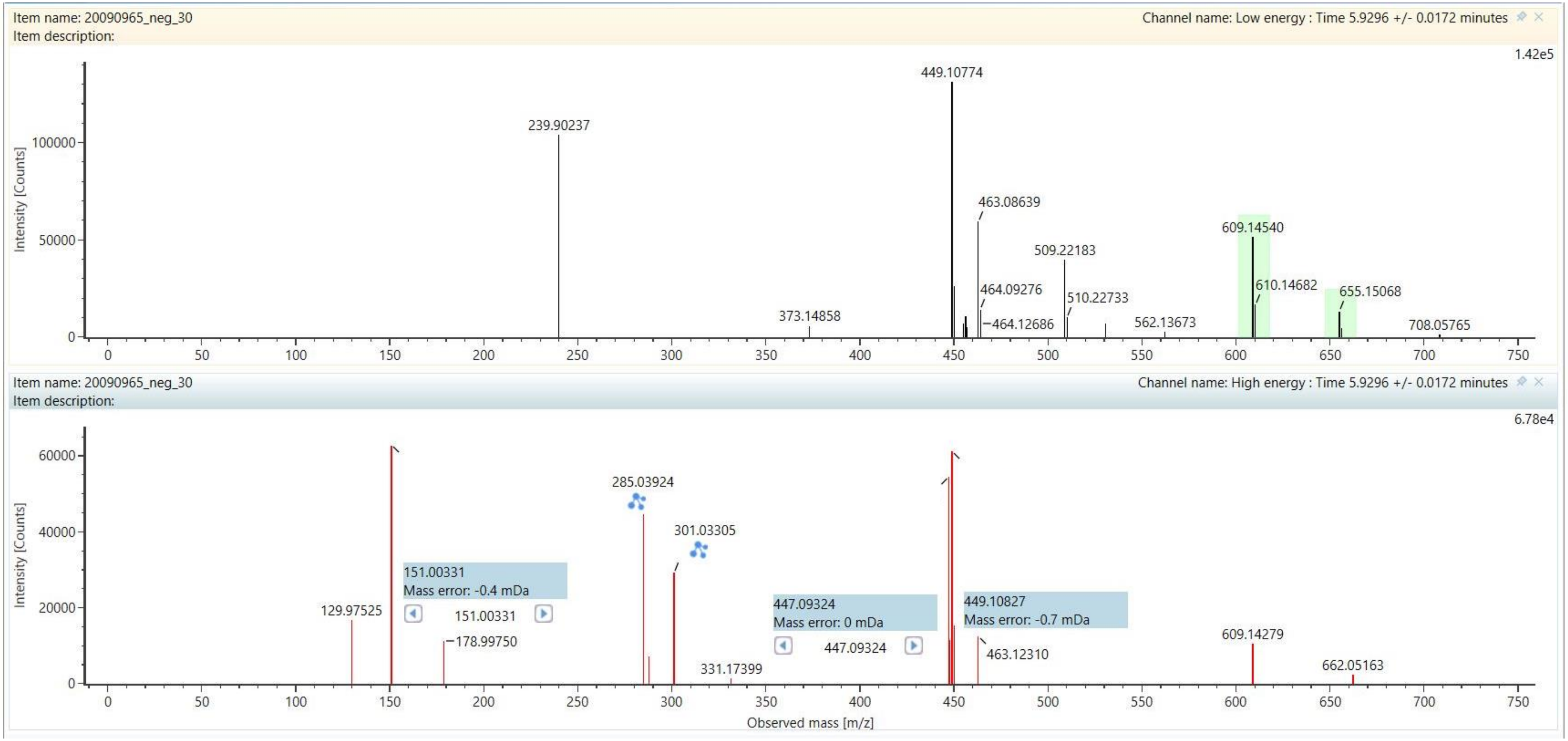

Figure S9. ESI-QTof-MS spectrum of butin-7-O-glucoside (peak 8)

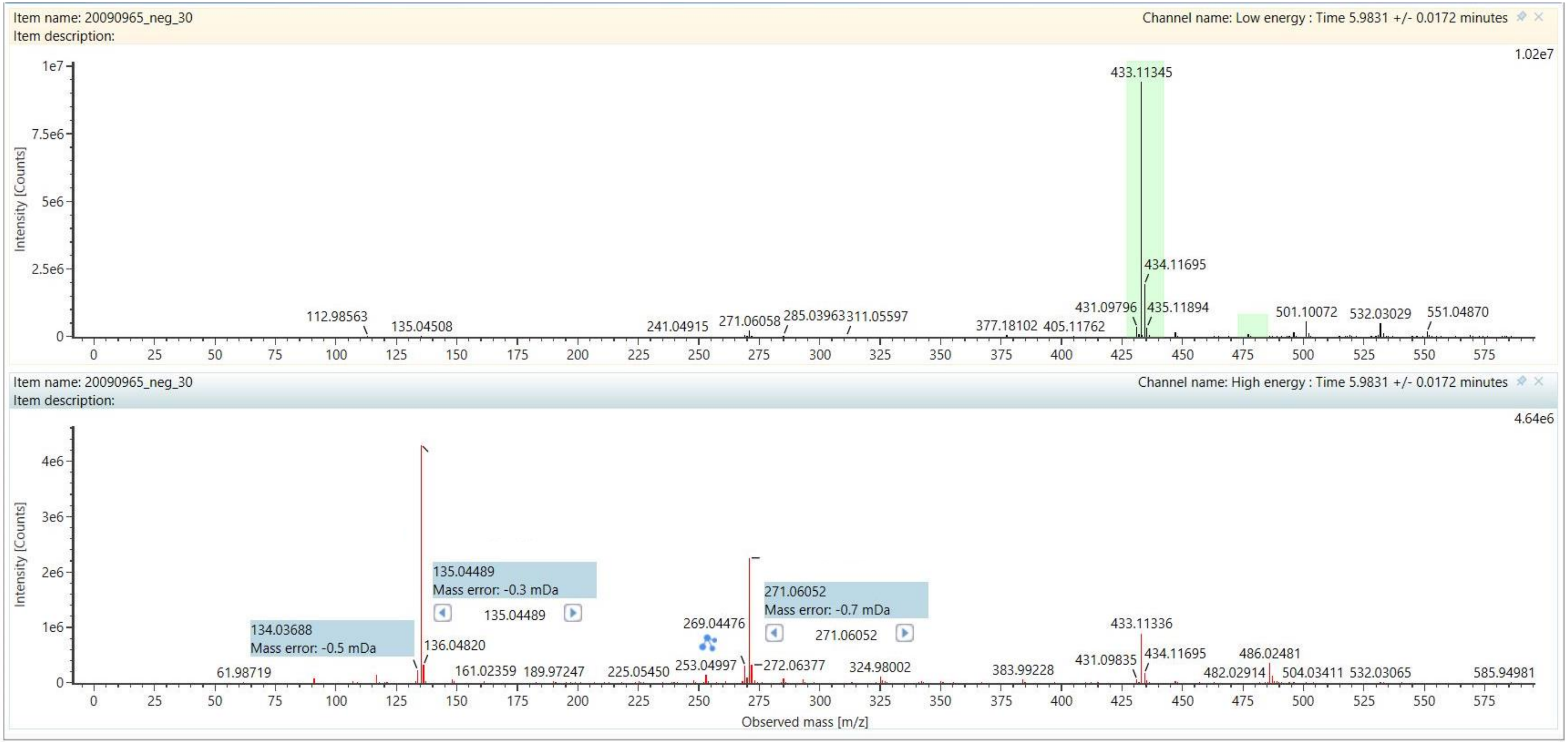

Figure S10. ESI-QTof-MS spectrum of 8-methoxyeriodictyol-7-O-glucoside (peak 9)

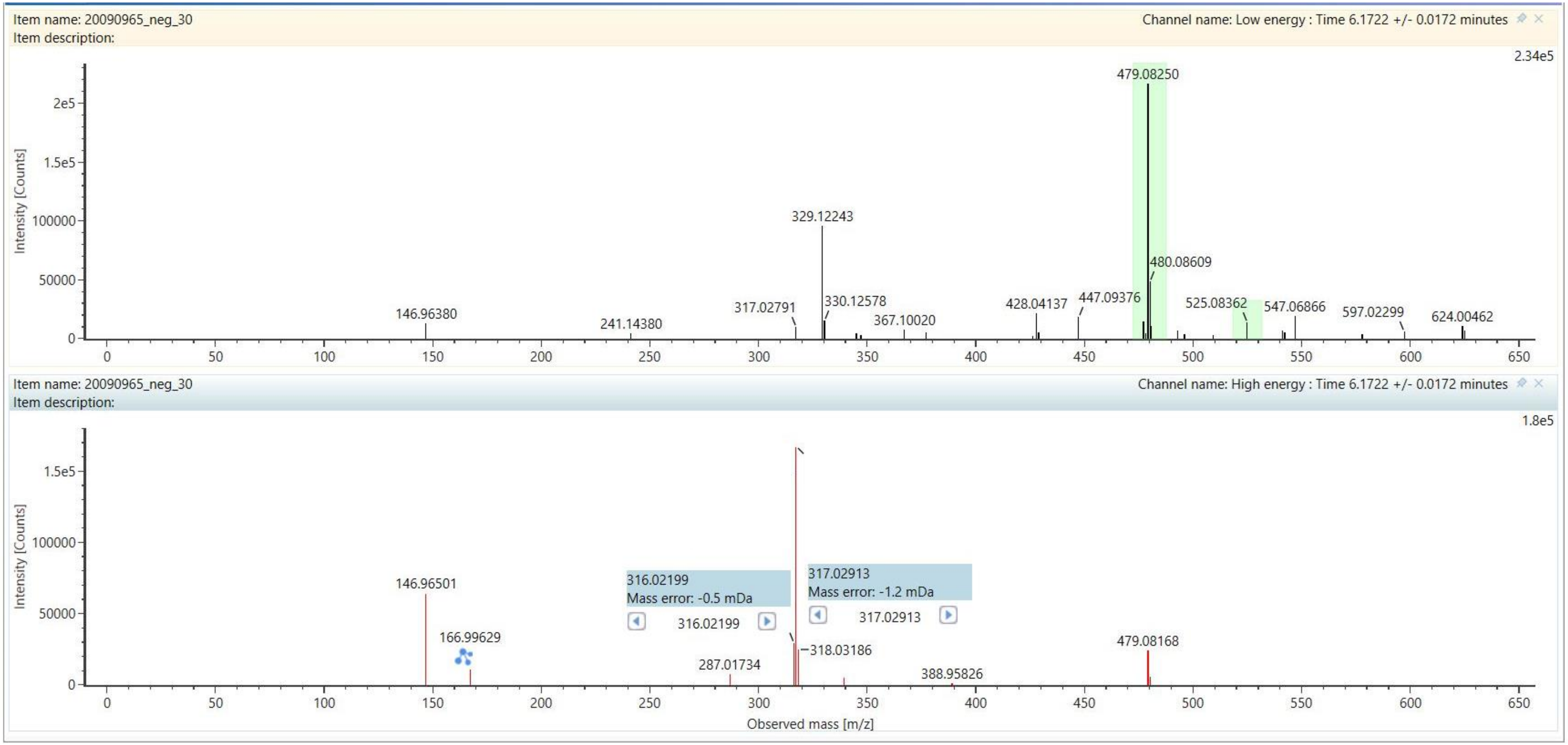

Figure S11. ESI-QTof-MS spectrum of coreolanceoline B (peak 10)

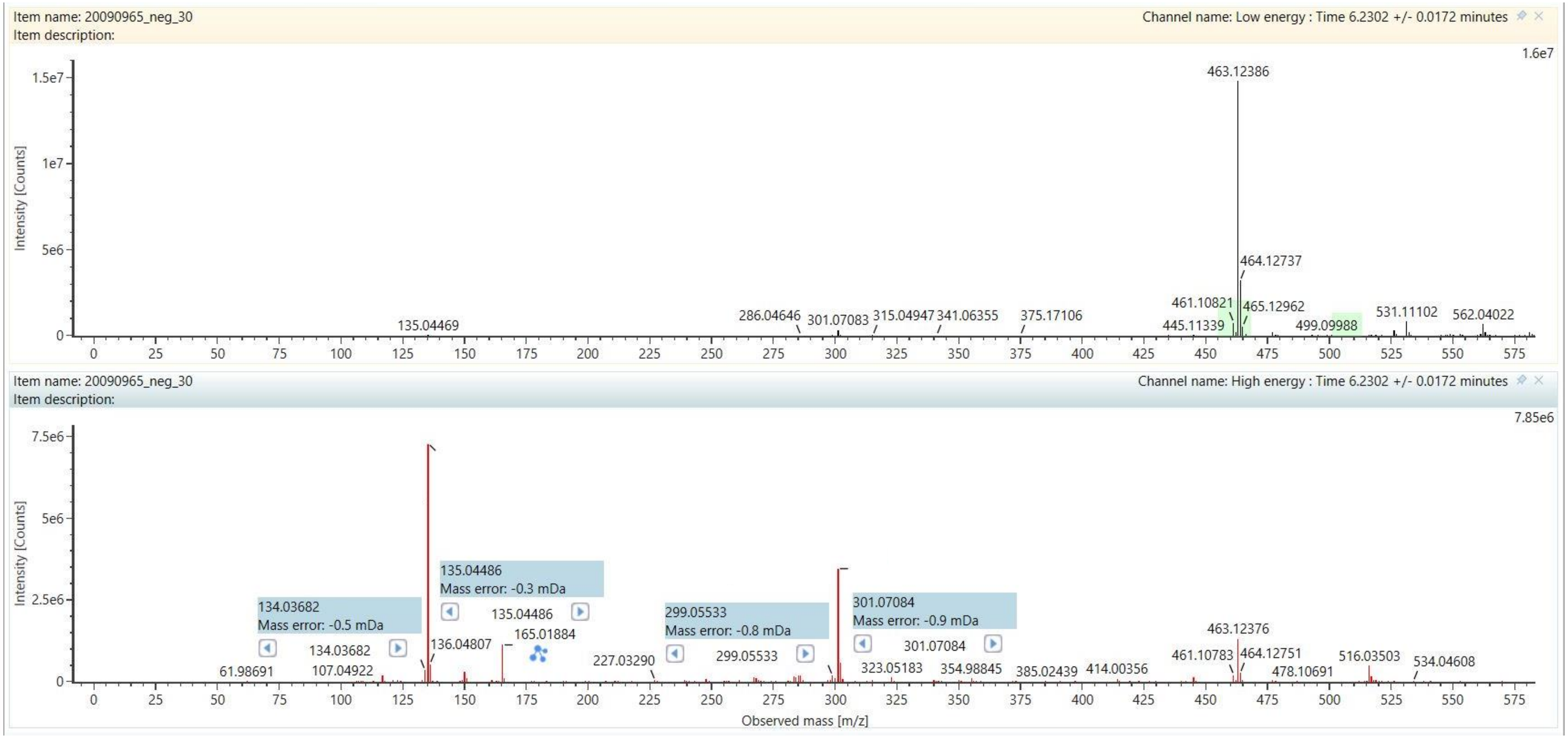

Figure S12. ESI-QTof-MS spectrum of lanceolin (peak 11)

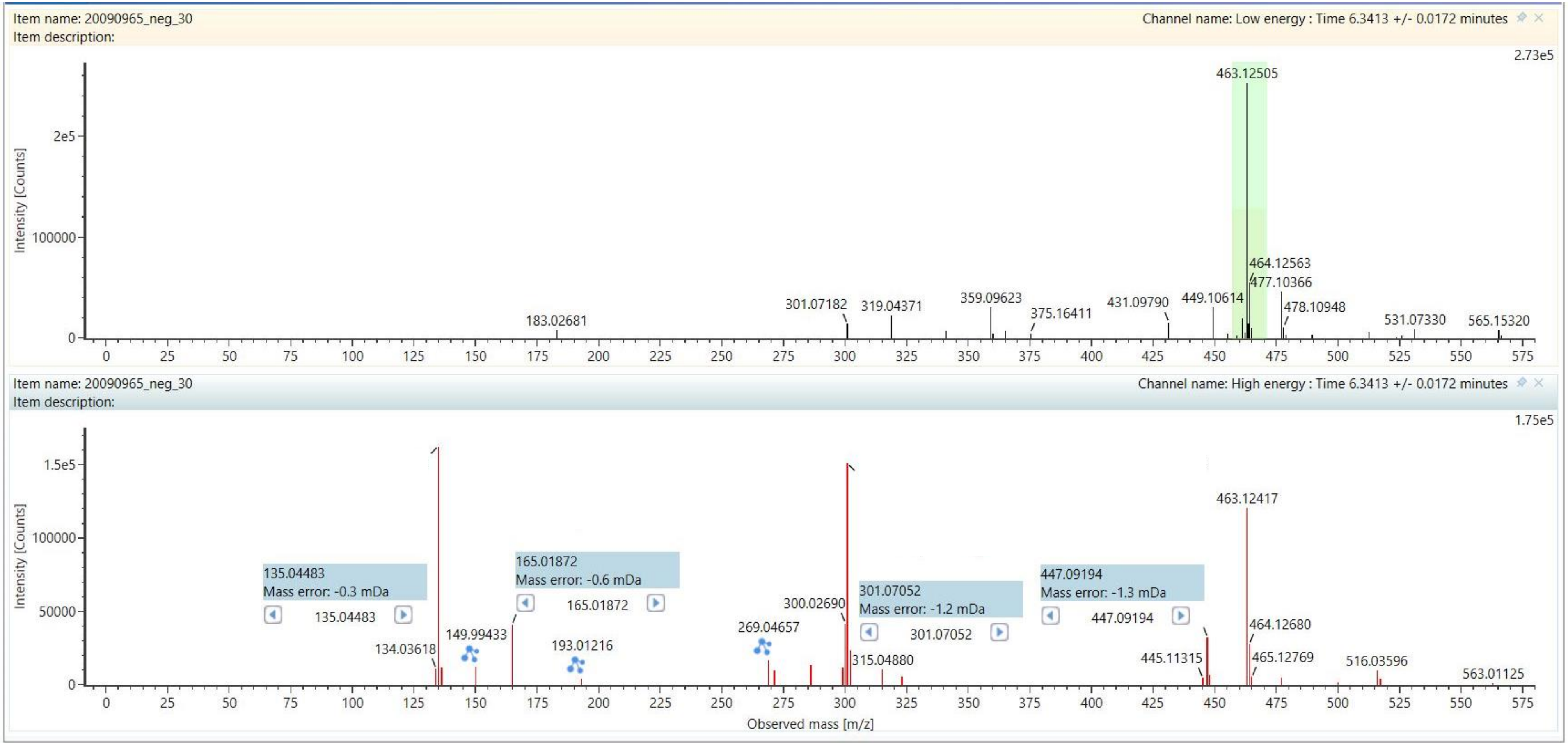

Figure S13. ESI-QTof-MS spectrum of naringenin-7-O-glucoside (peak 12)

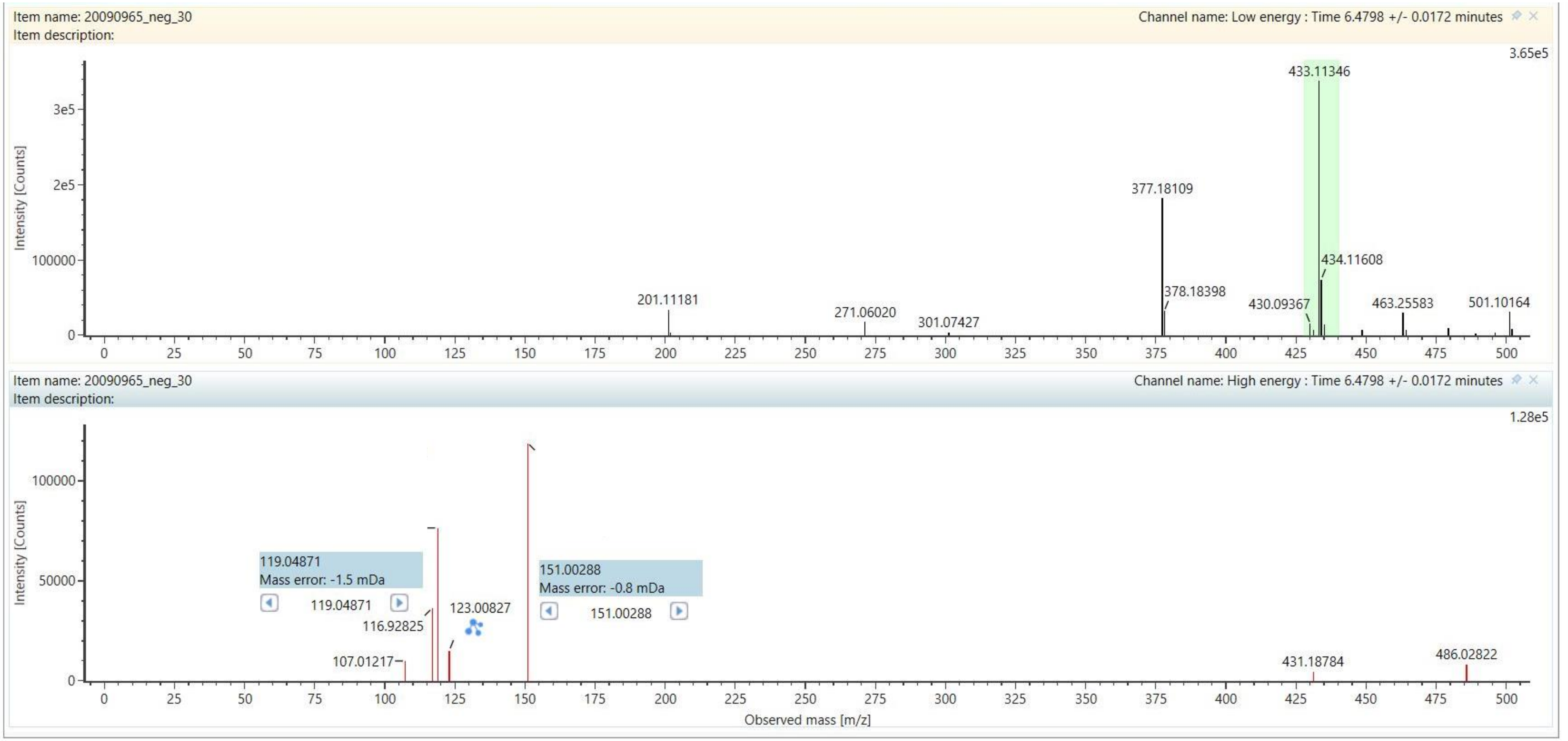

Figure S14. ESI-QTof-MS spectrum of okanin-4,4′-O-diglucoside (peak 13)

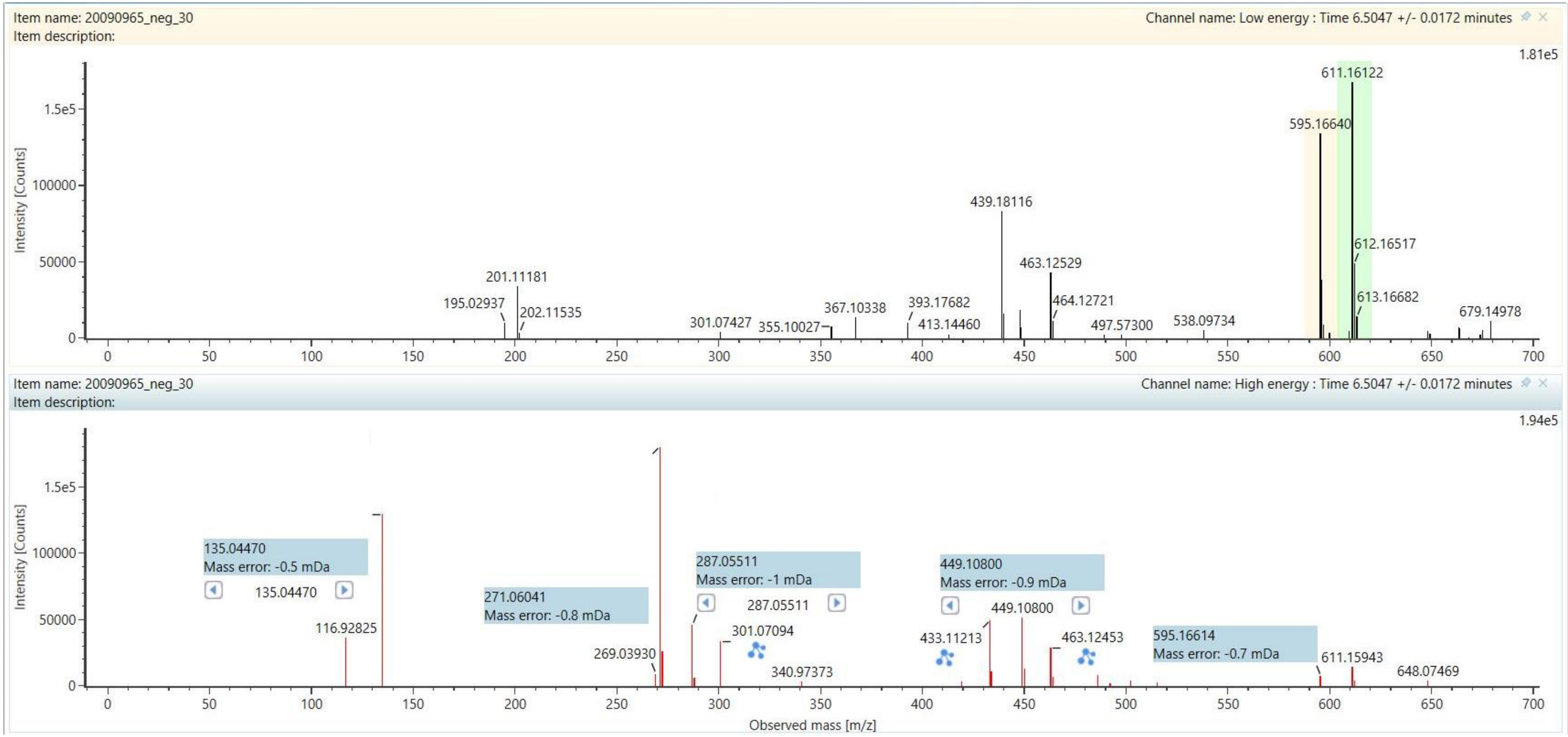

Figure S15. ESI-QTof-MS spectrum of 4′,7,8-trihydroxyflavone-O-diglucoside (peak 14)

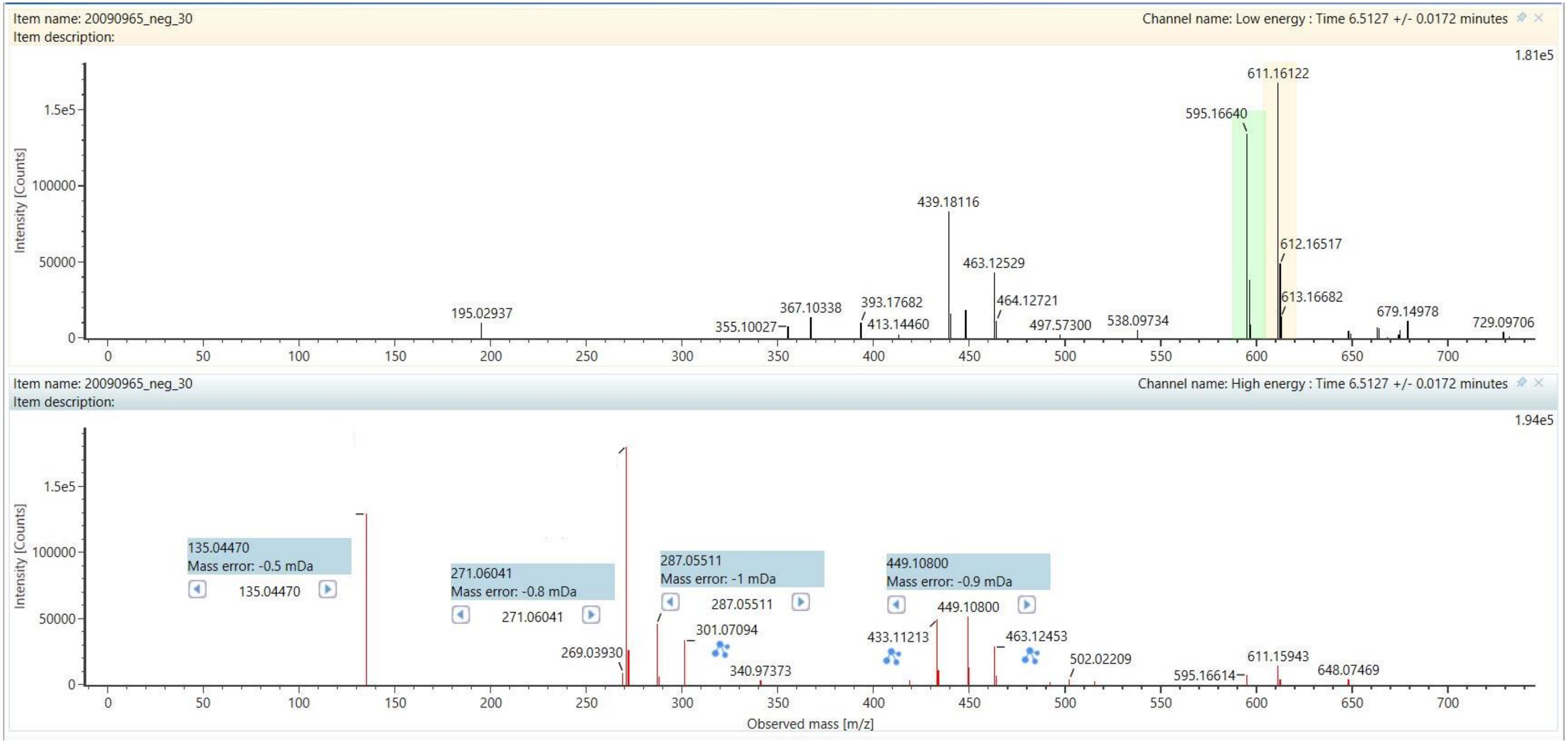

Figure S16. ESI-QTof-MS spectrum of fisetin-3,7-O-diglucoside (peak 15)

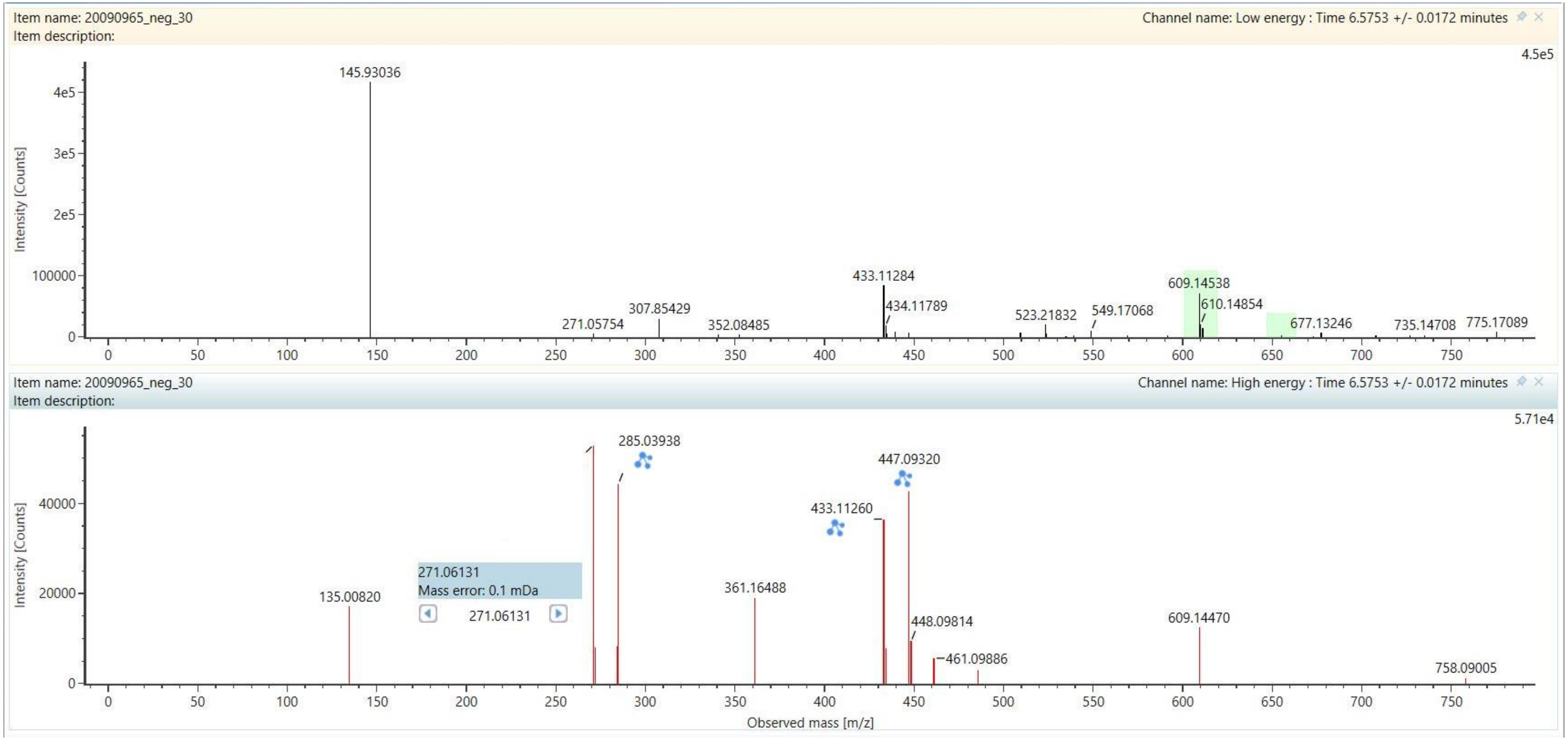

Figure S17. ESI-QTof-MS spectrum of isookanin (peak 16)

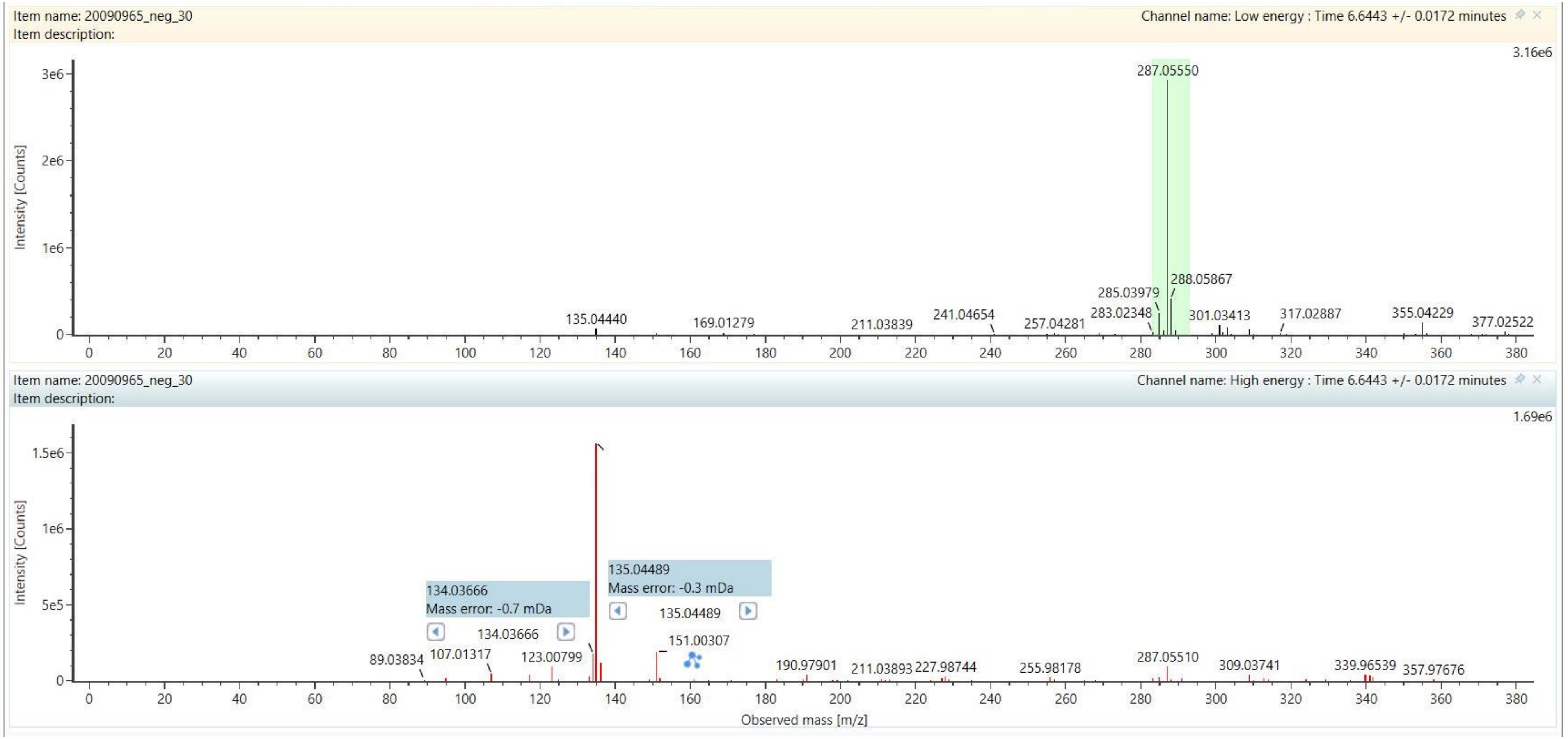

Figure S18. ESI-QTof-MS spectrum of taxifolin (peak 17)

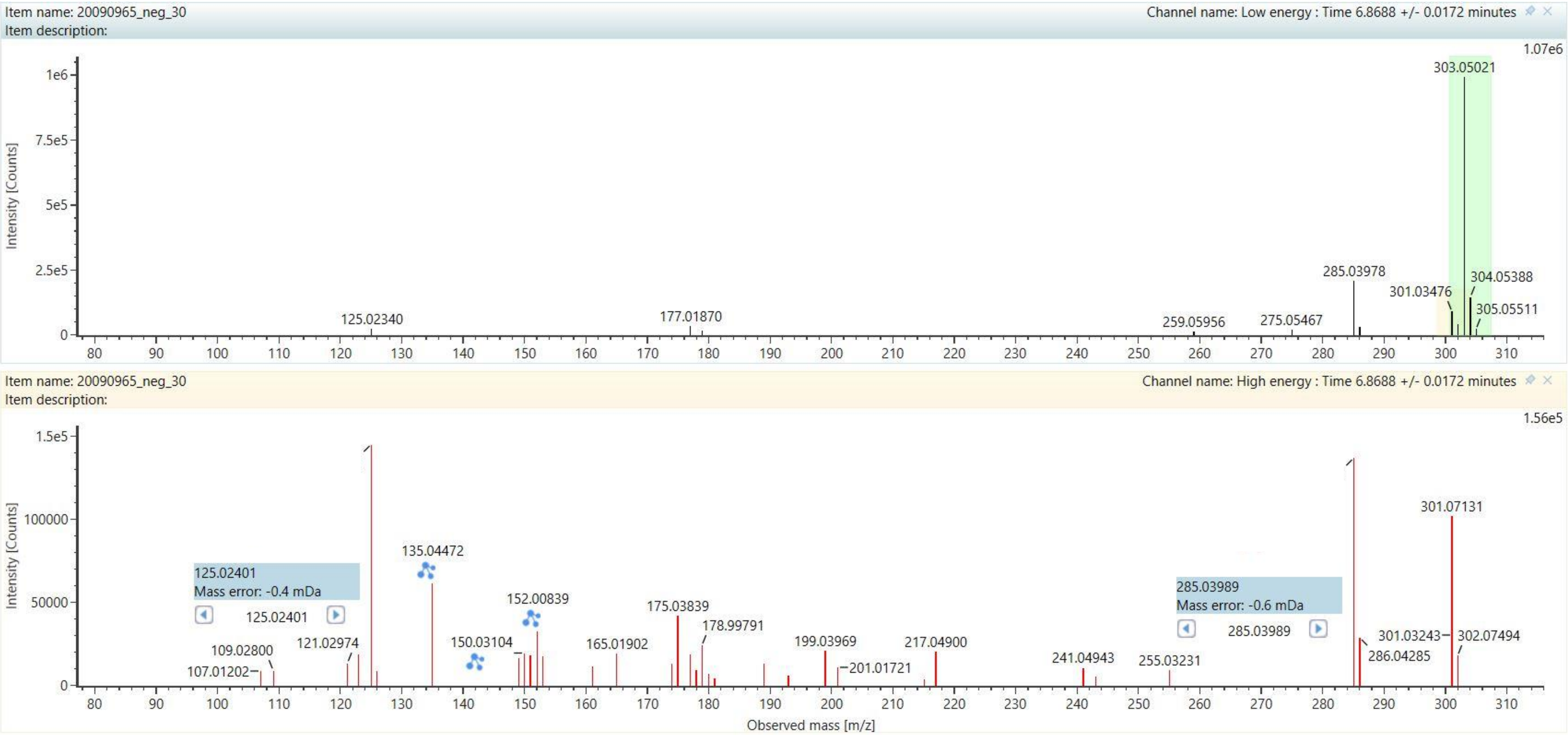

Figure S19. ESI-QTof-MS spectrum of 4′,5,7,8-tetrahydroxyflavanone-7-O-(6-O-arabinosyl-glucoside) (peak 18)

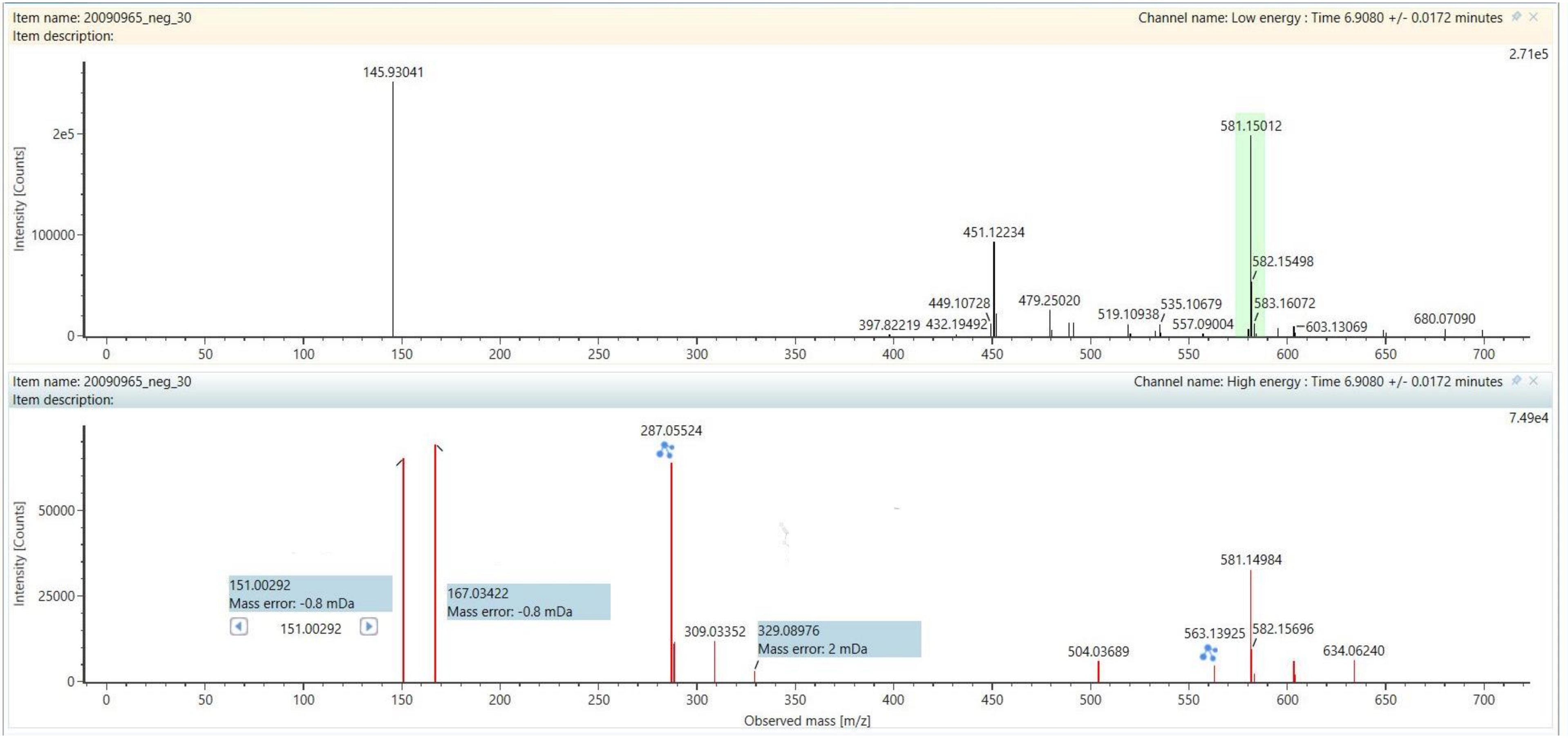

Figure S20. ESI-QTof-MS spectrum of sulfuretin-6-O-glucoside (peak 19)

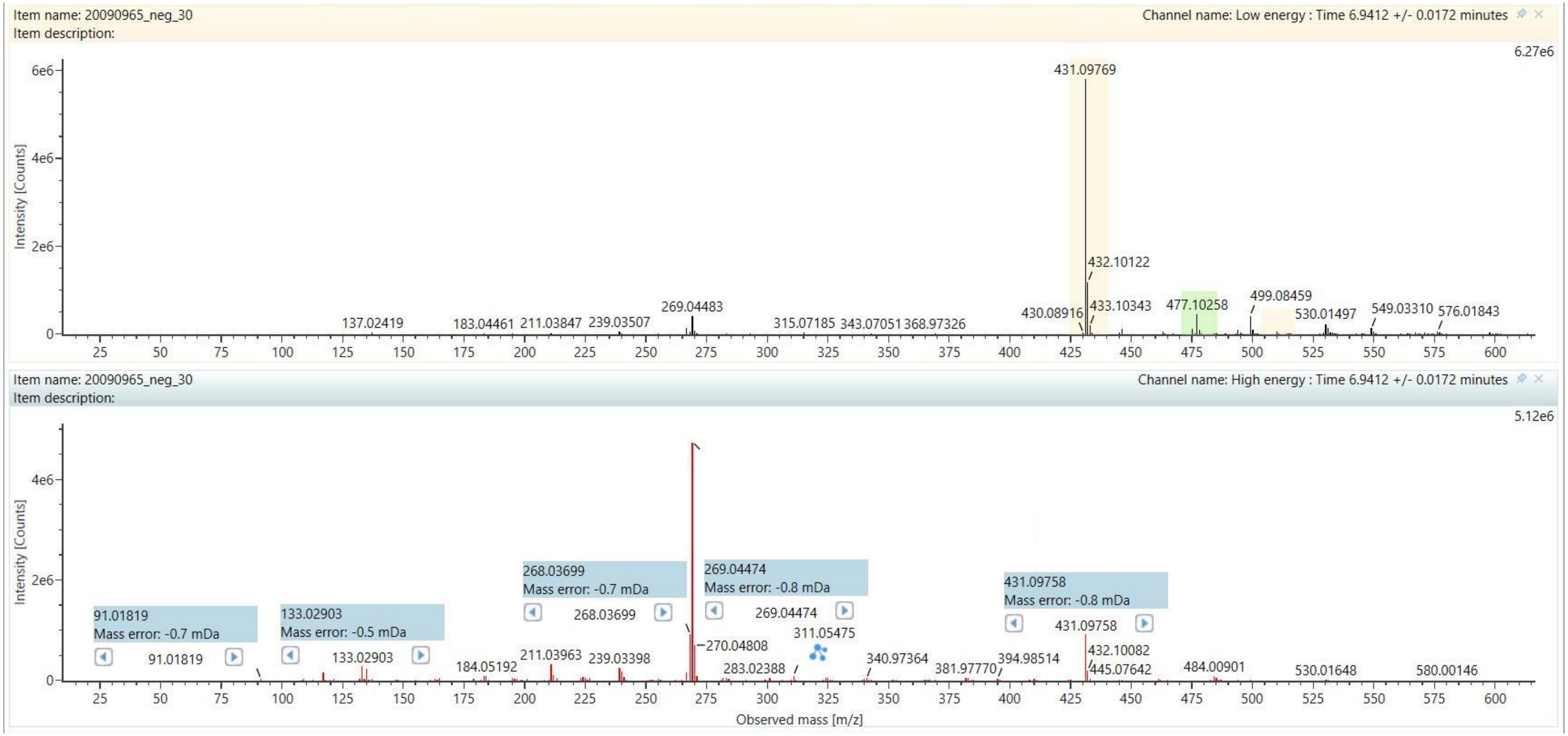

Figure S21. ESI-QTof-MS spectrum of quercetin-7-O-glucoside (peak 20)

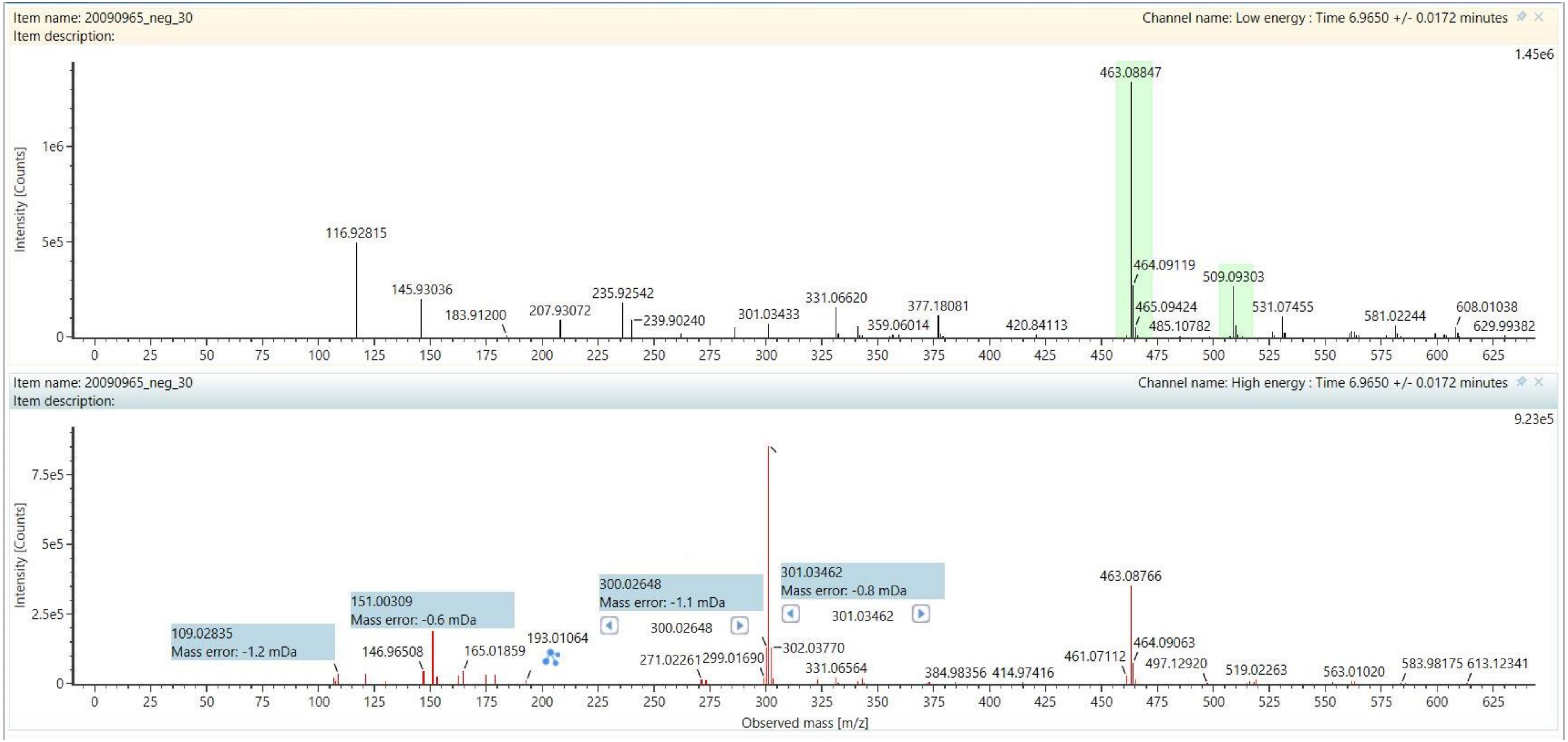

Figure S22. ESI-QTof-MS spectrum of maritimein (peak 21)

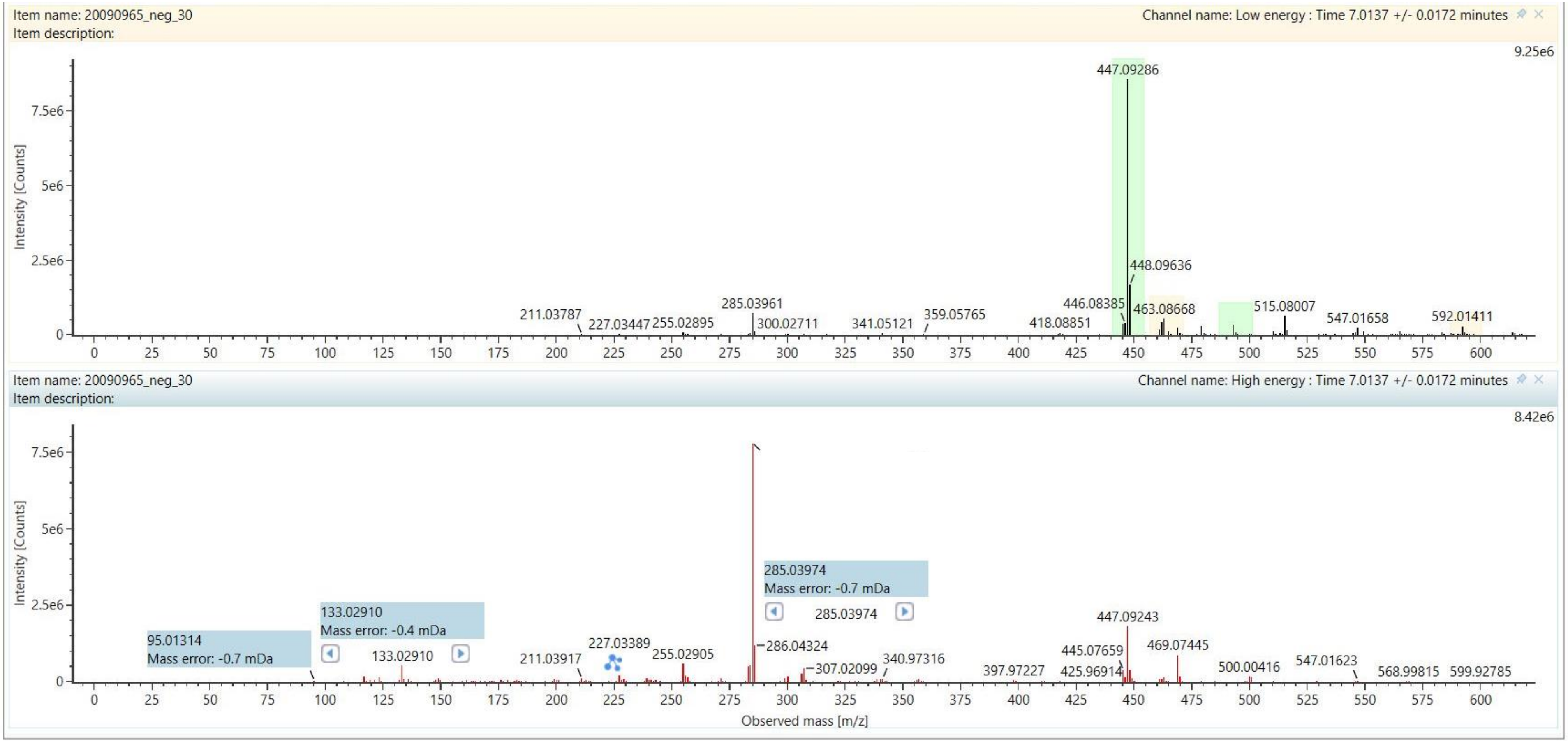

Figure S23. ESI-QTof-MS spectrum of luteolin-7-O-glucoside (peak 22)

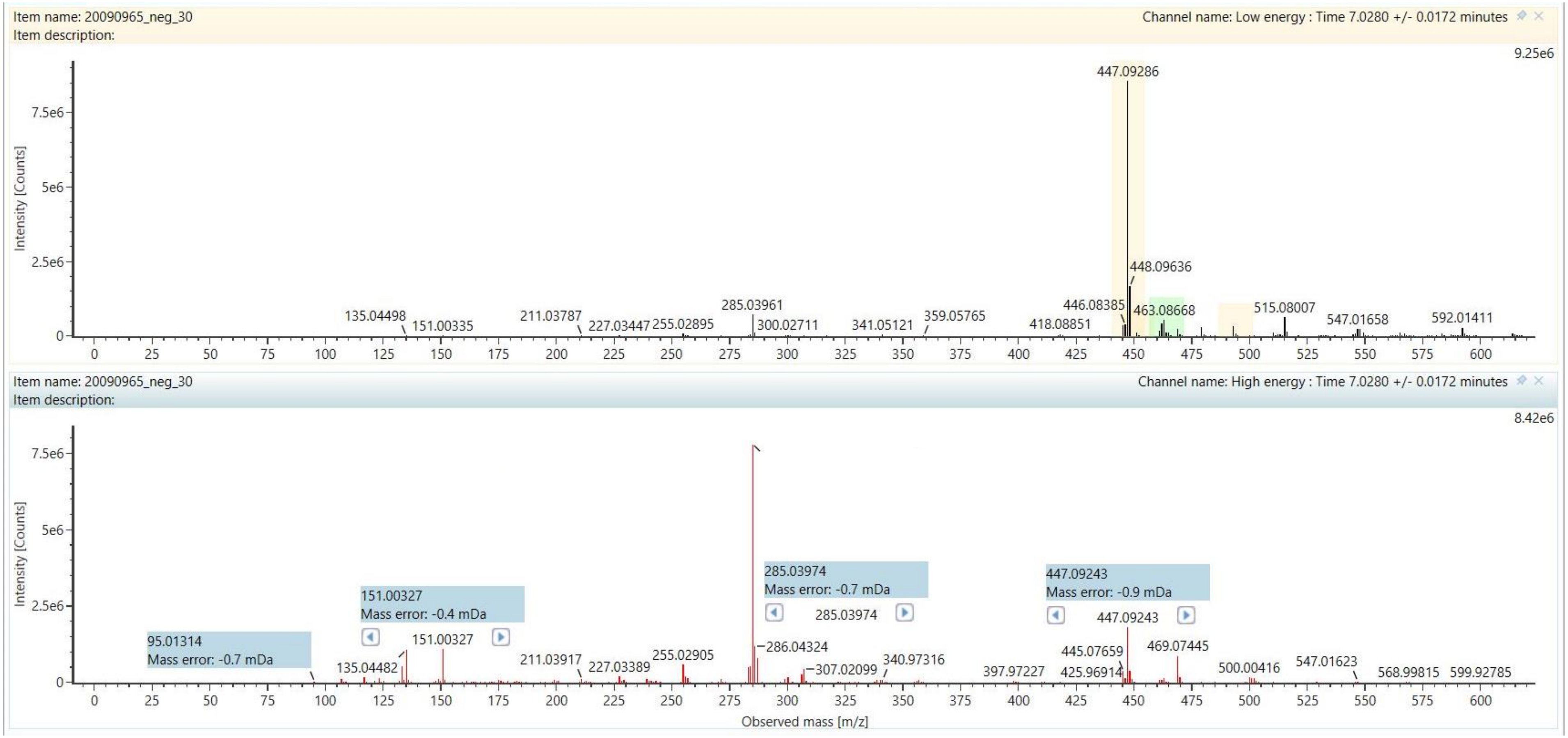

Figure S24. ESI-QTof-MS spectrum of marein (peak23)

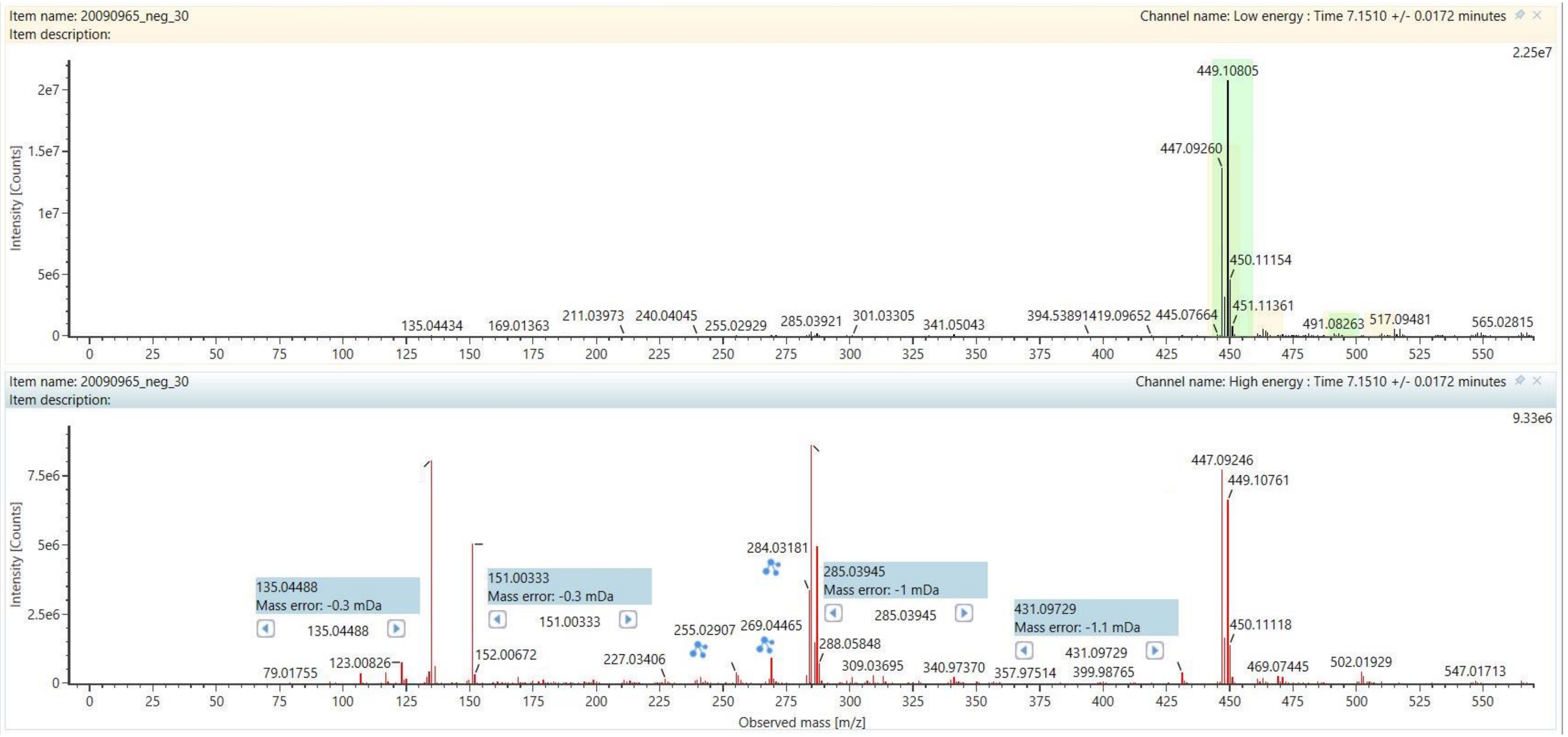

Figure S25. ESI-QTof-MS spectrum of taxifolin 3′,7-dimethyl ether 3-O-glucoside (peak 24)

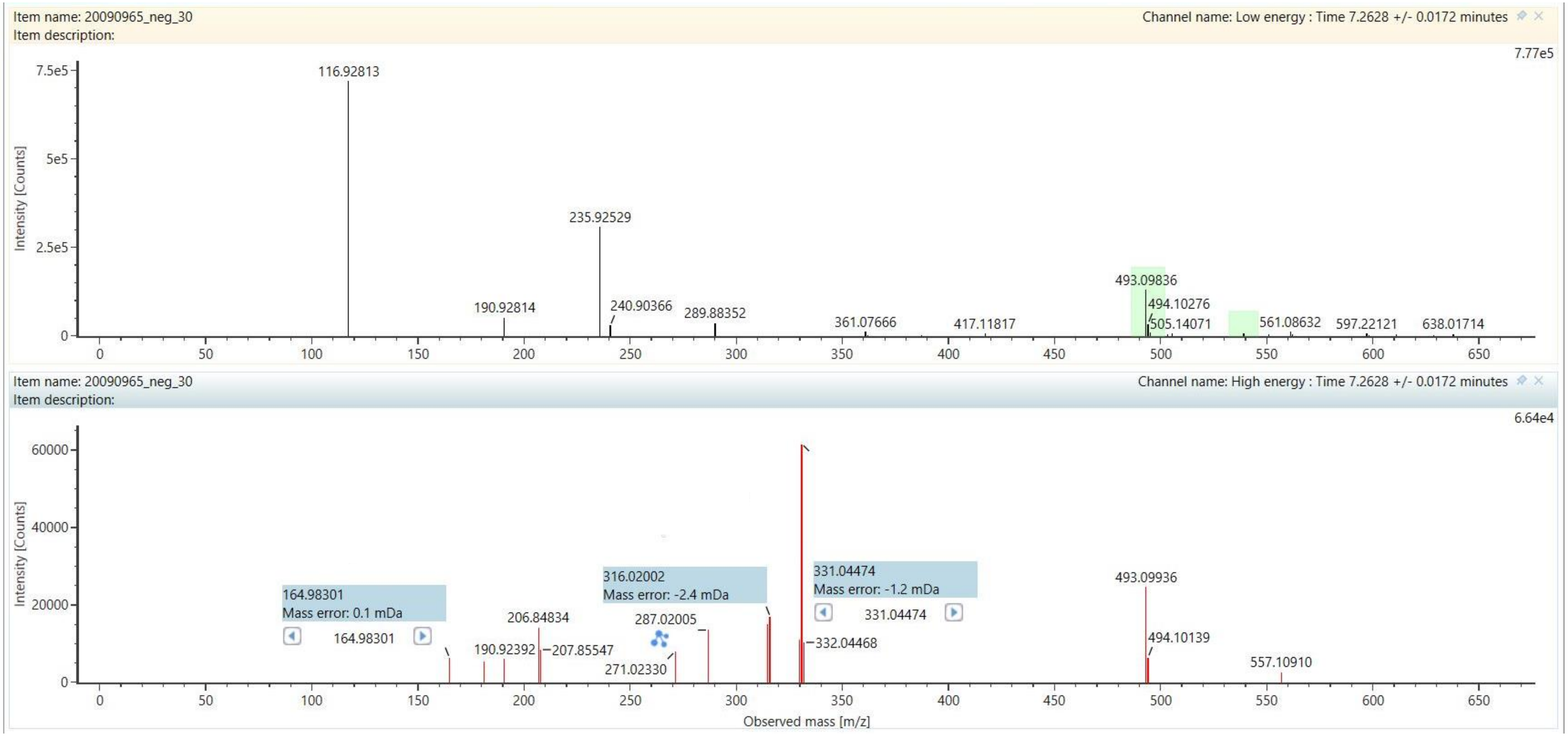

Figure S26. ESI-QTof-MS spectrum of 3,3′,4′-trihydroxy-7-methoxyflavone 3-O-glucoside (peak 25)

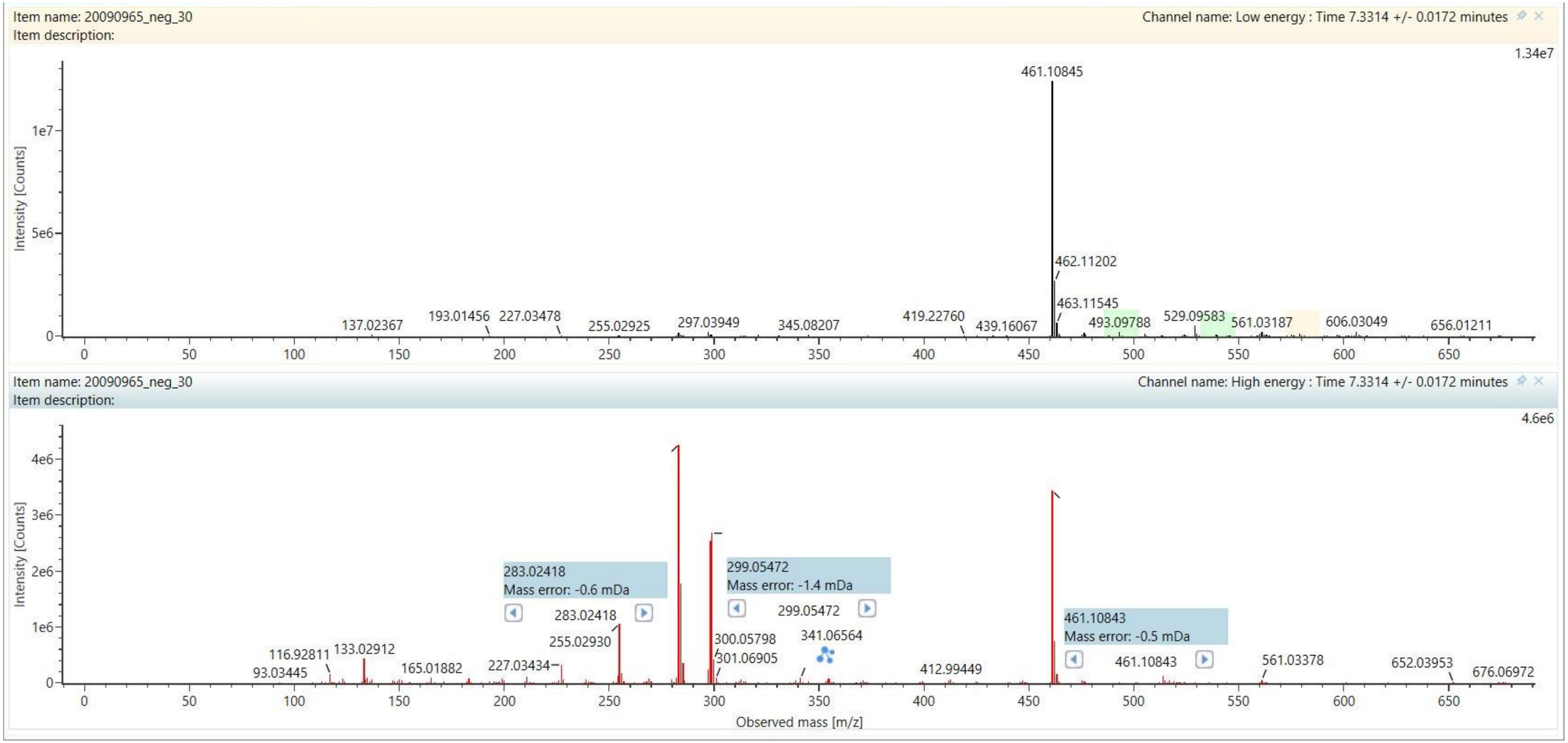

Figure S27. ESI-QTof-MS spectrum of qurcetagetin-7-O-(6″-caffeoylglucoside) (peak 26)

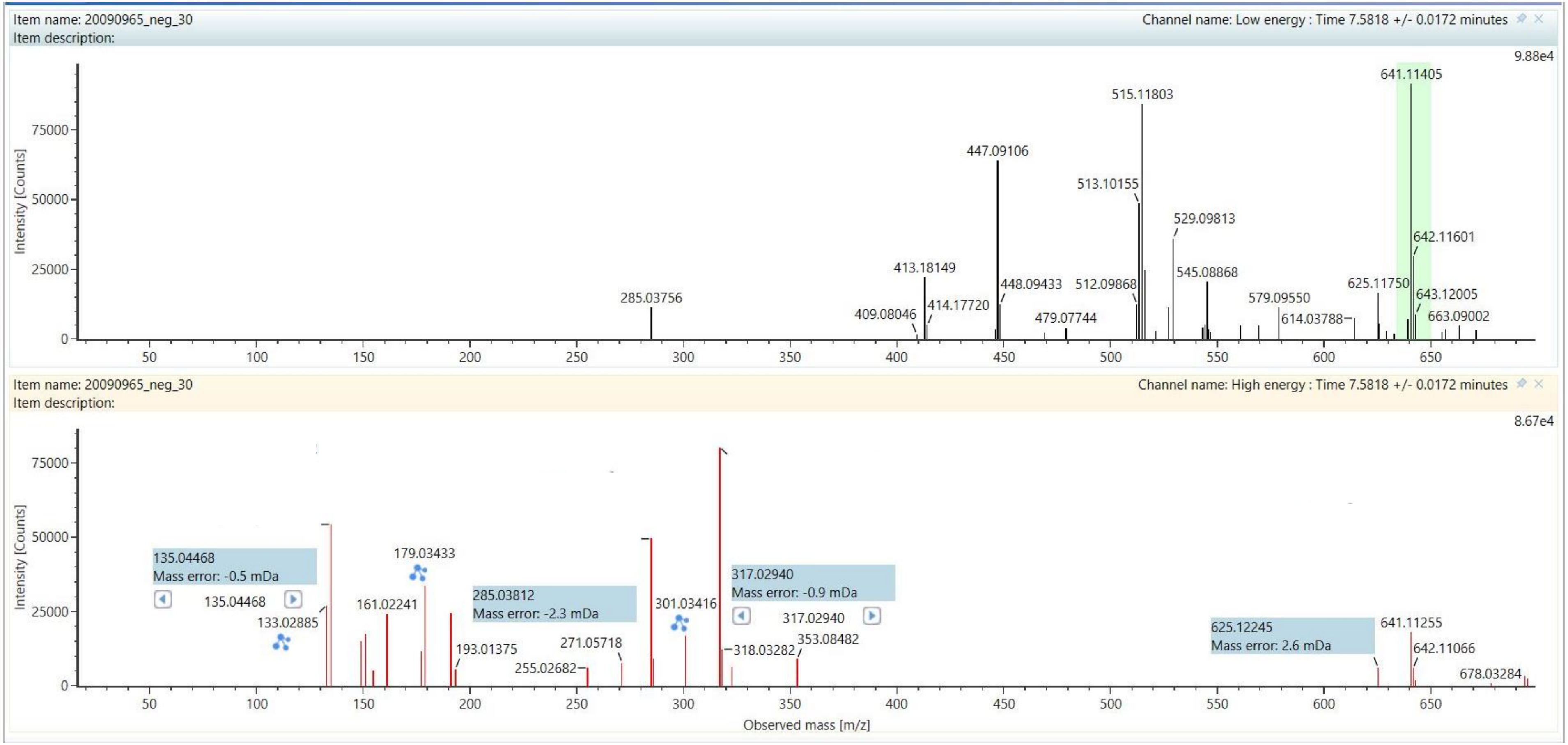

Figure S28. ESI-QTof-MS spectrum of 3,5-dicaffeoylquinic acid (peak 27)

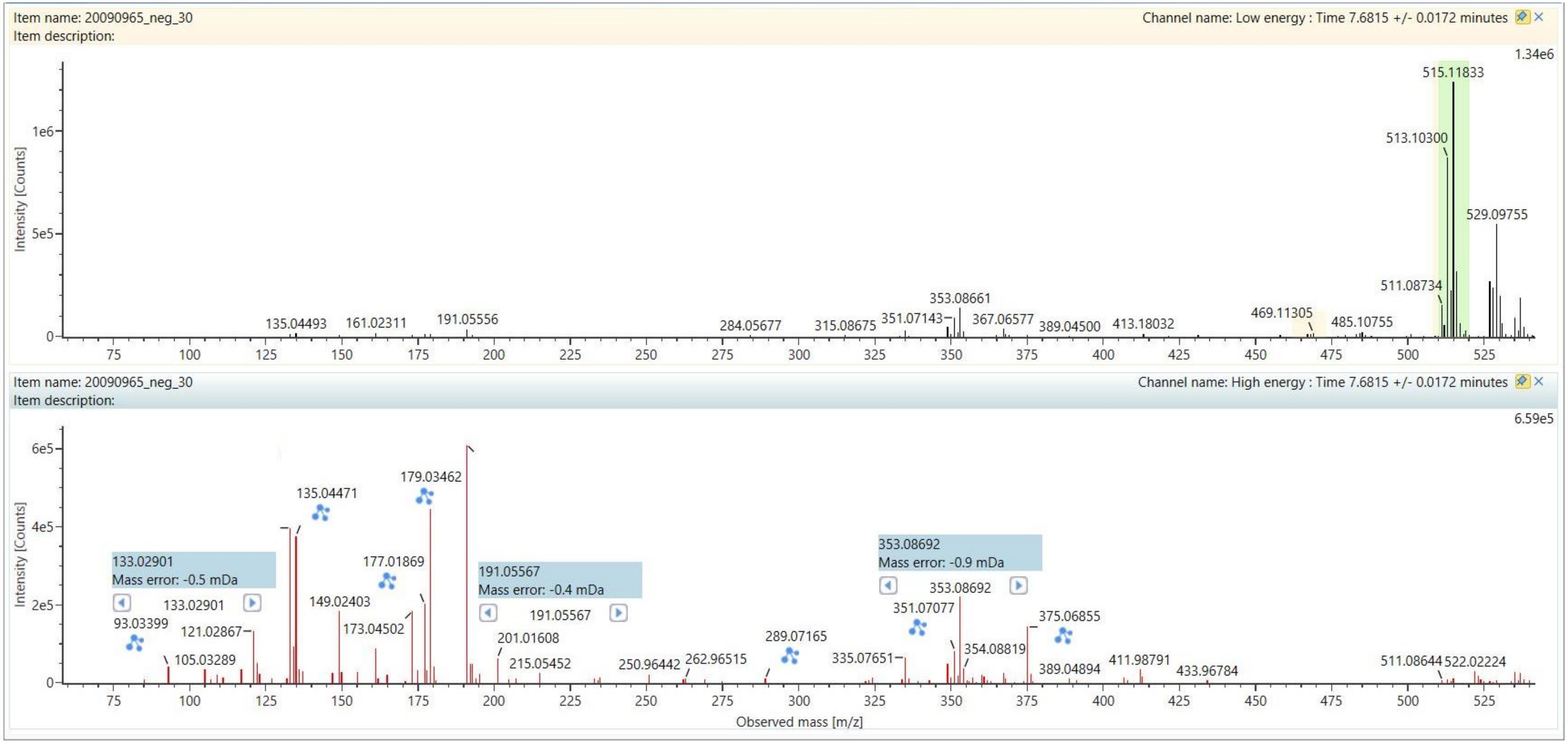

Figure S29. ESI-QTof-MS spectrum of sulfuretin (peak 28)

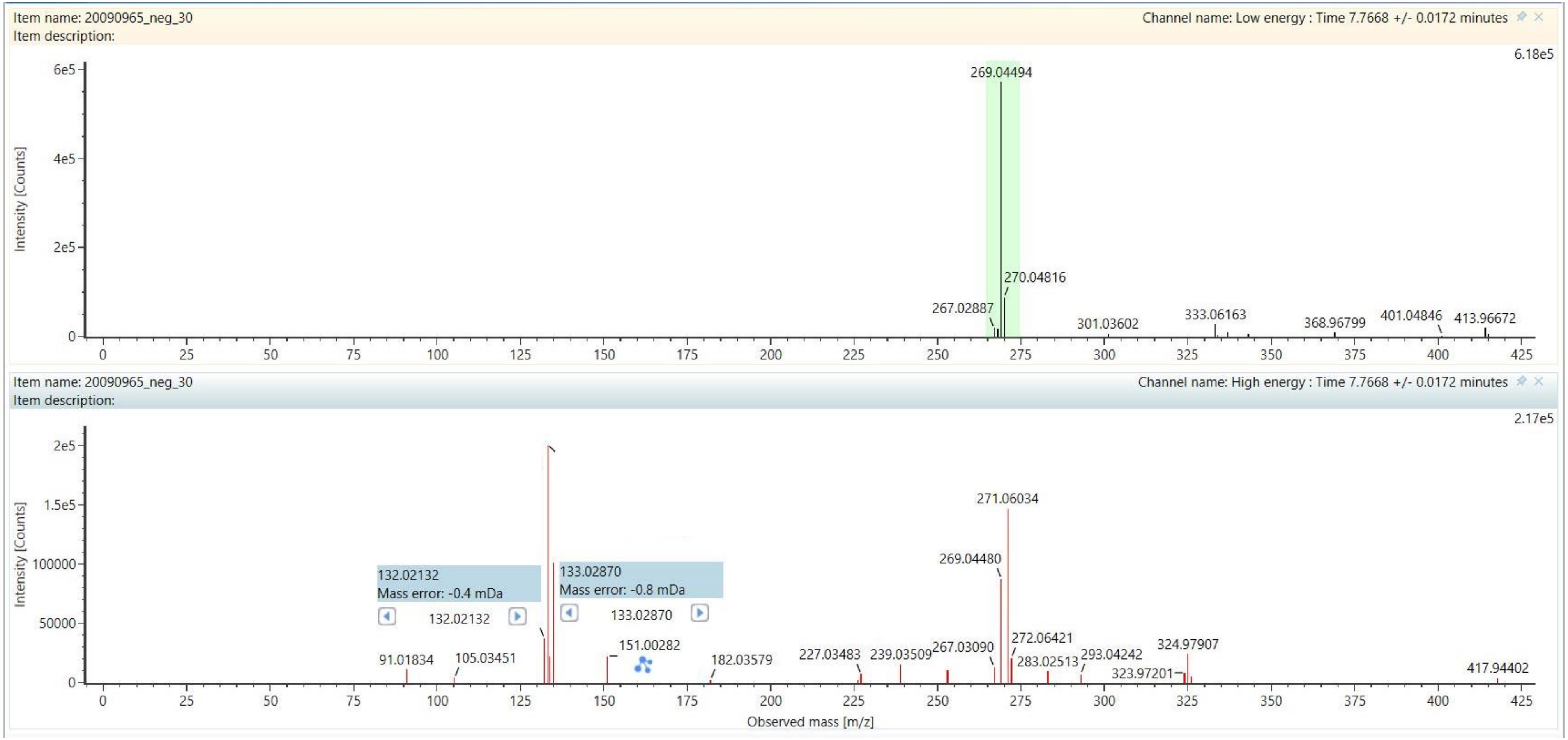

Figure S30. ESI-QTof-MS spectrum of luteolin-6-O-rhamnoside (peak 29)

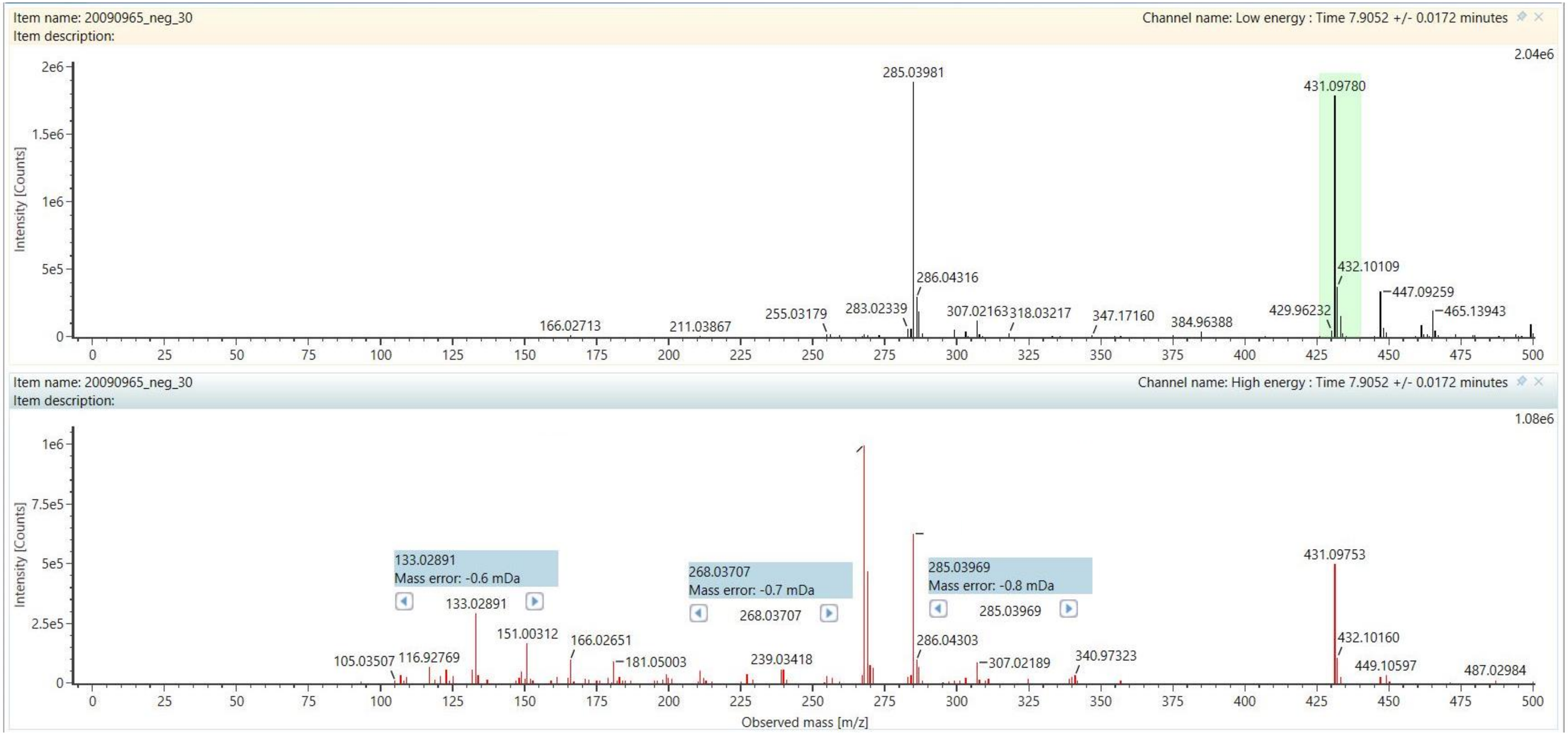

Figure S31. ESI-QTof-MS spectrum of coreopsin (peak 30)

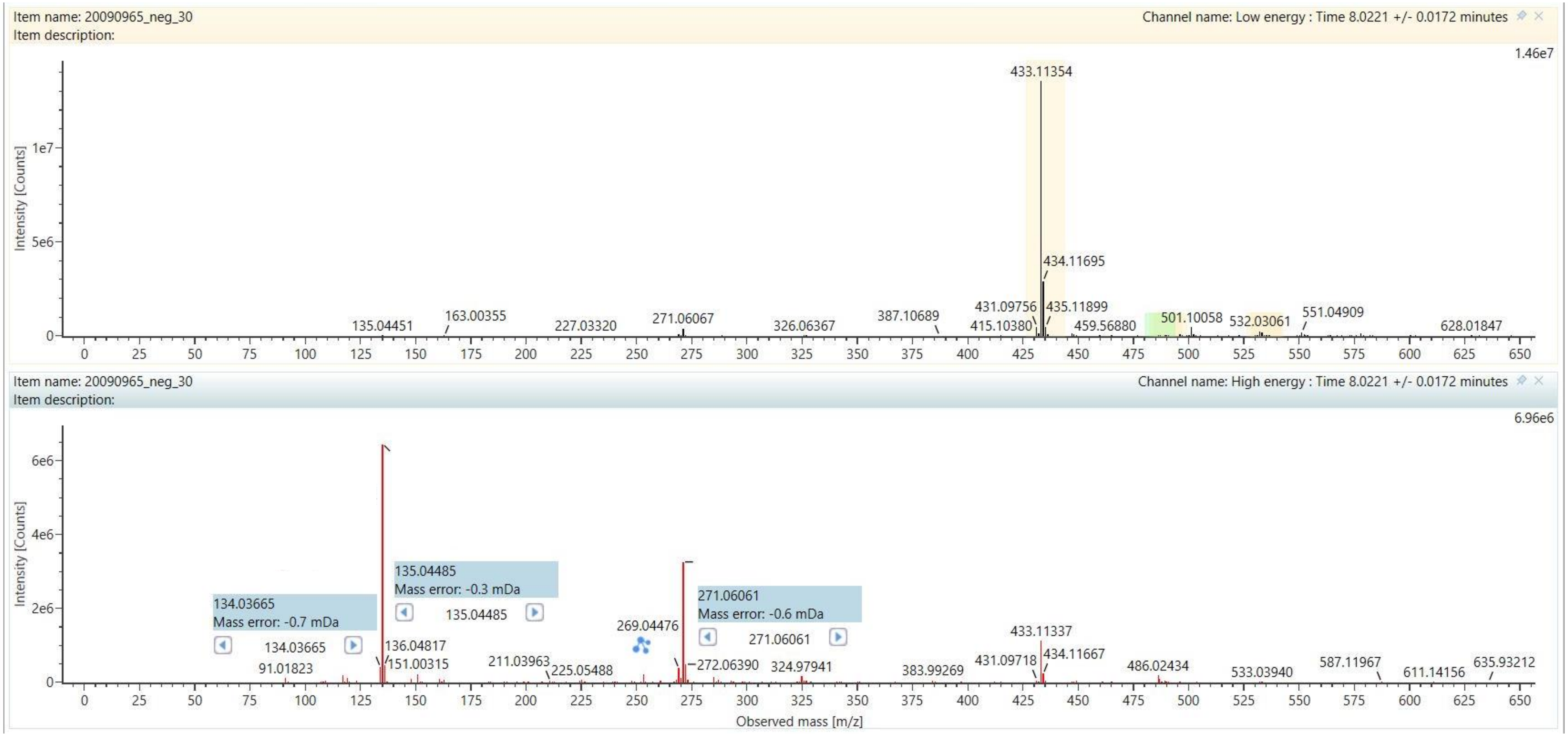

Figure S32. ESI-QTof-MS spectrum of 4,5-dicaffeoylquinic acid (peak 31)

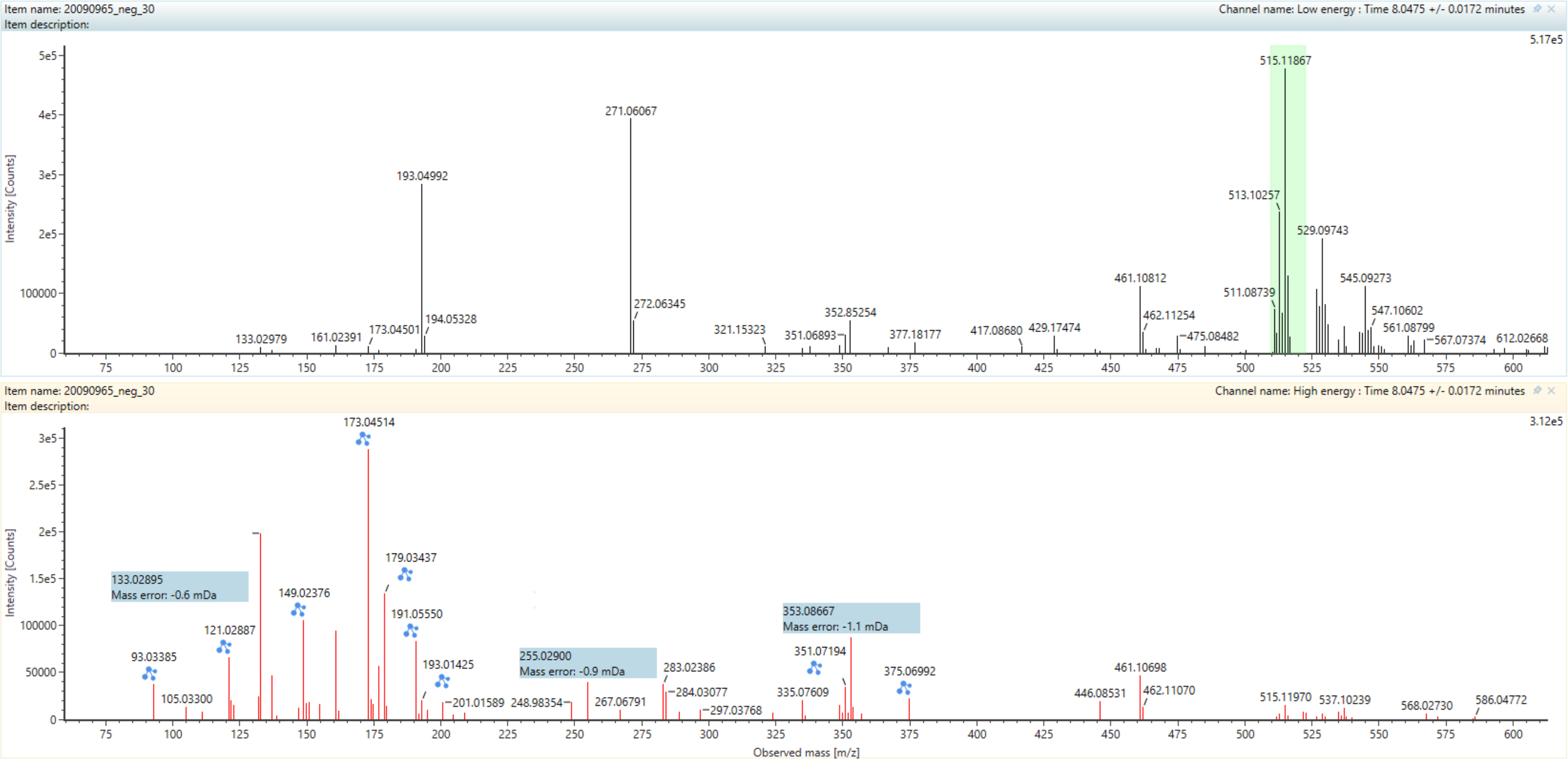

Figure S33. ESI-QTof-MS spectrum of okanin (peak 32)

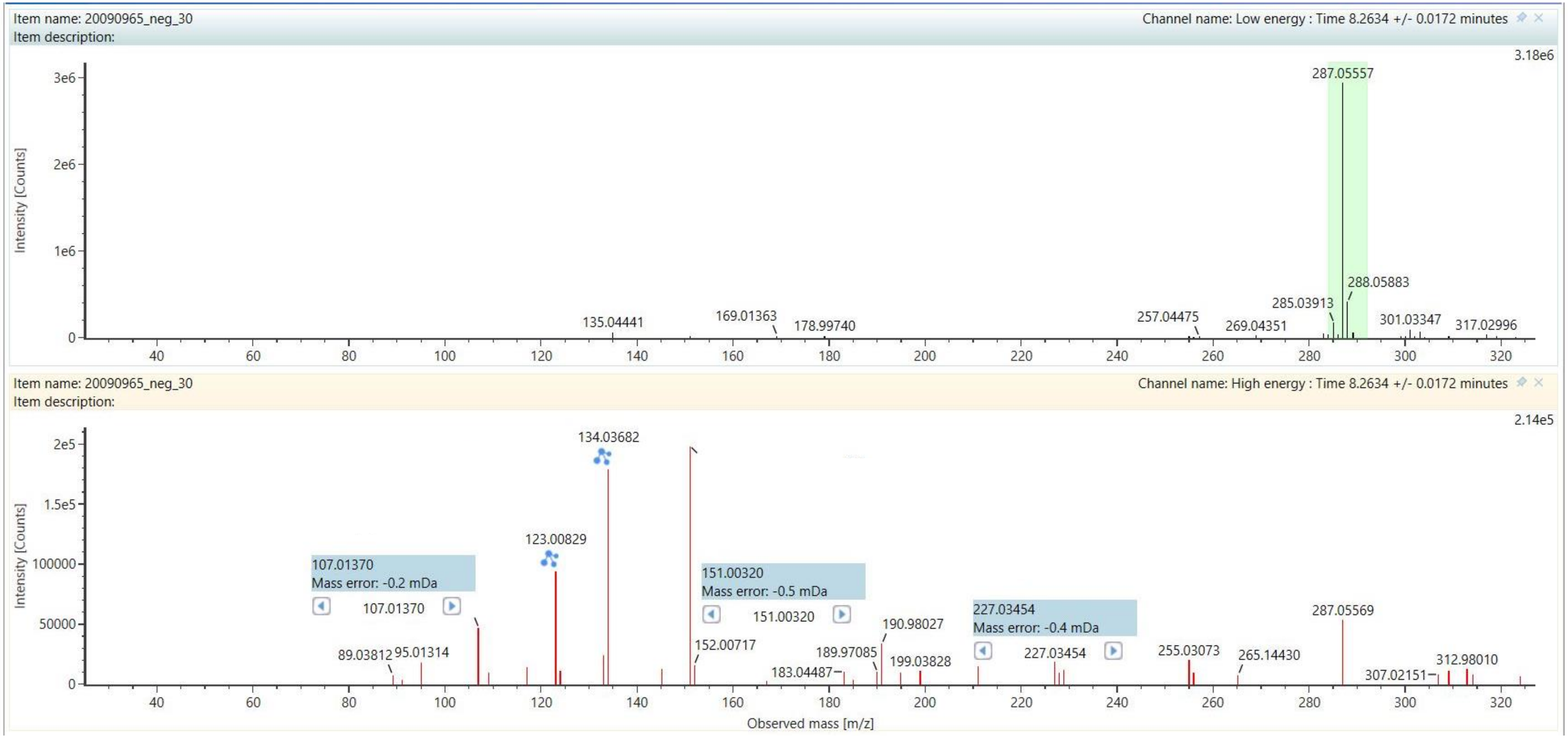

Figure S34. ESI-QTof-MS spectrum of eriodictyol chalcone-O-diglucoside (peak 33)

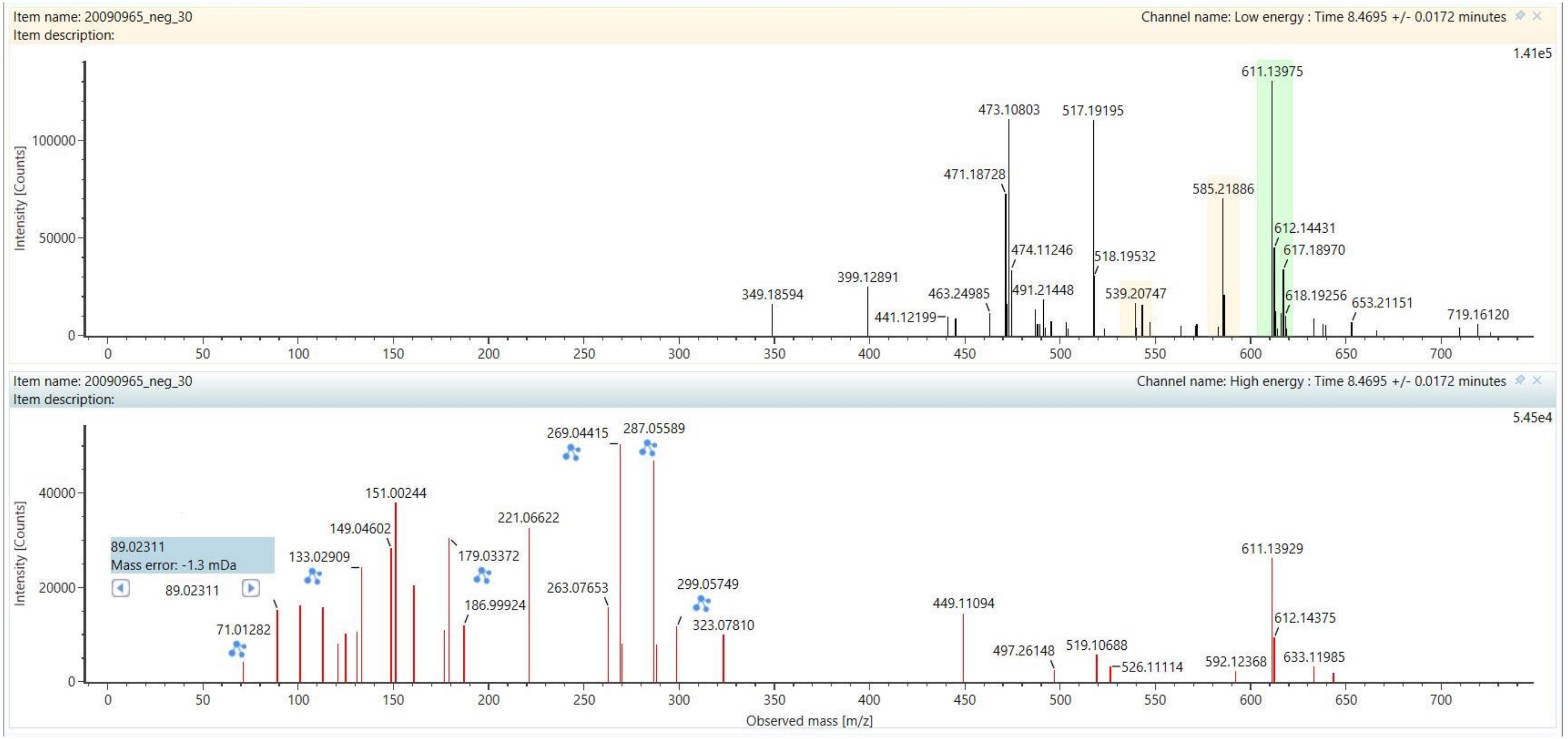

Figure S35. ESI-QTof-MS spectrum of eriodictyol chalcone (peak 34)

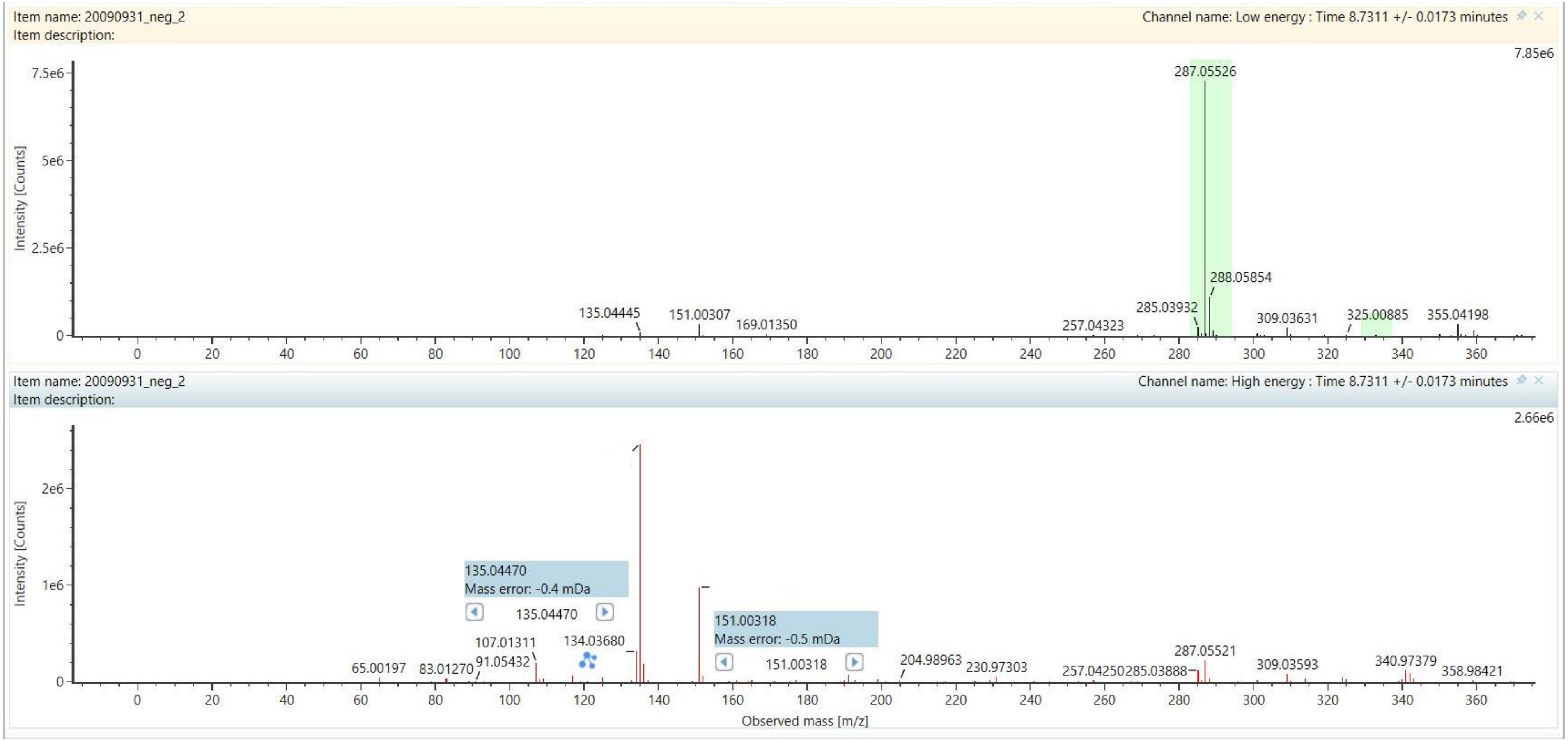

Figure S36. ESI-QTof-MS spectrum of kaempferide (peak 35)

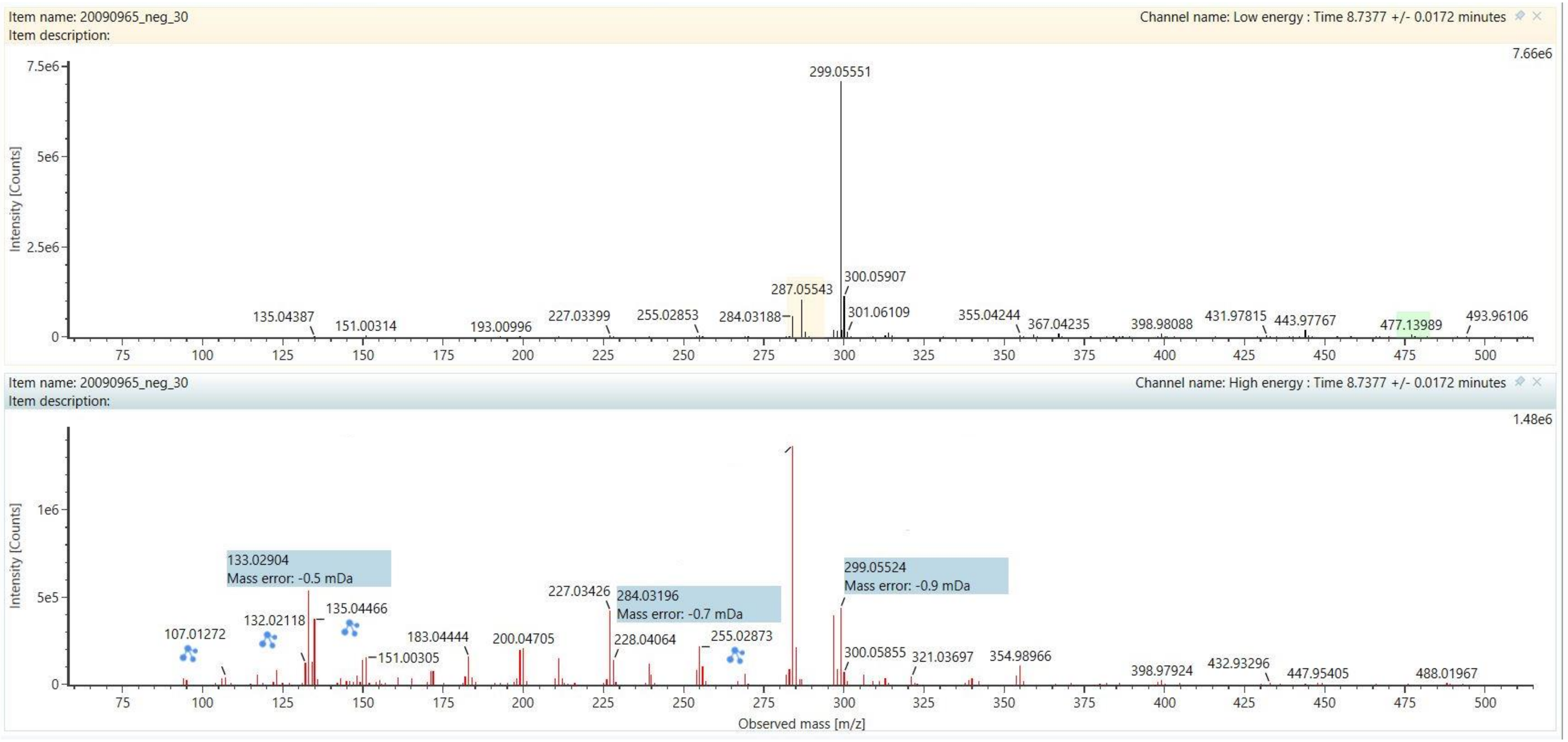

Figure S37. ESI-QTof-MS spectrum of luteolin (peak 36)

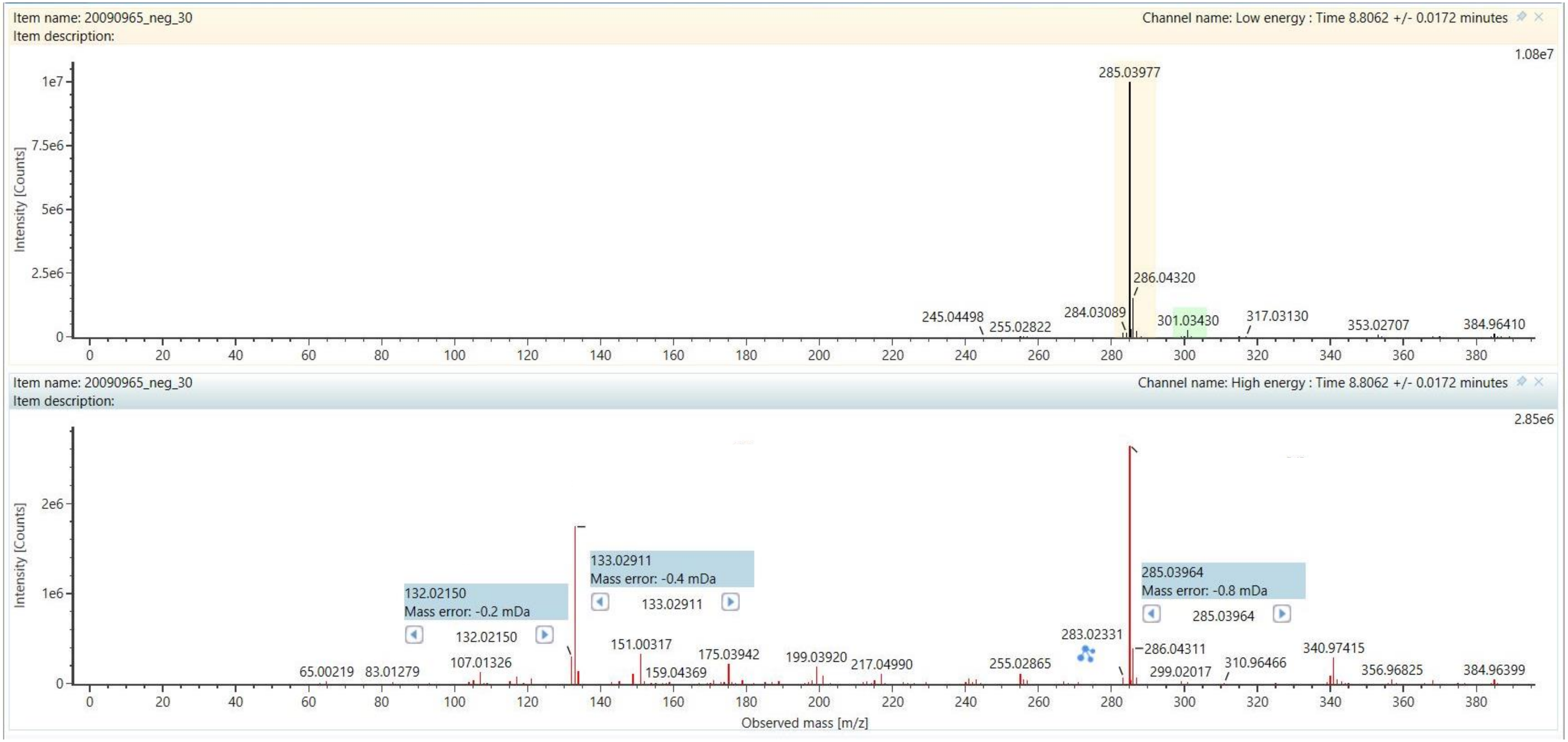

Figure S38. ESI-QTof-MS spectrum of 4-methoxylanceoletin-4′-O-glucoside (peak 37)

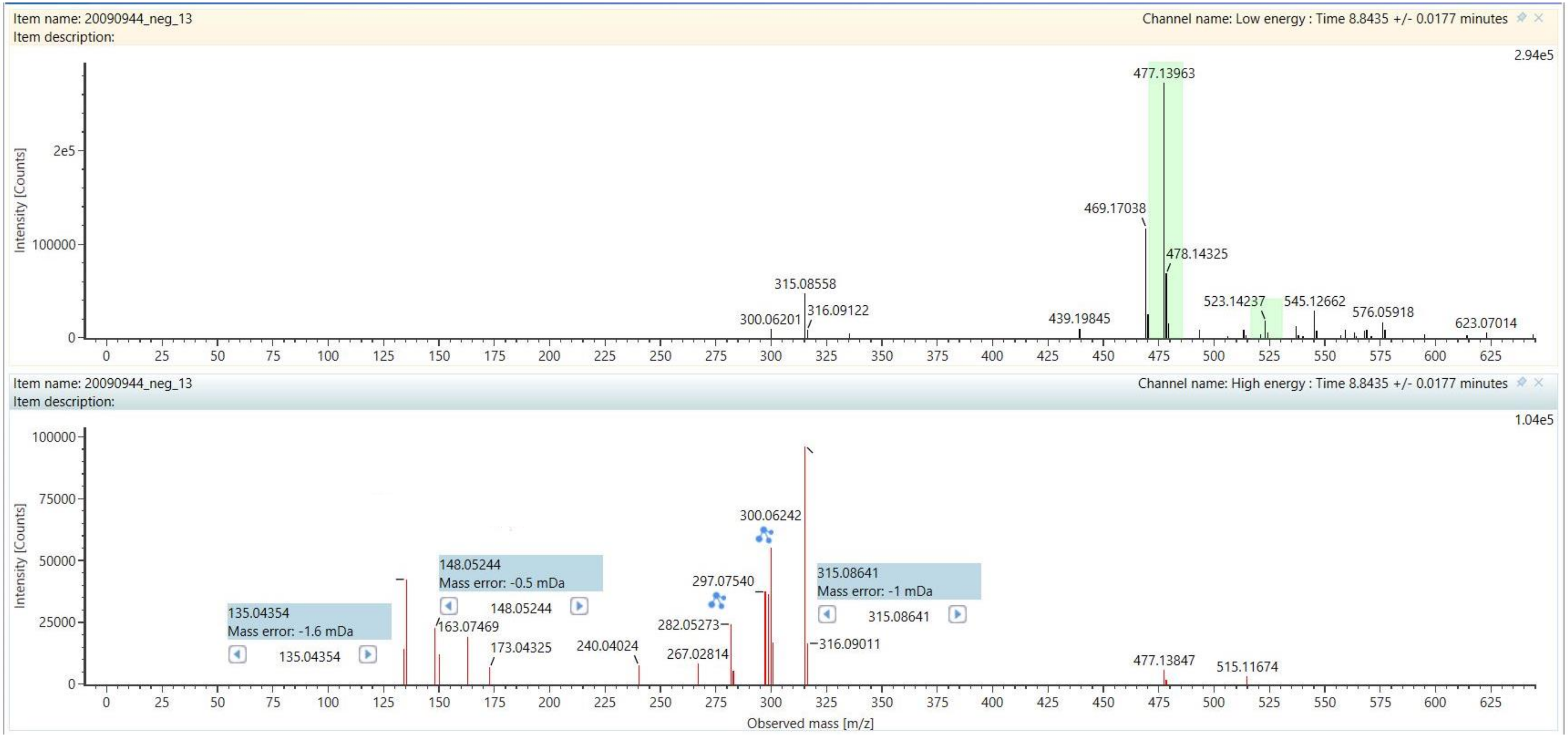

Figure S39. ESI-QTof-MS spectrum of butein (peak 38)

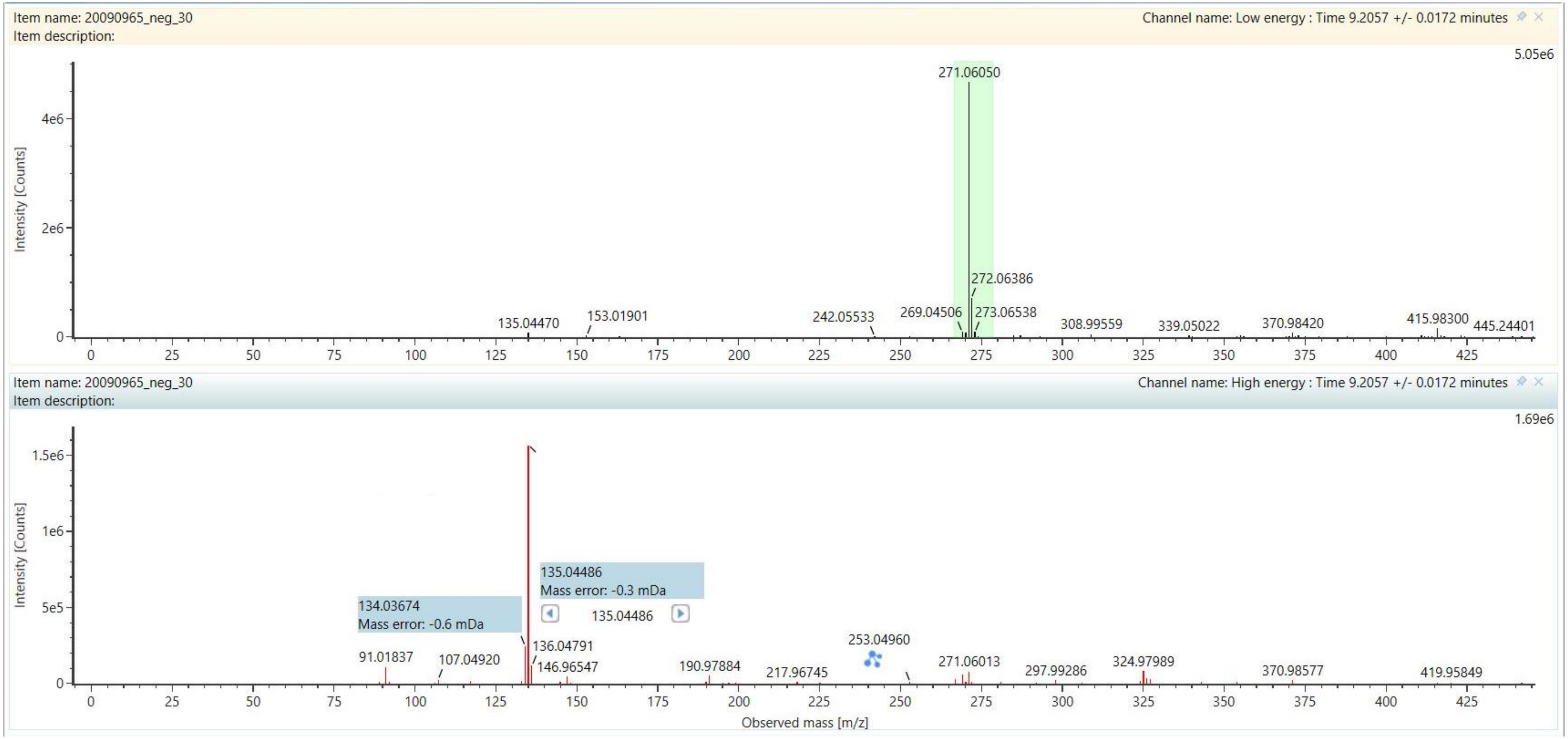

Figure S40. ESI-QTof-MS spectrum of apigenin (peak 39)

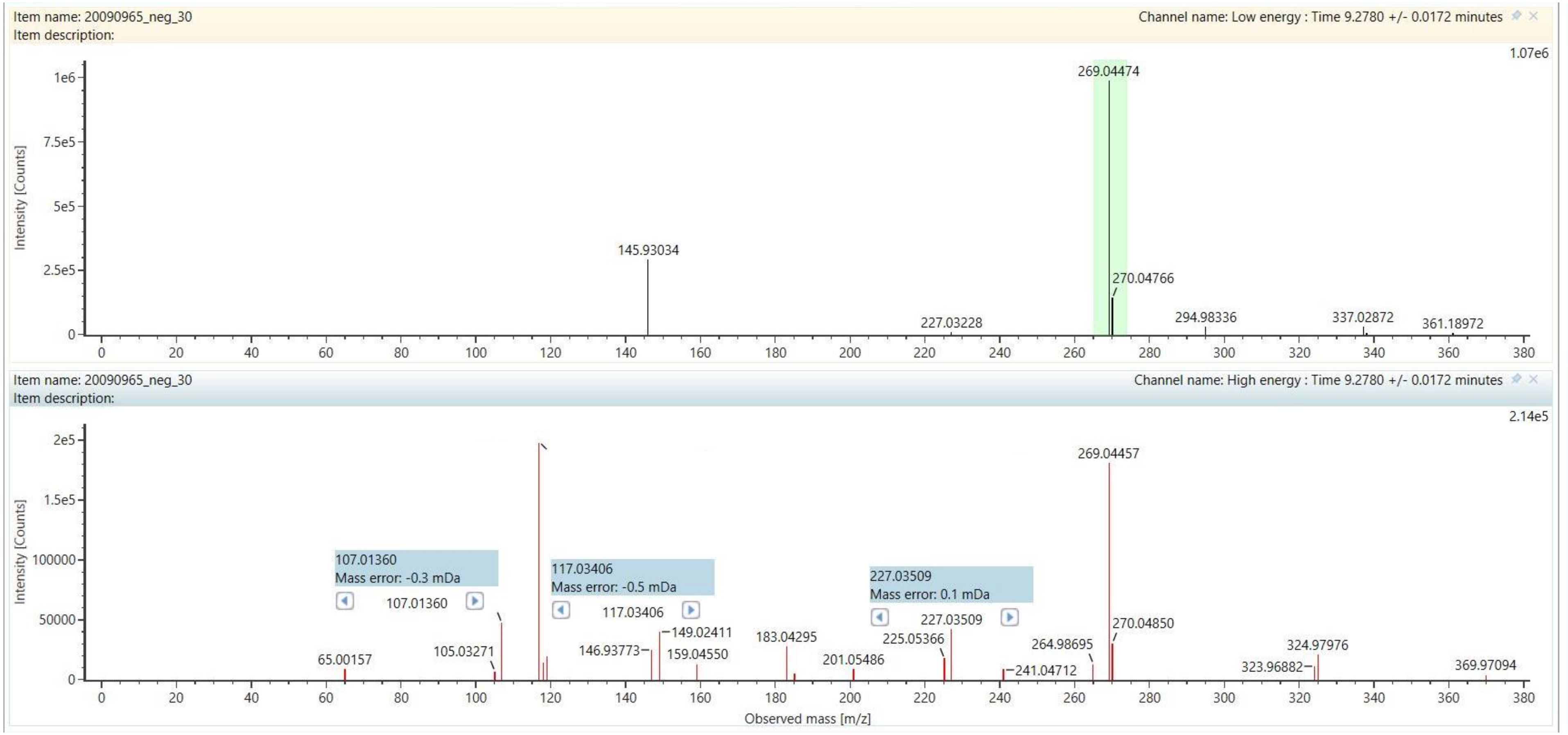

Figure S41. ESI-QTof-MS spectrum of unknown (peak 40)

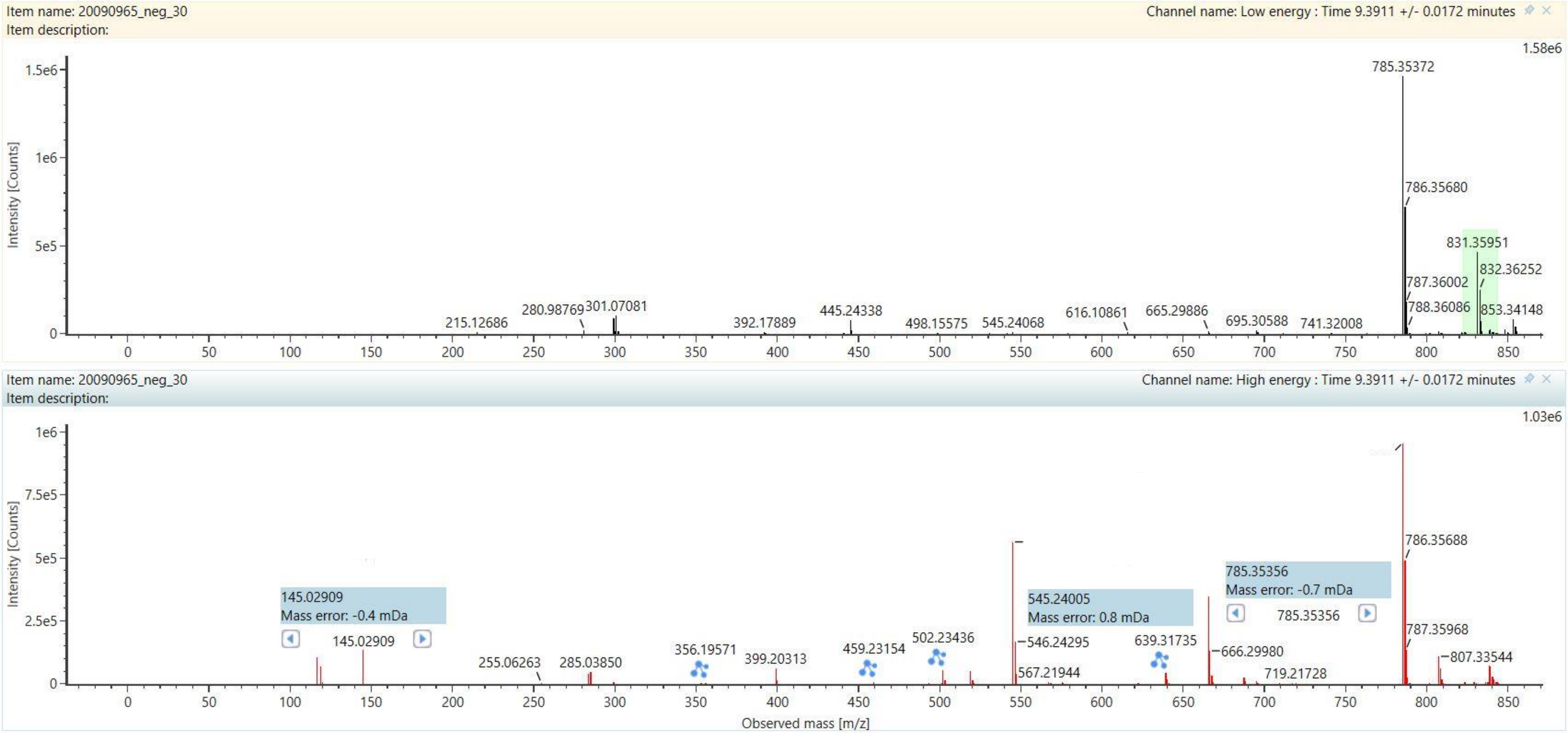

Figure S42. ESI-QTof-MS spectrum of lobetyolinin (peak 41)

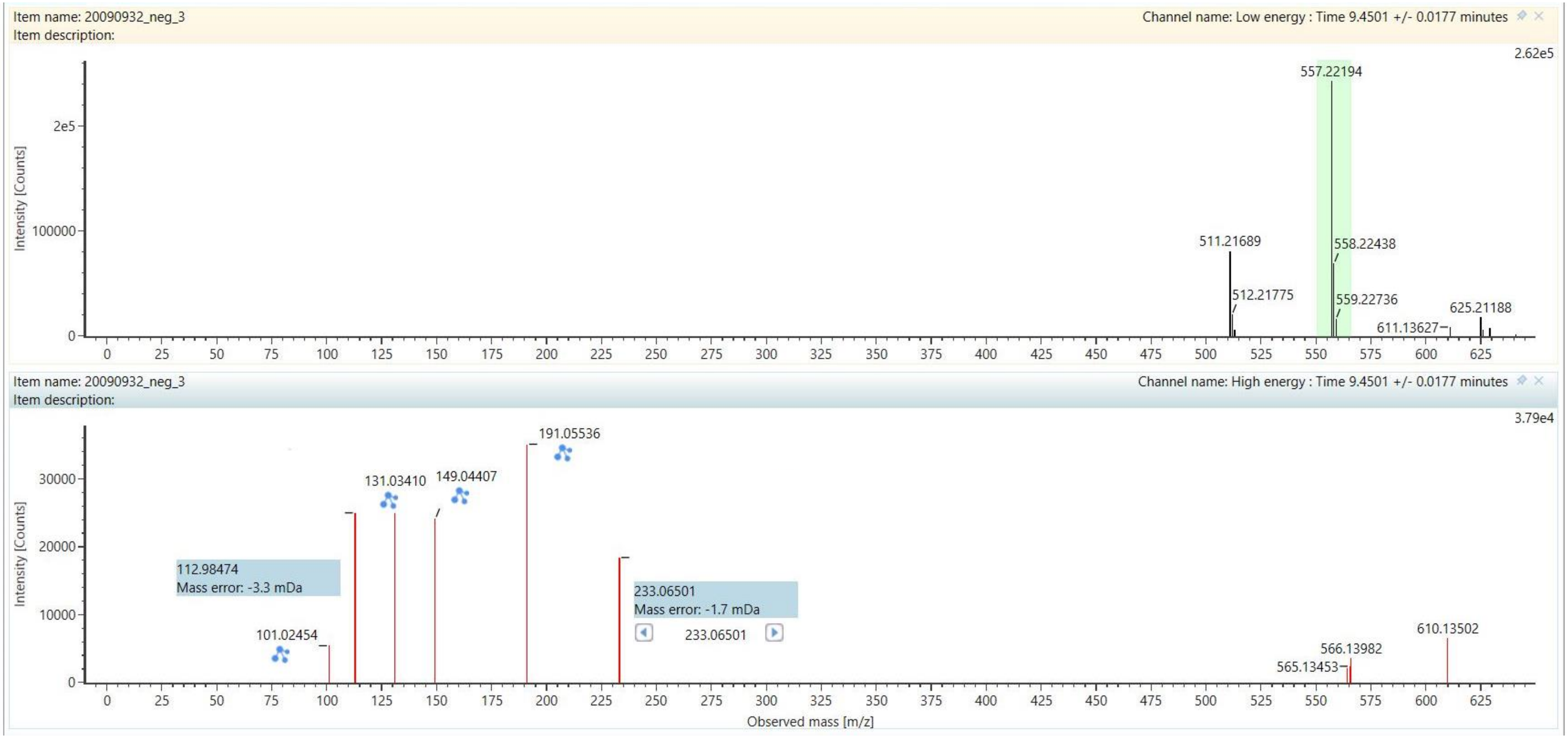

Figure S43. Validation plot of the OPLS-DA obtained from 200 permutation test.

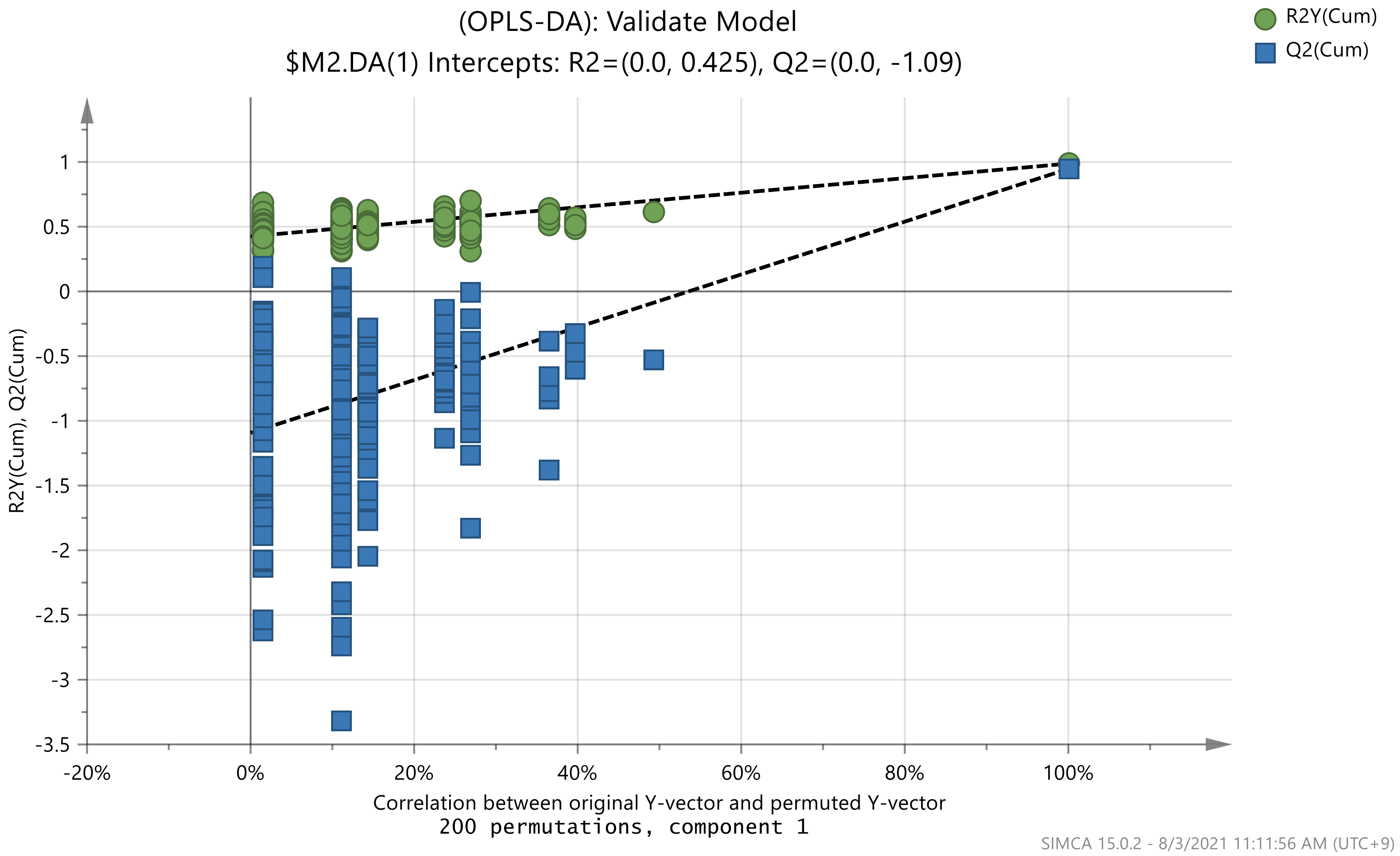

Supplement: Supplementary file 1 [file plants-10-01661-s001.zip › plants-1327139-supplementary.pdf]
